# KDM7A-DT induces genotoxic stress, tumorigenesis, and progression of p53 missense mutation-associated invasive breast cancer

**DOI:** 10.3389/fonc.2024.1227151

**Published:** 2024-05-02

**Authors:** Antonis Giannakakis, Margaritis Tsifintaris, Vasileios Gouzouasis, Ghim Siong Ow, Mei Yee Aau, Csaba Papp, Anna V. Ivshina, Vladimir A. Kuznetsov

**Affiliations:** ^1^ Department of Molecular Biology and Genetics, Democritus University of Thrace, Alexandroupolis, Greece; ^2^ Bioinformatics Institute, Agency for Science, Technology and Research (A*STAR), Singapore, Singapore; ^3^ University Research Institute for the Study of Genetic & Malignant Disorders in Childhood, National and Kapodistrian University of Athens, Athens, Greece; ^4^ Department of Urology, The State University of New York (SUNY) Upstate Medical University, Syracuse, NY, United States; ^5^ Department of Biochemistry and Molecular Biology, The State University of New York (SUNY) Upstate Medical University, Syracuse, NY, United States

**Keywords:** KDM7A-DT, oxidative stress, breast cancer, genotoxic stress, TP53 mutation, DNA damage response and repair, epithelial-to-mesenchymal transition, lncRNA alteration

## Abstract

Stress-induced promoter-associated and antisense lncRNAs (si-paancRNAs) originate from a reservoir of oxidative stress (OS)-specific promoters via RNAPII pausing-mediated divergent antisense transcription. Several studies have shown that the KDM7A divergent transcript gene (*KDM7A-DT*), which encodes a si-paancRNA, is overexpressed in some cancer types. However, the mechanisms of this overexpression and its corresponding roles in oncogenesis and cancer progression are poorly understood. We found that *KDM7A-DT* expression is correlated with highly aggressive cancer types and specific inherently determined subtypes (such as ductal invasive breast carcinoma (BRCA) basal subtype). Its regulation is determined by missense *TP53* mutations in a subtype-specific context. *KDM7A-DT* transcribes several intermediate-sized ncRNAs and a full-length transcript, exhibiting distinct expression and localization patterns. Overexpression of *KDM7A-DT* upregulates TP53 protein expression and H2AX phosphorylation in nonmalignant fibroblasts, while in semi-transformed fibroblasts, OS superinduces KDM7A-DT expression in a TP53-dependent manner. KDM7A-DT knockdown and gene expression profiling in *TP53*-missense mutated luminal A BRCA variant, where it is abundantly expressed, indicate its significant role in cancer pathways. Endogenous over-expression of KDM7A-DT inhibits DNA damage response/repair (DDR/R) via the TP53BP1-mediated pathway, reducing apoptosis and promoting G2/M checkpoint arrest. Higher *KDM7A-DT* expression in BRCA is associated with *KDM7A-DT* locus gain/amplification, higher histologic grade, aneuploidy, hypoxia, immune modulation scores, and activation of the c-myc pathway. Higher KDM7A-DT expression is associated with relatively poor survival outcomes in patients with luminal A or Basal subtypes. In contrast, it is associated with favorable outcomes in patients with HER2+ER- or luminal B subtypes. KDM7A-DT levels are coregulated with critical transcripts and proteins aberrantly expressed in BRCA, including those involved in DNA repair via non-homologous end joining and epithelial-to-mesenchymal transition pathway. In summary, *KDM7A-DT* and its si-lncRNA exhibit several intrinsic biological and clinical characteristics that suggest important roles in invasive BRCA and its subtypes. *KDM7A-DT*-defined mRNA and protein subnetworks offer resources for identifying clinically relevant RNA-based signatures and prospective targets for therapeutic intervention.

## Background

Stress-inducible promoters are forged by evolution to adapt cells during acute and chronic stress responses. LncRNAs are regulatory ncRNAs with a size greater than 200 nucleotides and without significant coding potential. They play several roles, including cell homeostasis, metabolism, and response adaptation pathways. A systems-level approach was previously used to characterize a sharp burst in noncoding transcription immediately upon oxidative stress (OS), associated with a novel stress-induced, bi-directional RNAPII accumulation and pausing phenomenon at divergent promoters and enhancers (1). Results of this study have revealed that this phenomenon was enhanced in active and stress-inducible promoters, which transcribe well-annotated lncRNAs, called the promoter-associated antisense lncRNAs (si-paancRNAs) (1). The above phenomenon was later shown to occur as early as 15 minutes following 0.3 mM H_2_O_2_ treatment (2). This increase in RNAPII-mediated divergent transcription may serve as a natural regulatory mechanism of transcriptional and translational interference (3, 4) since si-paancRNAs showed an increased association in all RNP fractions from free-RNPs to polysomes (1). We reasoned that this stress-induced bidirectional transcription phenomenon represents a hidden regulatory mechanism of the core stress response, linking genotoxic stress to transcriptional/replication stress at stress-specific promoters and the acclimation of the cellular RNP environment for the cell to make the survival switch from proliferation or development to stress protection/adaptation (5). A global non-coding response to OS agrees with a stress-induced non-coding system identified in yeast correlated with decreased protein levels (6).

How cells adapt to their environment, particularly under stressful conditions, is critical for survival and is dictated by the core stress response (CSR). Both pillars of the CSR, DDR/R, and protein damage response (PDR), are thought to be regulated mostly post-transcriptionally *via* RNA turnover and translation (7), even though a small number of immediate-early genes are induced by mRNA transcription (8) OS is one of the primary cell stressors caused by an imbalance between the production and elimination of reactive oxygen species (ROS). OS activates the DNA and protein damage response signaling pathways, resulting in cell cycle arrest and death or metabolic adaptation (9). OS-induced damage primarily consists of DNA lesions in the noncoding genome, which can block genomic replication and transcription (5). Although not lethal, these lesions are highly mutagenic, especially during chronic oxidative DNA damage (10) due to a process known as the Fenton-type reaction, which creates base lesions, leading to single-strand or double-strand DNA breaks (SSBs and DSBs, respectively) (11). OS is involved in the induction and progression of several cancers, including BRCA. BRCA cells present increased ROS production and elevated antioxidant defense, balancing oxidative status (12).

Damaged nucleic acids and oxidized bases must be immediately and precisely repaired, or they can cause replication stress, genomic instability, inflammation, and cancer (13). The branch of the DDR/R responsible for repairing stress-induced DSBs is mainly via the non-homologous end-joining (NHEJ) branch of DDR/B, which ligates blunt DNA ends or DSBs with short overhangs and has low reproducibility. In contrast, homology-directed recombination (HR) represents the other DDR/R branch, which shows increased reproducibility and is highly conserved. Nevertheless, a template with high sequence homology to the broken DNA ends (or long overhangs) is required to repair DNA damage. Hence, HR is the dominant DNA repair mechanism during the mitotic phase of the cell cycle. At the molecular level, a DSB activates the DNA damage response kinases ATM, ATR, and DNA-PKcs, which phosphorylate Ser139 in the C-terminal tail of the histone variant H2AX. Upon H2AX phosphorylation (γ-H2AX), DSBs can be restored by NHEJ, which involves TP53BP1 recruitment to chromatin, or by HR, most frequently via the BRCA1/2–RAD51 pathway (14). TP53BP1 is a DNA damage response factor that interacts with the TP53 protein and is a driver protein in the cell-cycle-dependent DSB repair pathway (15).

The protein encoded by *TP53* is a master regulator of the stress response, controlling the cell cycle and inducing apoptosis. As one of the most prominent tumor suppressor proteins, TP53 acts at the transcriptional level as a pro-apoptotic factor in a transcription-independent level *via* pathways originating from the mitochondria or cytosol, directly causing cell death (16, 17). In addition, TP53 responds to multiple stressors, such as OS, DNA damage, hypoxia, nutrient starvation, and replicative stress, inducing the transcription of DDR/R pathway proteins, which in turn leads to genome instability, causing cell cycle arrest, autophagy, senescence, apoptosis, ferroptosis, metabolic reprogramming, and inhibition of stemness (18, 19). The mechanisms underlying the interplay between TP53, cell fate decisions, and cancer are still under investigation (20). In fact, under normal circumstances, TP53 is rapidly degraded, with a half-life of 20 minutes. At the same time, after exposure to DNA-damaging agents, its levels increase through posttranslational modifications and binding to the TP53-response element, stabilizing the protein (21, 22). Wild-type TP53 activity is typically linked to a favorable prognosis in cancer, although its mutation status and context should also be considered in clinical practice (23). Mutations in *TP53* are essential for BRCA initiation and progression (24). About 33% of human BRCA cases (based on cBioportal and TCGA 1084 samples) have a mutated TP53. The *TP53* mutation status across BRCA is subtype-specific. As reported in The Cancer Genome Atlas (TCGA) data, *TP53* mutations occur in 10.9% of luminal A, 39.3% of luminal B, 69.3% for HER2, and 88.4% of basal BRCA subtypes, the majority of which are missense affecting the DNA-binding domain (25).

*TP53* mutations are commonly involved in epithelial-to-mesenchymal transition (EMT)-associated pathways. EMT allows an epithelial cell to switch differentiation programs to a mesenchymal one. EMT-associated pathways are essential in physiological and pathological events, such as embryogenesis, wound healing, and oncogenesis. EMT-associated genes are classified into three diverse types (26, 27): type 1 is associated with embryogenesis and organ development; type 2 is associated with wound healing, tissue regeneration, and organ fibrosis; and type 3 is associated with cancer development. In all cases, the mesenchymal cell phenotype includes enhanced migration, stem cell-like capacity, invasiveness, elevated resistance to apoptosis, and significantly increased production of extracellular matrix components.

LncRNAs are immediately upregulated upon stress and regulate cellular responses to oxidative, metabolic, endoplasmatic reticulum, and genotoxic stress *via* multiple RNA-RNA, RNA–protein, RNA–DNA, and RNA–DNA–protein interactions (1, 28–31). Specific lncRNAs seem to be part of the TP53-mediated stress response pathway. Indeed, among the hydrogen peroxide (H_2_O_2_)-modulated lncRNAs in cells, MEG3 activates TP53, altering stress and chemotherapy responses; the MALAT1-mediated proliferative capacity is directly affected by TP53 activity (30–32); GUARDIN (a TP53-responsive lncRNA) maintains genomic integrity under steady-state conditions and after exogenous genotoxic stress exposure (33). The list of lncRNAs involved in the TP53 pathway of DDR/R is rapidly expanding. However, the exact role played by TP53 might be context-dependent (30, 32, 33). In parallel, the prevailing view that DDR/R mainly involves proteins and subsequent protein modifications, both of which promote the accumulation of DDR/R factors and form nuclear DDR foci, is being challenged by the recent paradigmatic shift in our understanding of the RNA-mediated regulatory mechanisms in hallmark pathways of the cellular stress response. Specifically, besides si-paancRNAs and other regulatory classes of si-lncRNAs that were discovered by our group (see below), numerous damage-induced lncRNAs (dilncRNAs) generated by RNA polymerase II (RNAPII) from the DNA ends of DSBs have also been discovered (34). The dilncRNAs form double-stranded RNAs (dsRNAs), which, similar to miRNA biogenesis, are further processed by DICER and DROSHA to generate DNA damage response precursors and small RNAs (DDRNAs) (35). TP53BP1 interacts with DDRNAs and dilncRNAs, potentially recruiting TP53BP1 to the DNA damage site (36).

Stress-induced promoter-associated and antisense lncRNAs (si-paancRNAs) are a class of si-lncRNAs that were discovered to be upregulated transiently upon physiological acute OS from a reservoir of stress-specific promoters via RNPII pausing-mediated divergent antisense transcription (1–5). We argue that si-paancRNAs and possibly other classes of si-lncRNAs represent a «hidden» or context-specific regulatory layer of the core stress response. Several studies have shown that the KDM7A divergent transcript (KDM7A-DT), which encodes a si-paancRNA, is overexpressed in cancer cells. The si-paancRNA KDM7A-DT was recently reported i) to aggravate the development of gastric cancer through the miR-450a-2-3p–PRAF2 axis (37); ii) to promote tumor growth of pancreatic cancer cell lines by regulating angiogenesis in response to nutrient starvation (38); iii) to represent a prognostic biomarker in non-small-cell lung cancer growth and metastasis by interacting with DHX15 protein (39); iv) to be one of the top differentially expressed lncRNAs in esophageal squamous cell carcinoma (40); v) to play a protective role in ROS-induced apoptosis of periodontal ligament stem cells by decreasing DNAJC10 *via* phosphorylation of the eIF2a protein (41); vi) to be one of the differentially expressed lncRNAs in whole maternal blood that correlate with advancing gestation in normal pregnancy (42); vii) to be used as disease-free survival (DFS) prognostic marker in patients with gastric cancer in combination with 12 other lncRNAs (43); viii) to alleviate neuroinflammation and neuronal injury *via* the miR-101-3p–DUSP1 axis in the spinal cord after brachial plexus injury (44); ix) to suppress MPP+-induced neuronal damage in Parkinson’s disease *via* the miR−134−5p–PIK3R3 axis (45); and x) to induce breast carcinogenesis by inhibiting miR-940 (KDM7A-DT-miR-940-ARTN ceRNA network) (46). The above results suggest that KDM7A-DT may exert its pathogenic functions through genetic changes and overexpression, leading to specific abnormal DNA, RNA, and protein interactions in cell type-specific contexts. However, no studies examine KDM7A-DT’s role in linking stress-induced pathways to cancer.

Overexpression of four (4) si-paancRNAs resulted in cell cycle checkpoint arrest and activation of the DDR/R and TP53 signaling pathways in nonmalignant fibroblasts. Following an unbiased functional screening of these si-paancRNAs in the parental, *TP53*, and p16/*INK4* semi-transformed fibroblast cell lines, KDM7A-DT was selected for (i) its highest induction levels upon OS in non-malignant cells and (ii) its superinduction by OS following the inhibition of TP53 mRNA levels, in semi-transformed fibroblasts. KDM7A-DT over-expression was significantly associated with an overall pro-oncogenic transcriptional signature in non-malignant lung fibroblasts. Based on a comprehensive NGS and clinical data analysis in TCGA BRCA patients, a functional link was established between KDM7A-DT overexpression and an aggressive cancer cellular phenotype associated with genome instability (GI), copy number alterations (CNAs), aneuploidy, *TP53* mutational status, and critical BRCA-associated transcriptional pathways.

Despite progress in the field, the mechanism(s) driven by over-/under-expression of KDM7A-DT in genotoxic stress response, specific pathways, oncogenesis, and cancer progression are poorly understood. In particular, in clinically relevant BRCA subtypes, the role of the pro-oncogenic or tumor suppressor functions of KDM7A-DT has not been identified.

A key objective of our study is to identify the functional isoform(s), expression profiles, amplification events, aberrant gene expression mechanisms, and pathways of KDM7A-DT during tumorigenesis and progression of p53 mutation-associated invasive breast cancer subtypes. To accomplish this, we developed a cancer systems biology approach, which includes molecular and cellular biology methods, cell line models, and bioinformatics methods. According to our findings, the KDM7A-DT locus and its full-length transcript play multiple roles in genotoxic stress, hormonal-independent tumor formation, and progression of TP53 missense mutation-associated BRCA subtypes. This paper’s experimental results and clinical data analyses suggest diverse roles for *KDM7A-DT* and its lncRNA in BRCA subtypes pathobiology and clinical variant outcomes.

## Results

### Constitutive upregulation of si-paancRNAs in nonmalignant fibroblasts induces DDR/R signaling and stress response–related growth retardation

**Figure 1:**
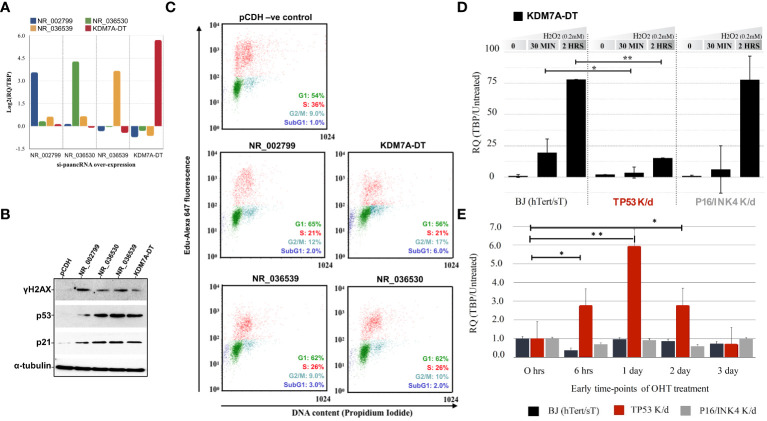
Overexpression of selected stress-induced promoter-associated lncRNAs (si-paancRNAs) in normal lung fibroblasts triggers cell cycle arrest and activates DDR/R signaling **(A)**. The average transcript levels of the four chosen si-paancRNAs when overexpressed in MRC5 cells (using a lentivirus approach) compared with cells expressing just the vehicle control (pCDH). **(B)** Overexpression of all four si-paancRNAs significantly increased the protein levels of p53 and CDK1A and the phosphorylation levels of the hallmark DDR/R protein H2AX compared with those in control cells. **(C)** Representative cell cycle analysis scoring for DNA content and EdU Alexa Fluor 647 showed that MRC5 cells overexpressing the four different si-paancRNAs entered the S phase significantly slower than MRC5 control cells (pCDH) in three independent flow cytometry experiments. **(D)** KDM7A-DT RNA levels were super-induced in a p53-dependant manner in response to acute OS (0.2 mM H_2_O_2_, 30 minutes), as analyzed by qPCR in modified BJ fibroblasts with constitutive *TP53* and P16/*INK4* knockdown compared with control modified cells. **(E)** KDM7A-DT was the only si-paancRNA upregulated significantly by early *HRAS* oncogenic activation (OHT treatment) in *TP53* knockdown BJ cells compared with P16/*INK4* knockdown and untreated BJ cells. P-values less than 0.05 are summarized with one asterisk (*), and P-values less than 0.01 are summarized with two asterisks (**).

Among the validated si-paancRNAs (at all time points and cell lines) studied in our previous study (1), we selected si-paancRNAs that showed significantly different transcript abundance within the non-translating/free-RNP fraction compared with the translating polysome fraction (30). We used a lentivirus-based approach to elucidate the noncoding roles of si-paancRNAs in nonmalignant cells. We analyzed stable gain-of-function phenotypes (47, 48) in MRC5 cells of four si-paancRNAs (NR_036539, NR_002799, NR_036530, and NR_024451 [KDM7A-DT]) compared with a nontargeting-empty control vector (pCDH) (**Figure 1A**). Lentivirus-transduced cells were subjected to puromycin selection. Consistent overexpression of all four si-paancRNAs resulted in a stress-induced premature senescence phenotype associated with growth retardation, a significant decrease in the estimated number of living cells compared with control cells, and a phenotypic transition from small, spindle-shaped cells to relatively large, flattened cells (**Supplementary Figure S1A**).

Stable overexpression of all four si-paancRNAs resulted in significant phosphorylation (*i.e.*, protein activation) of the hallmark DDR/R pathway protein γH2AX and considerable upregulation of total TP53 and CDKN1A (p21) protein levels (**Figure 1B**). We visually inspected transduction efficiency based on the GFP reporter fluorescence to confirm that the resulting phenotype of the overexpressed si-paancRNAs was not due to differences in lentivirus transduction or integration efficiency compared with the control vector. Then, we confirmed the results by quantifying the vector-derived sequences LTR-GAG and GFP using qPCR (**Supplementary Figure S1B**). When overexpressed, KDM7A-DT caused a direct block within the S phase (intra-S phase checkpoint), followed by a second accumulation of living cells in the G2 phase while increasing the percentage of apoptotic cells (sub-G1 phase). In contrast, the other three si-paancRNAs inhibited only entry into the S phase by blocking the G1/S transition, which increased cell accumulation in the G1 phase (**Figure 1C**).

### Regulation of KDM7A-DT induction by OS and early oncogenic stress is dependent on TP53 in nonmalignant semi-transformed fibroblasts

To identify which of the two main signaling pathways of the CSR regulates si-paancRNA induction, we used a modified 4-OHT-inducible *HRAS* BJ fibroblast cell line to express hTERT and small T proteins, as well as the corresponding p53 and p16/*INK4* stable knockdown cell lines (see Methods). Among the si-paancRNAs tested, KDM7A-DT was the only one to show a ‘super-induction’ in modified BJ cells under OS. Specifically, only 2 hours after H_2_O_2_ treatment, KDM7A-DT’s transcript levels increased ~80-fold compared to those in untreated cells. Furthermore, the ability of KDM7A-DT to be super-induced by OS was abolished only in the *TP53* knockdown BJ cell line (**Figure 1D**). In addition, we tested the expression of selected si-paancRNAs in response to short- and long-term oncogenic stress by overexpressing the *HRAS* oncogene by 4-OHT treatment when one of the two main stress pathways (*TP53* or p16/*INK4*) was abolished. KDM7A-DT was significantly upregulated at 6 hours, one (1) day, and two (2) days after HRAS induction in *TP53* knockdown cells compared with p16/*INK4* and control cells (**Figure 1E**). The results indicate that KDM7A-DT is super-induced by OS and that its aberrant induction levels depend on TP53 loss of function in semi-transformed human fibroblasts.

### Stress-induced alternative usage of 5’ and 3’ transcriptional ends at the KDM7A-DT locus generates novel short and long isoforms

**Figure 2:**
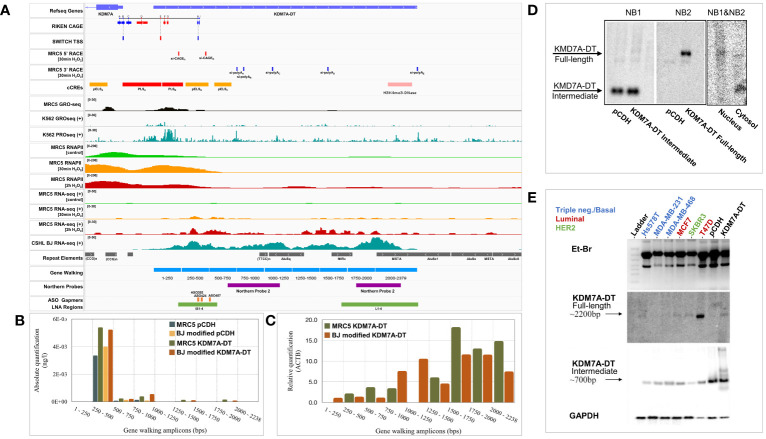
Genomic characterization of the KDM7A-DT locus and its stress-induced divergent promoter. **(A)** Visualization of genomic and experimental tracks in IGV viewer. CAGE data from the RIKEN project were downloaded from https://springernature.figshare.com/articles/dataset/annotations_of_the_human_CAGE_peaks/4797436?backTo=/collections/FANTOM5_CAGE_profiles_of_human_and_mouse_samples/3728767 and CHSL RNA-seq quantification data of BJ cells were derived from UCSC table browser (https://genome.ucsc.edu/cgi-bin/hgTables). The same source was utilized for downloading SWITCH TSS, repeat elements, and ENCODE cCREs (Candidate cis-Regulatory Elements). Regarding cCRES, red corresponds to a promoter-like signature, while orange and yellow indicate a proximal or distal enhancer. Pink corresponds to H3K4me3-enriched DNase-hypersensitive regions, while blue marks regions carrying only CTCF binding sites. GEO (Gene Expression Omnibus) database was used to retrieve GRO-seq and PRO-seq for K562 cells (GSE60456), and RNA-seq, RNApolII ChIP-seq, and GRO-seq data for MRC5 cells (GSE55169, GSE55171, GSE92375). We performed gene walking and northern blot analysis and 5′ and 3′ RACE analysis. Gene walking experiments using primer pairs hybridizing at a rolling window of approximately 250 bp in length across the *KDM7A-*DT gene body and data analysis by **(B)** absolute quantification (standard curve of the KDM7A-DT gene body) and **(C)** relative quantification (ΔΔCt method). **(D)** Northern blot analysis using an equimolar mixture of DNA probes NB1 and NB2 against total denatured RNA from MRC5 cells overexpressing full-length KDM7A-DT compared with the vehicle control. Amplification and gel electrophoresis analysis of two KDM7A-DT cDNA amplicons overlapping probes NB1 and NB2 (NB1&NB2) revealed that the full-length transcript accumulates mainly in the nucleus. At the same time, its ncRNA intermediate is found in the cytoplasm of T47D BRCA cells. **(E)** Northern blot analyses of the KDM7A-DT ncRNA intermediate (probe NB1) and its full-length transcript (probe NB2) in BRCA cell lines of triple-negative (basal-like, normal-like), HER2 and luminal subtype variants showed that only in T47D cells (luminal A subtype) was the full-length transcript specifically expressed.

KDM7A-DT gene is a single exon lncRNA located in the antisense direction at the promoter of the protein-coding gene *KDM7A*. To gain insight into the biogenesis of KDM7A-DT in response to OS, 5’ and 3’ RACE analysis (**Figure 2A**) was performed using total RNA isolated from MRC5 treated with OS for 30 mins. Two stress-induced cap-methylated sites were identified downstream of the annotated transcription start site (TSS) of the RefSeq coordinates for the KDM7A-DT locus at chr7:139877061 (GRCh37/hg19). The first stress-induced cap-methylated site (5’ end) of KDM7A-DT is located at genomic position chr7:139877283 (GRCh37/hg19), 224 bp downstream of the TSS above. Interestingly, 247 bp downstream of this location, at position chr7:139877528 (GRCh37/hg19), our 5′ RACE analysis supports an additional cap-methylated site. From the 3′ RACE analysis, four alternative poly(A)-tailored transcripts were identified with a size range from 530 to 1351 bp (median length = 719.5 bp), while the primary poly(A) end was accurately identified at position chr7:139879440 (GRCh37/hg19) corresponding to the predicted end based on the RefSeq database with a total length from the first stress-induced 5’ site of 2158 bp. In contrast, the size of the RefSeq-annotated KDM7A-DT is estimated at 2380 bp. Overall, ten (10) capped and poly(A)-tailored transcripts were detected: eight intermediate-size ncRNAs, with a size range from 285 to 1351 bp (median length = 726.5 bp) and two long ncRNAs (1911 bp and 2158 bp).

We utilized the UCSC table browser to download CAGE data from the RIKEN project, TSSs from the SWITCH project, cCREs (candidate cis-Regulatory Elements) information from ENCODE project and Repeat Elements from Repeat Masker. CAGE data indicate three TSSs located at the minus strand (TSS A-C) from the onset and upstream of the promoter region PLS_A_, corresponding to the TSSs of the KDM7A coding transcript. One of them (TSS B) is also present at the SWITCH track. In the middle of the promoter region PLS_A_, there is a GACE site at the plus strand (TSS D) corresponding to KDM7A-DT. However, it does not overlap with a predicted SWITCH TSS. At a downstream location, inside the extended promoter region (PLS_B)_ and within the gene body of KDM7A-DT, there are three additional TSSs, located near each other in the plus strand (TSS E, F, G), out of which, TSS E is also present at the SWITCH TSS track, indicating that the position of the primary TSS site for KDM7A-DT could vary significantly up to ~250 bps. In agreement with this, the SWITCH project shows that the TSSs for the coding and non-coding genes are located in the middle of the respective regions spanning the CAGE sites of the corresponding strand (blue boxes for KDM7A and red boxes for KDM7A-DT) situated at the start and at the end of the PLS_A_ promoter box.

Next, we characterized the nascent transcription detected at the divergent promoter region of the gene pair. According to the sequencing tracks from MRC5 GRO-seq and K562 GRO-seq, the genomic region spanning the middle of the PLS_A_ until the end of pELS_B_ corresponds to the boundaries of binding sites peaks of transcriptionally active RNAPII depending on the cell type/state. We have already published RNAPII ChIP-seq and RNA-seq tracks of MRC5 cells under OS conditions (1), showing that the accumulation of transcribing RNAPII extends transcription initiation further downstream from the annotated 5’ site and TSS of KDM7A-DT. Notably, the novel and stress-induced 5’ ends from our RACE analysis described above are located at the end of PLS_B_ and next to a proximal enhancer (pELS**_B_**), respectively (**Figure 2A**). The sequencing tracks from K562 PRO-seq show that RNAPII pausing sites accumulate at the end of PLS_B_. At the same time, it also highlights the presence of additional pausing sites, most overlapping the alternative 3’ ends of our RACE analysis and repeat elements. This observation could indicate that these DNA regions might naturally inhibit RNAPII elongation to generate the full-length transcript under physiological conditions. However, a stress-induced accumulation of divergent pausing RNAPII could promote the elongation of antisense and noncoding transcription.

We designed gene walking primers to analyze the predicted RNA biogenesis at the KDM7A-DT locus by qPCR in MRC5 cells overexpressing either pCDH (empty vector) or KDM7A-DT. The biogenesis of KDM7A-DT transcripts was investigated with a gene walking window of ~250 bp. We estimated the corresponding expression levels by both absolute and relative quantification PCR in MRC5 and BJ cells overexpressing full-length KDM7A-DT compared with the empty vector (pCDH) (**Figures 2B, C**). RACE analysis and RNA-seq mapping indicated that the 1–250 and 1000–1250 bp windows showed close-to-zero expression. Interestingly, the 250–1000 bp region showed the highest absolute expression levels. In contrast, the area between 1250 and 2230 bp showed the highest relative expression levels.

We designed two cDNA probes for northern blot analysis to validate the above results using total RNA isolated from MRC5 cells overexpressing the RefSeq annotated KDM7A-DT and control cells expressing only the empty vector (pCDH). Northern blot probe 1 (NB1) was designed to hybridize to a region that overlaps with most of the intermediate-size (truncated) transcripts predicted by 3′ RACE analysis (size range of 500–1000 bp), targeting the area immediately after the secondary 5′ start site. Northern blot probe 2 (NB2) was designed to hybridize at the end of KDM7A-DT, overlapping the gene walking window of 1750–2238 bp with the region amplified by our qPCR primer pair. NB1 hybridization revealed a prominent ~700 bp band (intermediate/truncated ncRNA) abundant in both groups (**Figure 2D**, left panel). NB2 hybridization only occurred in the KDM7A-DT-overexpression group in a particular ~2200 bp band (representing the full-length lncRNA transcript), validating our previous observations (**Figure 2D**, middle panel). To infer whether the identified ncRNAs of intermediate length correspond to autonomous transcripts or byproducts of RNAPII machinery during KDM7A-DT biogenesis, we performed subcellular fractionation of MRC5 cells, followed by northern blot analysis of RNA isolated from nuclear and cytoplasmic fractions, using an equimolar mixture of NB1 and NB2. The band representing the full-length KDM7A-DT RNA showed a weak but specific band at the nucleus with a second band in the cytosol approximately 200–300 bp shorter in length. In sharp contrast, the band corresponding to the intermediate length ncRNA indicated abundant cytosolic localization (**Figure 2D**, right panel).

Northern blot analysis, the gold standard analysis for RNA validation, was then utilized to quantify the transcript levels of full-length KDM7A-DT compared to its ncRNA intermediates in BRCA cell lines available in-house (**Figure 2E**). To characterize the length variability of the *KDM7A-DT* locus and its transcripts expression in BRCA cells *in vitro*, we performed a Northern blot analysis of total RNA in the luminal A (MCF7, and T47D), HER2 (SKBR3) and triple-negative (HsS78T, MDA-MB-231, and MDA-MB-468) cell lines. Interestingly, the intermediate ncRNAs detected by NB1 were expressed abundantly and uniformly across all BRCA cell lines analyzed, indicating a nonspecific but robust pattern of accumulation, as it was also observed in non-malignant cells above In contrast, NB2 signal detection revealed that KDM7A-DT full-length lncRNA shows, like in non-malignant cells, a weak but sharp band with a regulated expression pattern across BRCA cell lines. Importantly, full-length KDM7A-DT showed a robust and abundant expression in the T47D cell line, which was later included in downstream experiments to provide mechanistic insight regarding the BRCA-associated pathways regulated by endogenous KDM7A-DT knockdown.

### KDM7A-DT sequence directionality is necessary for inducing complete phenotype abnormalities in nonmalignant fibroblasts

**Figure 3:**
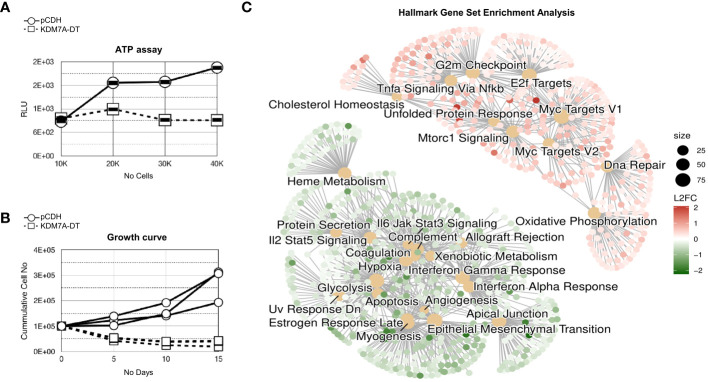
KDM7A-DT significantly inhibited the survival and growth of nonmalignant MRC5 cells by affecting the RNA levels of genes involved in processes associated with stress and stress response pathways. **(A)** KDM7A-DT overexpression significantly decreased the viability of MRC5 cells as measured by the colorimetric ATP assay. **(B)** KDM7A-DT abrogated long-term cell growth as measured by absolute cell numbers over three passages (15 days). **(C)** GSEA results are depicted as a cnetplot (category network) using cancer hallmark gene set terms. GSEA was based on log_2_FC resulting from differential expression analysis (expression quantified using microarrays) in MRC5 cells overexpressing KDM7A-DT or vehicle control (each group was run in triplicate). The top significant gene sets that include upregulated and downregulated genes are represented and colored based on gene log_2_FC. The scale of dot sizes is based on the gene set size.

To determine the functional role of KDM7A-DT in non-malignant cells, we first performed overexpression experiments using human fibroblasts MRC5 as a biological model (Methods). MRC5 cells overexpressing KDM7A-DT showed reduced cell cycle proliferation (**Figure 3A**) and long-term survival (**Figure 3B**). In addition, KDM7A-DT overexpression was associated with a distinct stress-related morphology (enlarged and flattened cells) compared with cells expressing only the vehicle control (pCDH) (**Supplementary Figure S2A**). We stably overexpressed its complementary clone in reverse orientation to confirm that the resulting phenotype of KDM7A-DT overexpression was not merely due to generic toxicity, such as an effect of dsRNA accumulation. MRC5 cells overexpressing reverse-oriented KDM7A-DT exhibited similar cellular morphology, cell cycle progression, and γH2AX levels compared with negative control cells (**Supplementary Figures S2B**, **S2C**). Thus, KDM7A-DT overexpression shows a unique strand-specific functional directionality in inducing the observed stressed cellular phenotype and gene expression pathways.

### KDM7A-DT-mediated global reprogramming of nonmalignant fibroblasts

We conducted microarray-based gene expression profiling to examine the effects of KDM7A-DT overexpression on the transcriptome of nonmalignant fibroblasts. Subsequently, we performed differential expression analysis by comparing a group of four independent biological replicates of MRC5 cells overexpressing KDM7A-DT with a group of samples from control cells (**Supplementary Tables S2A, B**). We found that KDM7A-DT overexpression provides global changes in gene expression profiles. Statistics of the significant differentially expressed genes in MRC5 cells (p<0.05; fold change (FC) >1.2) show 1745 upregulated and 1007 downregulated (coding and non-coding) genes, respectively.

In the list of top-upregulated DEGs (23 transcripts, p< 0.002 and FC > 2) (**Supplementary Table S2D**), c-Myc and nuclear receptors genes were significantly enriched. Genes of ‘regulation of lipid and steroid metabolic processes’ (GO: 0019216, n = 8 genes GO: 0019218, n = 5 genes) and ‘lipid biosynthesis and glycerolipid metabolism’ (STRING-CL: 14136, n = 6) are there. Also, we observed SIN3-HDAC complex associated factor (SINHCAF) transcripts (FAM60/60A) that map to chromosome 12p11 and regulate the expression of the genes that encode components of the TGF-beta signaling pathway. The complex changes cell morphology and increases cell migration. The top list of KDM7A-DT-mediated downregulated DEGs (n = 88, FC< 0.5) was found enriched in gene sets, including extracellular matrix organization, collagen-containing extracellular matrix growth factor binding, platelet-derived growth factor binding, regulation of the developmental process, blood vessel development, and regulation of cell population proliferation.

The results of GSEA against the MSigDB cancer hallmark gene sets showed that due to overexpression of KDM7A-DT in MRC5 cells. We further extended our analysis through Gene Set Enrichment Analysis (GSEA) utilizing MSigDB cancer hallmark gene sets (**Figure 3C**). Among the 31 enriched gene sets, 10 were upregulated, including the notable c-Myc targets V2 (NES = 2.77, GR = 0.64) and c-Myc targets V1 (NES = 2.51, GR = 0.49), and 21 were downregulated, such as the EMT pathways (NES = -2.96). EMT was the most ‘negatively’ enriched gene set and had the third highest gene ratio (GR = 0.48). The *functional connectivity analysis* via STRING revealed a highly interconnected protein-protein interaction network. The 95 MYC V2-associated proteins were particularly interesting, with 83 involved in cell metabolism processes, 57 in nucleobase-containing compound processes, 53 in gene expression regulation, and 10 associated with the DNA damage response (DDR) pathway.

In contrast, the negatively enriched DEG set referring to the KDM7A-DT overexpression was associated with EMT pathways (NES = -2.96), interferon-alpha response (NES = -2.43), coagulation (NES = -2.20), apical junctions (NES = -2.15), interferon-gamma response (NES = -2.06), apoptotic pathway (NES = -1.72) and Estrogen Response Late (NES= -1.57) and Early (NES= -1.48) in MSigDB Hallmark gene set. STRING GO enrichment analysis of the 88 genes provided additional evidence supporting the role of KDM7A-DT in the inhibition of collagen-containing extracellular matrix, extracellular matrix organization, ‘regulation of cell migration’, ‘cell adhesion’, ‘growth factor binding’, MET promotes cell motility gene expression. STRING analysis also detected high enrichment of the identified specific gene sets involved in ‘perivascular fibroblasts that predict poor immunotherapy response’ and ‘tumor-derived fibroblasts and normal tissue-resident fibroblasts revealed fibroblast heterogeneity in breast cancer.’ **Supplementary Tables S2E, S2F** provide detailed characterization of Geneset Enrichment Analysis for GO Terms and Hallmark Geneset pathways in differential expressed genes mediated by the overexpressed KDM7A-DT in MRC5 cells.

### *KDM7A-*DT mediates suppression of EMT Type II gene set in nonmalignant fibroblasts

The EMT pathway is a collection of protein-coding and non-coding genes classified into I, II, and III types (26, 27, 49). To robustly specify our 88-gene EMT signature represented in **Supplementary Table S3**, we used the term ‘fibroblast’ and performed a tissue type enrichment analysis using the EnrichR tool applied to a database of tissue gene sets ARCHS4. We proposed that the total number of genes in the tissue collection should not be over n=88 and be enriched by the EMT gene pathway described in the previous section. Among the 13 largest genesets enriched with the term ‘fibroblast’, the six (6) most diverse genesets – fibroblast (n= 79), foreskin fibroblast (n=69), myofibroblast (n=64), myoblast (n=61) and pericardium (n=60). The adjusted p-value ranged from 6.82E-62 (fibroblast) to 7.33E-35 (pericardium). These 6 GO terms were represented by 84 unique genes of 88 genes that were downregulated after KDM7A-DT amplification and annotated in the MSigDB Hallmark as the EMT geneset. Panther bioinformatics GO enrichment analyses of the 88 geneset (**Supplementary Table S3**) showed significant enrichment of lung fibroblasts. So, these results support our hypothesis that the 88 gene set represents signature EMT Type II. This signature is biologically and molecular highly informative: it includes genes of fibroblast morphology and functions, wound healing, myogenesis, tissue regeneration, immune cells, inflammatory responses, interferons response, tissue remodeling, apical junction, specific production of extracellular matrix components (see more details in **Supplementary Tables S2F, S4**).

Interestingly, it has been shown that a *mesenchymal-epithelial transition* (MET)-like pathway could occur in fibroblasts (49, 50). It has been shown that the brain enriched neuronal miRNA-inducible and striatal-enriched transcription factors can reprogram the fibroblasts from healthy subjects and patients with Huntington’s disease (HD) into induced pluripotent stem cells (iPSCs) and then into striatal medium spiny neurons (MSNs) (GEO data: GSE84013) (50). Our analysis of these datasets provides the list of MET genes altered after neuronal differentiation. We found that 76 and 83 of the 88 Type II gene signature specified in MRC5 cells were downregulated in striatal neurons converted from normal and HD fibroblasts, respectively (**Supplementary Table S3**). These findings suggest that the proliferating lung fibroblasts of our study may be transformed to a less mesenchymal (partial) phenotype due to KDM7A-DT overexpression.

To clarify potential pathobiological components of the KDM7A-DT-depleted EMT pathway–associated gene signature, we used dbEMT-2.0 (51) and EMTome (52) - the comprehensive databases for EMT genes with experimentally verified information about pro-oncogenic and tumor suppressor gene functions. According to dbEMT-2.0, in our 88 KDM7A-DT down-regulating EMT Type II pathway–associated gene signature, only *PDGFRB* is associated with oncogenic role, *WNT5A* is related to dual (tumor suppressor- and oncogenic- like) functions, and seven genes (*IGFBP3*, *CDH11*, *ITGA5*, *LOX*, *CD44*, *ITGB1*, and *FBLN1*) are EMT pathway–associated tumor suppressor genes. The enrichment of tumor suppressor genes in our gene subset was significant (two-sided p = 1.00E-6, by the ratio of two binomial proportion exact tests). These results suggest that KDM7A-DT overexpression in nonmalignant proliferating fibroblasts could activate pro-oncogenic pathways and preferentially reduce the expression of the tumor suppressor genes involved in the EMT II pathway.

### *KDM7A-DT* overexpression identifies genomic alterations determined by gain or amplified events

Multiple genetic and genomic alterations drive tumor heterogeneity and underlie much of the uncertainty when offering precise and personalized therapeutics. Cancer aneuploidies (i.e., whole-chromosome or whole-arm imbalances represented by structural deletions and chromosome arm alterations) are common events affecting BRCA and many other cancer types. Copy number alternations (CNA), comprising genome-wide deletion or amplification of genomic DNA fragments, are also a common characteristic of cancer progression. Aneuploidy and CNA are both stochastic processes underlying cancer progression and markers for prognosis and survival. To identify genetic factors affecting *KDM7A-DT* and its products, we analyzed gene locus and genome-wide factors, referring to KDM7A-DT locus-specific somatic CNA and aneuploidy. We first asked how stable the KDM7A-DT locus is across cancer types. Then, we analyzed the KDM7A-DT associations of comprehensive BRCA genome CNV and transcriptome datasets from TCGA and the PCAWG Consortium (25, 53). There were CNAs and major classes of mutations within KDM7A-DT in 2583 tumor samples representing 46 distinct cancer types. Aneuploidy score (AS) was used to quantify the correlation between KDM7A-DT expression level and aneuploidy (25, 53).

**Figure 4:**
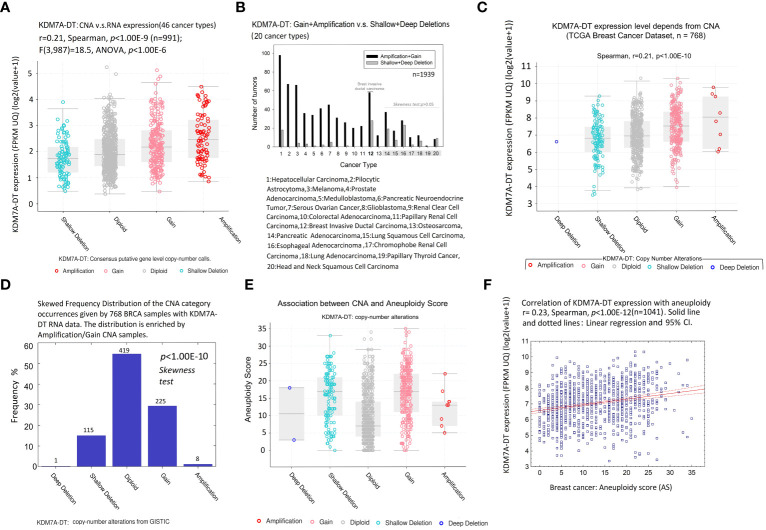
CNA and aneuploidy preferentially modulate KDM7A-DT expression in BRCA. **(A)** Gene expression of KDM7A-DT [log_2_(x+1)] defined in 46 cancer types has a positive linear trend regarding KDM7A-DT CNA score from shallow deletion, diploid, gain, and amplification categories (*p*< 1.00E-06, *n* = 991, ANOVA). Colored circles: patient data; horizontal bars: 99% confidence interval, with the mean value (horizontal line) between the bars. **(B)** Distribution of the number of tumors carrying KDM7A-DT: gain + amplification *versus* shallow + deep deletion in the 20 most representative cancer types. BRCA genome CNA datasets were downloaded from TCGA and the PCAWG Consortium (https://www.cbioportal.org/study/summary?id=pancan_pcawg_2020) (*n* = 1939). **(C)** KDM7A-DT expression as a function of KDM7A-DT CNA. The TCGA level 3 interpreted BRCA dataset was downloaded from cBioPortal (https://www.cbioportal.org/datasets) (*n* = 768). Colored circles: patient data; horizontal bars: 99% confidence interval, with the mean value (horizontal line) between the bars. **(D)** Skewed frequency distribution of the KDM7A-DT CNA category occurrences given by 768 TCGA BRCA samples with KDM7A-DT RNA expression data. The distribution is enriched by the amplification/gain CNA samples. CNA-DT data from GISTIC. **(E)** aneuploidy score (AS) across KDM7A-DT CNA categories. The TCGA BRCA dataset was downloaded from cBioPortal. **(F)** TCGA BRCA KDM7A-DT expression is positively correlated with AS.

Surprisingly, we observed KDM7A-DT amplification in 7.32% (189/2583) cancer cases (**Supplementary Figure S3A**). In contrast, there were deletions in a few patients. Furthermore, we observed that there was a positive linear trend for log_2_ (gene expression signal) of KDM7A-DT in 46 cancer types with the CNA score from shallow deletion, diploid (i.e., average/normal copy number), gain, and amplification categories (*p*< 1.00E-06, ANOVA, **Figure 4A**).

Due to the small number of samples represented by many cancer types in the collected dataset, the CNA distribution of KDM7A-DT was analyzed in 20 selected cancer types, comprising at least 30 samples. We calculated the difference between the sum of sample numbers represented by amplification and gain events and the sum of sample numbers characterized by deep and shallow deletions. Using these numbers, we calculated two-sided *p*-values, testing the null hypothesis that the discrete distribution function (histogram) with three categories – amplification and gain, diploid, and deep and shallow deletions – follows the symmetrical function (Symmetry test, see Methods). Overall, 13 out of 20 tumor types (including 12: Breast Invasive Ductal Carcinoma (BIDC)) showed significant enrichment of the amplification and gain categories (*p*< 0.005; **Figure 4B**; **Supplementary Table S5**). The exact frequency distribution asymmetry trend was observed for the other seven tumor types, but the *p*-value was insignificant (primarily due to the small sample size). We observed that the tumor types with highly significant KDM7A-DT gain and amplification were highly aggressive, including hepatocellular carcinoma (*p* = 8.8E-13), pilocytic astrocytoma (*p* = 3.43E-10), melanoma (*p* = 5.21E-10), prostate adenocarcinoma (*p* = 5.16E-08), medulloblastoma (*p* = 4.20E-07), and colorectal carcinoma (*p* = 6.14E-04). BIDC was part of the enriched in KDM7A-DT gain/amplification per cancer type list (p = 1.22E-03), showing at the same time an increased accumulation of deletions as well.

Using BRCA TCGA data collection, we downloaded a level 3 interpreted dataset CNV and expression profiles from cBioPortal. **Figure 4C** shows that KDM7A-DT expression correlated positively with its corresponding CNA events (*r* = 0.21, *p<* 1.00E-10, Spearman), and the link is defined by the linear regression model (*p<* 1.00E-10, ANOVA). **Figure 4D** shows a significant enrichment of KDM7A-DT expression in gain and amplification *versus* shallow and deep deletion KDM7A-DT CNA categories (*p*< 1.00E-10, symmetry test). Only one sample harbored a deep deletion of KDM7A-DT. We then analyzed the AS, quantifying the whole chromosome or chromosome-arm imbalance level of TCGA BIDC samples (54, 55). Aneuploidy is significantly increased in samples with (i) shallow depletions, (ii) gain, and (iii) amplification of KDM7A-DT compared to its average copy number in the diploid chromosome (**Figure 4E**). However, KDM7A-DT expression correlates positively with AS (*r* = 0.23, *p*< 1.00E-12, Spearman), suggesting an essential role of BIDC AS, in parallel with KDM7A-DT gain and amplification events, in the pro-oncogenic functions of KDM7A-DT expression (**Figure 4F**).

### Association between *KDM7A-DT*’s RNA and CNA levels with corresponding chromosome-level and whole genome instability among BRCA patients’ subtypes

**Figure 5:**
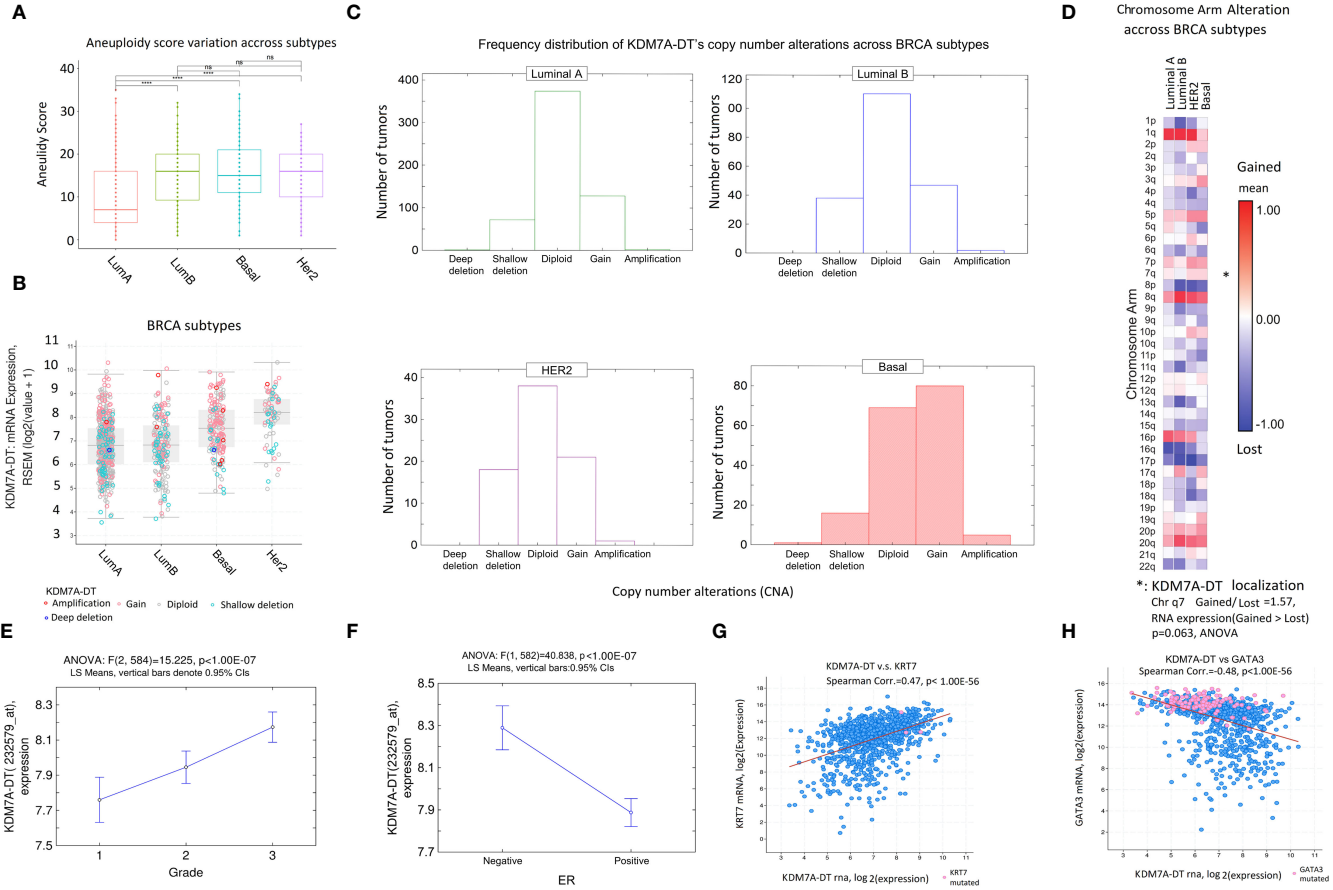
Clinical relevance of KDM7A-DT alterations due to aneuploidy in molecular BRCA subtypes and clinical factors. **(A)** AS is higher in aggressive breast cancer subtypes (luminal B, HER2, and BL) than in the less aggressive luminal A subtype. **(B)** KDM7A-DT expression levels in luminal A and luminal B BRCA subtypes are not associated with the CNA pattern (indicated by the proportion of red dots); in both TCGA BRCA subtypes, the expression is significantly lower than in the HER2 and BL cell subtypes (p< 1.00E-07, Mann–Whitney test). **(C)** Frequency distribution of genome alterations in BRCA subtypes. Only in BL was the KDM7A-DT expression level significantly associated with gain and amplified events (*p*< 0.0001, symmetry test). **(D)** Heatmap of chromosome arm alteration quantification across BRCA subtypes. Categorizing samples with KDM7A-DT expression levels by CNA on chromosome 7q shows that gain and amplification events are enriched compared with shallow deletion events. **(E)** KDM7A-DT expression is positively and linearly associated with a histological grading system. **(F)** KDM7A-DT expression is negatively associated with ER BRCA status. **(G)** Significant positive correlation between KDM7A-DT RNA and *KRT7* mRNA levels (*r* = 0.47, *p*< 1.00E-56, Spearman). **(H)** Significant negative correlation between KDM7A-DT and *GATA3* mRNA levels (*r* = -0.48, *p*< 1.00E-56, Spearman). TCGA RNA-seq and clinical data were used (*n* = 1084). Stars denote significance levels in p-values: One star (*) for p < 0.05, Two stars (**) for p < 0.01, Three stars (***) for p < 0.001, and Four stars (****) for p < 0.0001. “NS” indicates non-significance (p > 0.05).

We examined the AS distribution in PAM50-based BIDC subtypes (luminal A, luminal B, HER2, and BL). **Figure 5A** shows that AS is significantly higher in the luminal B, Basal, and HER2 BRCA subtypes compared to the Luminal A subtype. Indeed, the AS score in the Luminal A subtypes was approximately two times lower, indicating that it might represent the break-even point of aneuploidy level for aggressive BRCA subtypes (Luminal B, Basal, and HER2).

Also, according to TCGA data analysis, KDM7A-DT RNA levels weren’t different between luminal A and B subtypes. However, KDM7A-DT RNA expression levels in Basal and HER2 subtypes are elevated (**Figure 5B**, see also **Supplementary Figure S4A**). To explain these findings, we compared the frequency distribution of the KDM7A-DT CNA categories (**Figure 5C**). We did not find a significant association between CNA and expression levels of KDM7A-DT in luminal A and B subtypes, while in HER2 and Basal cell subtypes, the association was significant (*p*< 1.00E-07, Mann–Whitney test; **Figure 5B**). The expression pattern of KDM7A-DT was analyzed using U133 expression microarrays, and the results showed lower Luminal A and Luminal B expression compared to Basal and HER2 subtypes (**Supplementary Figures S4A–S4D**).

In the Basal subtype, KDM7A-DT’s CNAs were statistically significantly skewed to gain and amplification events (*p*< 0.0001, symmetry test; **Figure 5C**). **Figure 5D** supports these findings by showing the chromosome level alterations in the 7q arm (where KDM7A-DT is localized) using a heatmap analysis to quantify gain versus deletion alterations across BRCA subtypes. In HER2 and Basal subtypes, chromosome 7q showed enrichment of gain and amplification events compared to shallow deletion events. At the same time, even though the AS of the whole genome increases in Luminal B compared to Luminal A subtype by approximately two-fold (**Figure 5A**), within the 7q chromosome arm, CNA gain and amplification events were not significant in Luminal B compared to Luminal A subtypes indicating that KDM7A-DT’s chromosome-level instability is a critical contributor of KDM7A-DT overexpression (**Figure 5D**). Hence, CNA gain/amplification of KDM7A-DT is observed and essential for explaining the KDM7A-DT overexpression in Basal and HER2 BRCA subtypes but not in Luminal A and B subtypes.

### KDM7A-DT levels are elevated in ER-negative aggressive BRCA and correlate with biomarkers of the basal subtype

The Affymetrix probe-set with ID 232579_at maps to the 3′ end of the full-length KDM7A-DT transcribed gene. To analyze KDM7A-DT expression in the biological function, cell subtype, and clinical contexts, we used our collection of six Affymetrix U133A and U133B expression microarray datasets and associated clinical data (see Methods). We used standard batch effect correction, log_2_-normalized expression, and outlier exclusion fitted statistical quality control data criteria. Categorizing the KDM7A-DT expression based on histologic grades suggested that the log2-normalized expression level of the full-length KDM7A-DT transcript is a linear regression function of BRCA histology grades (**Figure 5E**). Accordingly, its expression is elevated in samples with estrogen receptor (ER) negative status (**Figure 5F**). These results were confirmed with independent BRCA datasets (**Supplementary Figures S4B, S4C**). A negative ER status, typical of the Basal and HER2 subtypes, is often associated with BRCA mutations and may exhibit distinct molecular features, like accumulation of oxidative DNA damage, that could contribute to the genomic instability observed in these cancers. Furthermore, we noticed a robust negative correlation between KDM7A-DT expression and *ESR1* mRNA levels and between KDM7A-DT expression and ER protein levels, as detected by specific antibodies (**Supplementary Figures S4G, S4H**).

Using TCGA BRCA RNA expression dataset (*n* = 1084), we observed a highly significant positive correlation between KDM7A-DT and *KRT7* mRNA levels (*r* = 0.47, *p*< 1.00E-56, Spearman; **Figure 5G**) but a highly significant negative correlation between KDM7A-DT and *GATA3* mRNA levels (*r* = -0.48, *p*< 1.00E-56, Spearman; **Figure 5H**). *KRT7* overexpression is a reliable marker of the Basal subtype cells. Low *GATA3* mRNA expression is associated with the Basal subtype and is a marker of poor patient prognosis. High *GATA3* expression is a reliable marker of luminal subtypes. Furthermore, high *GATA3* expression could contribute to reprogramming Basal cells to a less aggressive phenotype and inhibit metastasis (56). The findings suggest that the expression patterns defined by the signature [KRT7(+/-), KDM7A-DT(+/-), GATA3(-/+), ER(-/+), HER2(-)] may contribute significantly to our understanding of basal and luminal cell’s lineages development and EMT/MET reprogramming pathways in BRCA clinically relevant contexts, such as response to chemotherapy and radiotherapy.

### *KDM7A-DT*’s RNA and CNA levels are associated with tumor mutational burden, TP53 missense mutations, and diverse BRCA clinical scores

**Figure 6:**
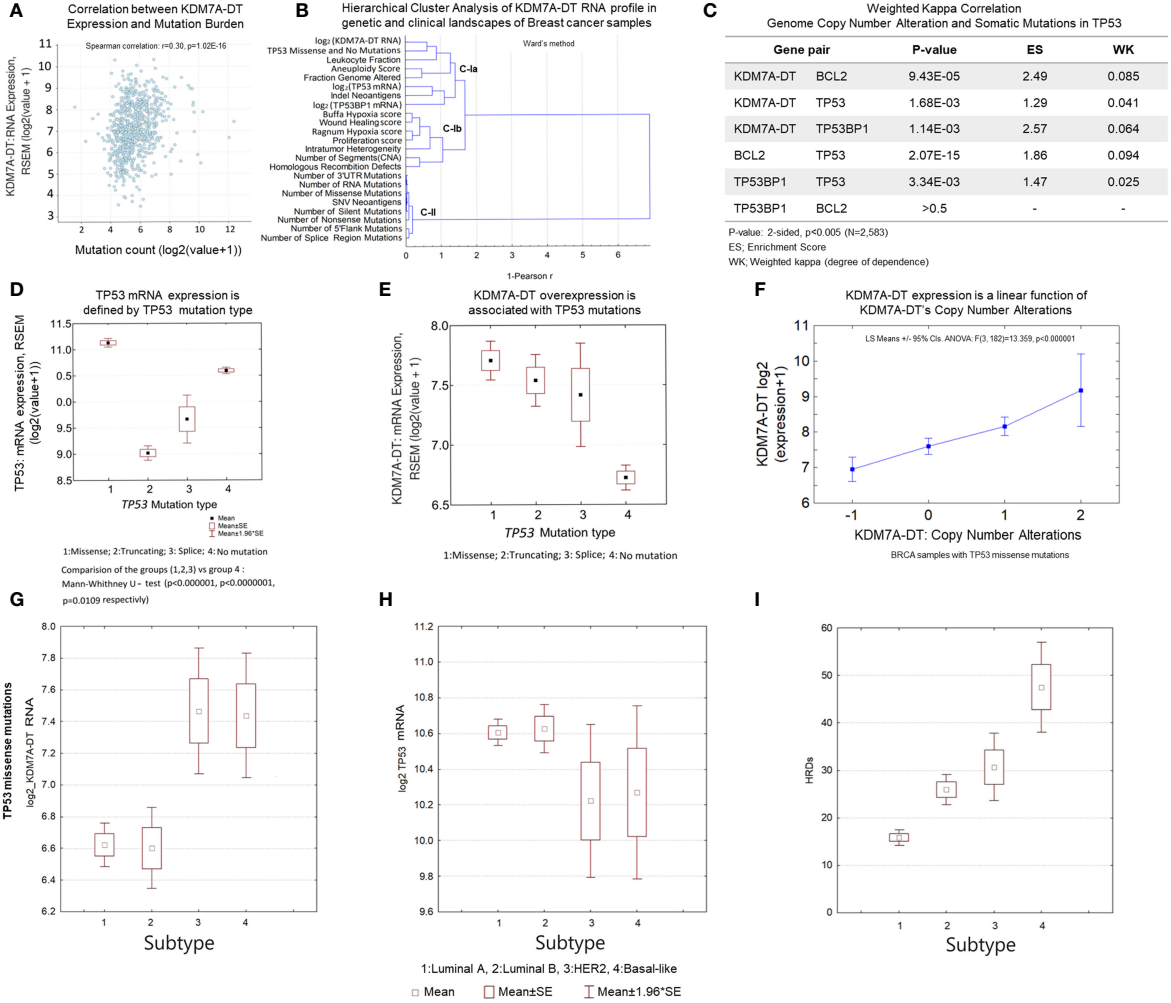
KDM7A-DT is a multifunctional player in genetic variants and genome instability associated with *TP53* missense mutations and DDR pathways in aggressive BRCA. **(A)** *KDM7A-DT* expression is positively correlated with the tumor mutational burden. **(B)** Hierarchical clustering analysis of the KDM7A-DT RNA profile regarding BRCA characteristics, genetic markers, and clinical data perspectives. Sub-clusters C-Ia, C-Ib and cluster C-II are defined using the1-Peason correlation metrics (Ward’s method, Statistica-12). **(C)** Significance and degree of dependence (estimated by weighted kappa correlation) between KDM7A-DT CNA score, *TP53*, *BRCA2*, and *TP53BP1* somatic mutations. **(D)** *TP53* mutation type defines TP53 mRNA expression. **(E)** KDM7A-DT expression level is associated with *TP53* mutation types. **(F)** KDM7A-DT expression is a linear function of KDM7A-DT CNA in TCGA BRCA samples with *TP53* missense mutations. **(G-I)** *KDM7A-DT* and *Tp53* expression, and HRD score data distributing over BRCA subtypes respectively in the case of *TP53* mutation negative patient samples. **(J-L)** *KDM7A-DT* and *Tp53* expression, and HRD score data distributing over BRCA subtypes respectively in the case of *TP53* missense mutation patient samples. Axis X of subtypes’ order on panels **(G-L)** panels reflects an integrative grading system of genome instability, tumorigenic potential, and aggressiveness over the main BRCA subtypes. The TCGA BRCA dataset was downloaded from cBioPortal (Methods, Supporting Materials).

In this section, we studied the different scales (aneuploidy and mutations) referring to genome instability that could lead to high expression of KDM7A-DT and alter the downstream pathways (**Figure 6**). TCGA datasets were analyzed to detect the properties of the KDM7A-DT locus and its expression in BRCA pathogenic processes. Mutation events in PanCancer Atlas and CBioPortal were evaluated for 766 invasive ductal carcinoma cases. Nonparametric correlation tests showed a strong positive association between KDM7A-DT expression and the tumor mutational burden (r=0.30, Spearman, *p* = 1.02E-16, **Figure 6A**). Fraction genome altered (FGA) is strongly associated with mutation count (*r* = 0.42, *p* = 3.35E-44, Spearman).

We carried out paired correlation and unsupervised hierarchical cluster analyses of the log_2_-transformed KDM7A-DT RNA expression level profiles with a group of significant clinical, genomic, and molecular tumor markers, including FGA. **Table 1** shows a summary of statistics for 23 selected TCGA BRCA clinical variables, characterizing tumor gene expression, gene variation, somatic mutations, TP53 mutations, genome alterations, and a group of clinically significant scores correlated with KDM7A-DT’s log2 RNA levels from 1072 tumors of female patients. In particular, these data show that KDM7A-DT expression positively correlates with FGA. Homologous recombination deficiency (HRD) is a phenotype characterized by a cell’s inability to repair double-strand DNA breaks using the HR repair pathway effectively. TCGA PanCancer data prove that tumors with HRD and multiple oncogenic mutations accumulate genomic alterations that may serve as neoantigens and increase sensitivity to immune checkpoint inhibitors.

**Table 1:** Basic statistics of TCGA BRCA samples, clinical variables, and Spearman correlation coefficients between log2 (KDM7D-TD RNA), aneuploidy score, and other reported variables.

Variables	Mean	Std. Dev.	Spearman Corr. Coefficient Log2 (KDM7A-DT RNA)	Spearman Corr. Coeff. Aneuploidy Score
**log2 (KDM7A-DT RNA)**	7.63	1.057	1.00	0.24
log2 (TP53 mRNA)*	10.87	0.662	-	-
**log2 (TP53BP1 mRNA)**	10.02	0.630	-0.34	-0.21
**Buffa Hypoxia Score**	5.8	20.32	0.36	0.39
**Ragnum Hypoxia Score**	9.5	9.95	0.26	0.38
**3’UTR Mutations**	30.8	71.09	0.21	0.13
5’Flank Mutations*	1.97	2.752	-	-
**Missense Mutations**	274	700.7	0.30	0.27
**Nonsense Mutations**	29.4	103.02	0.21	0.10
**RNA Mutations**	8.9	20.1	0.16	-
**Silent Mutations**	95.4	224.79	0.26	0.23
Splice Region mutations*	6.97	17.005	-	-
**Aneuploidy Score**	14.8	6.76	0.24	1.00
**Fraction Genome Altered**	0.41	0.186	0.14	0.65
**SNV Neoantigens**	139	330.8	0.31	0.28
**Indel Neoantigens**	98	225.7	0.14	-
**Leukocyte Fraction**	0.22	0.124	0.24	-
**Intratumor Heterogeneity**	0.15	0.117	0.19	0.42
**Proliferation Score**	0.42	0.479	0.17	0.38
**Wound Healing Score**	0.12	0.151	0.23	0.35
**Number of Segments (CNA)**	7.77	0.982	0.14	0.41
**Homologous Recombination Defects**	37.5	22.07	0.23	0.43
TP53 Missense and No Mutations**	1.49	0.504	0.37	0.29

*Marked correlations are not significant at p<0.001.

**TP53 Missense and No Mutations: binary score vector(1,2): 1: TP53 missense mutation, 2: TP53 wild type.

To evaluate the associations of the alterations of *KDM7A-DT* expression with immune response and tumor neoantigens, we included in our analysis the leucocyte fraction (LF), single nucleotide variant (SNV) neoantigen, and indel neoantigen (IN) counts. Collectively, we considered our mutation score (MS) – a binary variable that assigns 1 to the wild-type and 2 to mutation events, the log_2_-transformed mRNA levels of *TP53* and *TP53BP1*, the AS, the FGA, HRD, LF, SNV neoantigens, IN counts, the hypoxia score (HS), the proliferation score (PS), the wound healing score (WHS), and counts of several mutation types and genes reported to be altered in TCGA datasets and other publications (25, 56–58). The Spearman correlation matrix (**Supplementary Table S7**) shows that the KDM7A-DT log_2_ mRNA levels correlated significantly positively with 20 of the 23 parameters, including TP53 missense type mutation score, negatively with the mRNA levels of TP53BP1 and not significantly with TP53 mRNA and its splice region mutations. Notably, *TP53* log_2_ mRNA expression correlates significantly (positively) only with the number of *TP53* missense mutations. These results suggest a positive correlation of KDM7A-DT expression with whole-genome alterations and several other scores representing mutagenesis, genome instability, and clinically relevant scores.

In addition, KDM7A-DT CNA levels (diploid/gain/amplification) show a significant association with the number of somatic mutations within genes related to DDR, apoptosis, and P53 pathways (**Figure 6C**), which suggests a role of the KDM7A-DT gain/amplification in these pathways. Our results indicate that KDM7A-DT genetic alterations and expression aberrations in primary BRCA play essential roles in multiple pathways of genome instability, driving cancer progression and the immune response to tumor neoantigens. These findings connect various pieces of evidence, showing that KDM7A-DT CNA and RNA levels are linked in *TP53*-driven missense mutagenesis, DDR/R, wound healing pathways, mutational burden aneuploidy, and genome instability.

### KDM7A-DT expression in BRCA subtypes: TP53 missense mutations role

The role of KDM7A-DT’s expression alterations in BRCA subtypes is poorly understood. Our findings suggest that KDM7A-DT expression can be altered by some specific driver mutation(s) in susceptible tumor gene(s), including *TP53*. According to data, *TP53-*mutation mRNA could be overexpressed compared with the wild-type samples, and missense mutations may be driver mutations in BRCA. **Supplementary Table S6** shows TCGA BRCA samples with missense mutations (which represent 51% of all observed mutation events (*n* = 666930) and their distribution across all BRCA subtypes.

We observed that TP53 mRNA levels differentiated across mutation types: 1: missense, 2: truncating, 3: splicing, and 4: no mutations (control) (**Figure 6D**). According to **Figure 6D**, the expression of the TP53 mRNA is highest in samples with missense mutations compared to those with different types of mutations, and control samples (no mutation) showed the second highest expression levels. Interestingly, there is no difference in TP53 mRNA levels across BRCA subtypes when the samples with studied TP53 mutation types are not separated (**Supplementary Figure S3B**). When the mutation types are categorized, however, it is evident that the distinct frequency distributions are mixed and that TP53 mRNA overexpression is significantly associated with HER2 and Basal (**Supplementary Figure S3C**). **Supplementary Table S10B** shows the frequency distributions of the number T53 missense mutation event and the TP53 negative mutation event over the BRCA subtypes. The frequency distributions are not similar. *TP53* mutations occur in 5.3% of normal-like, 14.7% of luminal A, 24.7% of luminal B, 20.0% of HER2, and 35.5% of basal BRCA subtypes, while *TP53* mutation-negative events occur in 4.5% of normal-like, 61.9% of luminal A, 23.1% of luminal B, 4.3% of HER2, and 6.3% of basal BRCA subtypes. Luminal A, HER2, and Basal subtypes show the most contrast differences between the distributions.

We observed significant *KDM7A-DT* overexpression in each TP53 mutation type *versus* no mutation samples (**Figure 6E**). However, the highest KDM7A-DT expression levels occurred in the samples with *TP53* missense mutations. Next, gene expression data analysis showed in the samples with missense *TP53* mutations (where *TP53* mRNA is over-expressed), KDM7A-DT RNA levels are associated with KDM7A-DT gain and amplification mutations (*p*< 1.0E-06, ANOVA, **Figure 6F**; **Supplementary Figure S3D**). In contrast, the conditional correlation between KDM7A-DT RNA levels and other TP53 mutations (i.e., truncating mutations **Supplementary Figure S3E**) was insignificant. Next, we identified the subsets of BRCA samples with TP53 missense (essential) mutations and carried out an analysis of statistical properties of specified BRCA subtypes (1:Luminal A; 2:Luminal B, 3:HER2; 4:Basal) (**Figures 6G–I**). **Figure 6G** shows the differential expression pattern of KDM7A-DT (log_2_ expression) in BRCA where, in the cases of TP53 missense mutations, there is an expected significant increase in the ER- subtypes, Basal and HER2 v.s. other subtypes. In addition, *TP53* expression levels in HER2 and Basal subtypes are significantly reduced versus luminal A/B (**Figure 6H**). These results prove that within the TP53 missense mutations context, the differential expression patterns of KDM7A-DT and TP53 are dichotomizing, essentially being a mirror-image of each other, luminal A/B subtypes v.s. Basal and HER2 subtypes. **Figure 6I** shows that HRD scores demonstrated different levels across all subtypes, with Basal and HER2 subtypes showing higher HRD scores than luminal subtypes. Luminal B, as expected (based on their corresponding AS score), showed significantly higher HRD scores than luminal A. However, HER2 subtypes showed notable lower levels of HRD scores compared to Basal cases, suggesting that in terms of HRD levels, luminal B and HER2 subtypes show a similar baseline compared to Luminal A (significantly lower) and Basal (significantly higher) subtypes.

The results provide evidence that TP53 missense mutation events play a causal role in KDM7A-DT gene amplification and aberrant expression and also suggest that KDM7A-DT functions in luminal A/B, Basal, and HER2 BRCA subtypes can be involved in various stress-dependent pathways. In association with HRD scores and TP53 mRNA levels, they provide a distinct ranking of BRCA subtypes (luminal A, luminal B, HER2, Basal) that indicate KDM7A-DT’s pathogenic roles in BRCA subtype contexts (59).

### KDM7A-DT-defined concordantly expressed mRNAs and proteins are involved in oncogenic PPI networks, including driver BRCA genes showing subtype-specific mutation events

It is essential to clarify the integrative roles of KDM7A-DT expression in PPI networks. We carried out KDM7A-DT expression functional associations using the mRNA and protein expression datasets available in the PanCancer BRCA dataset (25, 57). TCGA collection includes 191 proteins whose expression was measured by RPPA in 876 TCGA BRCA samples (see Methods). Out of the 191 protein symbols, 160 are unique. We performed GO and network analyses using the STRING-11 software. The list of 160 proteins was highly enriched in numerous GO terms involved in ‘cellular response to stimulus’ (GO:0051716) and ‘cellular response to stress’ (GO:006950) and pathways related to apoptosis, proliferation, BRCA, and other cancers.

We observed that the expression of 46% (74/160) of proteins was significantly correlated with the KDM7A-DT mRNA level (*p*< 0.05, Spearman correlation): 44 positive and 30 negative correlations. We selected the significantly correlated mRNAs (*p*< 0.001, Spearman). Then, we selected both mRNA and proteins encoded by the same gene. We selected 46 protein-mRNA pairs that showed the same correlation directionality with KDM7A-DT: 24 protein–mRNA pairs were positively correlated, and 22 protein–mRNA pairs were negatively correlated with KDM7A-DT (**Table 2**). The details of the protein annotation are presented in **Supplementary Table S8**. Using the ingenuity pathway analysis (IPA) functional enrichment tool, we observed that 45 of the 46 selected mRNAs/proteins were categorized by the term ‘Apoptosis’ in the ‘Cell death and survival category. The gene subsets under the terms ‘Growth of tumor’ (*n* = 33), ‘Metastasis’ (*n* = 34), and ‘Cell cycle progression’ (*n* = 29) were also highly enriched. In particular,’Cancer, organismal injury and abnormalities’ is represented by the term ‘Invasive tumor’ (*n* = 36, *p* = 7.3E-30). We defined a gene’s invasive tumor property as ‘increased’, ‘affected’, and ‘decreased’ (**Table 2**). We ranked the order of these properties by our *invasive tumor index* (ITI) 1 (increased), 2 (affected), and 3 (decreased) for our analysis. We identified 15 ‘increased’, 16 ‘affected’ and 5 ‘decreased’ genes that showed invasive tumor properties. All but one of the genes that were significantly positively correlated with KDM7A-DT belonged to the ITI category 1 (N=9; *ANXA1*, AKT3, *EGFR*, *KIT*, *TGM2*, *WWTR1*, *YAP1*, *NOTCH1*, and *SRC*), and 2 (affected, N=7). *BAX* was the only gene with ‘decreased’ invasive tumor properties but positively correlated with KDM7A-DT. In contrast the genes that were significantly negatively correlated to KDM7A-DT expression exhibited an even mixture of all ITI categories, with four (N=4) belonging to the ITI category 3 ‘decreased’ invasive tumor properties (*TP53BP1*, *BCL2L11*, *SMAD3*, and *ARID1A*), six (N=6) to the ITI category 1 (increased) and nine (N=9) to category 2 (affected). The distribution symmetry test showed that the number of genes with ‘increased’ invasive tumor properties was overrepresented in our data compared to those with ‘decreased’ invasive tumor properties (*p* = 0.05). The Kendall tau correlation analysis showed a significant positive correlation between the KDM7A-DT-associated Spearman’s correlation coefficient values of the genes and ITI values (Kendall’s tau = 0.33, *p*< 0.01).

**Table 2:** KDM7A-DT-defined concordant correlated proteins and mRNAs encoded by primary BRCA 46 genes (p<0.015 for RNA and p<0.05 for proteins).

n/n	KDM7A-DT epression: Correlation direction: pos. = 1, neg. = 2	mRNA Symbol	Cytoband	Spearman’s Correlation	p-value	Protein Symbol	Spearman’s Correlation	p-value	Invasive tumors (IPA)	Number of references (IPA)	EMT pathways
A. Positive correlations between KDM7A-DT expression and gene and protein expressions											
1	1	ANXA1	9q21.13	0.267	3.03E-14	ANXA1	0.231	5.21E-12	Increases	35	
2	1	CDH3	16q22.1	0.322	3.16E-20	CDH3	0.228	9.74E-12			
3	1	AKT3	1q43-q44	0.158	8.92E-06	AKT3	0.218	1.60E-10	Increases	36	1
4	1	EGFR	7p11.2	0.286	3.63E-16	EGFR	0.213	1.98E-10	Increases	26	1
5	1	LCK	1p35.2	0.244	4.68E-12	LCK	0.178	1.18E-07	Affects	20	
6	1	KIT	4q12	0.187	1.41E-07	KIT	0.153	5.62E-06	Increases	21	
7	1	TGM2	20q11.23	0.221	4.65E-10	TGM2	0.151	7.19E-06	Increases	6	1
8	1	WWTR1	3q25.1	0.236	2.64E-11	WWTR1	0.131	1.08E-04	Increases	4	1
9	1	G6PD	Xq28	0.242	6.82E-12	G6PD	0.128	1.53E-04			
10	1	CCNE1	19q12	0.249	1.91E-12	CCNE1	0.124	2.35E-04	Affects	29	
11	1	STMN1	1p36.11	0.185	1.87E-07	STMN1	0.121	3.35E-04	Affects	7	
12	1	YAP1	11q22.1	0.152	2.14E-05	YAP1	0.112	8.63E-04	Increases	2	1
13	1	NOTCH1	9q34.3	0.259	1.82E-13	NOTCH1	0.111	9.87E-04	Increases	18	1
14	1	SRC	20q11.23	0.159	8.67E-06	SRC	0.101	2.85E-03	Increases	8	
15	1	TIGAR	12p13.32	0.142	7.02E-05	TIGAR	0.091	6.82E-03			
16	1	ACVRL1	12q13.13	0.152	1.99E-05	ACVRL1	0.091	7.35E-03			
17	1	PRKCA	17q24.2	0.168	2.34E-06	PRKCA	0.086	1.07E-02	Affects	15	
18	1	CHEK1	11q24.2	0.208	4.89E-09	CHEK1	0.086	1.13E-02	Affects	27	
19	1	TFRC	3q29	0.251	1.13E-12	TFRC	0.085	1.20E-02			
20	1	PRDX1	1p34.1	0.213	1.78E-09	PRDX1	0.080	1.75E-02			
21	1	BAX	19q13.33	0.176	8.08E-07	BAX	0.080	1.75E-02	Decreases	32	
22	1	ETS1	11q24.3	0.176	7.92E-07	ETS1	0.078	2.15E-02			1
23	1	YWHAZ	8q22.3	0.147	3.84E-05	YWHAZ	0.074	2.84E-02	Affects	1	
24	1	RAD51	15q15.1	0.186	1.78E-07	RAD51	0.072	3.39E-02	Affects	13	
B. Negative correlations between KDM7A-DT expression and gene and protein expressions											
25	2	GATA3	10p14	-0.474	5.19E-45	GATA3	-0.435	1.03E-41	Affects/Decreases	4*	
26	2	BCL2	18q21.33	-0.500	1.64E-50	BCL2	-0.419	1.60E-38	Affects	31	
27	2	ESR1	6q25.1-q25.2	-0.393	3.24E-30	ESR1	-0.352	5.94E-27	Affects	24	
28	2	PREX1	20q13.13	-0.271	1.50E-14	PREX1	-0.265	1.62E-15	Affects	16	
29	2	TP53BP1	15q15.3	-0.333	1.16E-21	TP53BP1	-0.259	8.14E-15	Decreases	5	
30	2	INPP4B	4q31.21	-0.232	5.75E-11	INPP4B	-0.246	1.62E-13			
31	2	XRCC1	19q13.31	-0.149	2.93E-05	XRCC1	-0.210	3.60E-10	Affects	3	
32	2	RPTOR	17q25.3	-0.185	1.93E-07	RPTOR	-0.203	1.32E-09	Increases	10	
33	2	BCL2L11	2q13	-0.131	2.39E-04	BCL2L11	-0.204	1.25E-09	Decreases	30	
34	2	IRS1	2q36.3	-0.323	2.40E-20	IRS1	-0.179	1.02E-07	Increases	22	
35	2	MAPK9	5q35.3	-0.197	3.08E-08	MAPK9	-0.173	2.66E-07	Affects	19	
36	2	AR	Xq12	-0.163	4.85E-06	AR	-0.151	7.32E-06	Affects	34	
37	2	RPS6KB1	17q23.1	-0.191	8.03E-08	RPS6KB1	-0.149	9.30E-06	Increases	11	
38	2	PGR	11q22.1	-0.190	9.34E-08	PGR	-0.139	3.84E-05	Affects	17	
39	2	RICTOR	5p13.1	-0.210	3.30E-09	RICTOR	-0.131	9.89E-05	Increases	12	
40	2	SMAD3	15q22.33	-0.234	3.54E-11	SMAD3	-0.128	1.46E-04	Decreases	9	1
41	2	CDKN1B	12p13.1	-0.254	5.82E-13	CDKN1B	-0.125	2.03E-04	Increases	28	
42	2	ARID1A	1p36.11	-0.167	2.84E-06	ARID1A	-0.135	2.42E-04	Decreases	33	
43	2	ACACA	17q12	-0.130	2.76E-04	ACACA	-0.124	2.42E-04			
44	2	RAD50	5q31.1	-0.302	5.73E-18	RAD50	-0.118	4.48E-04	Affects	14	
45	2	ERBB3	12q13.2	-0.216	1.16E-09	ERBB3	-0.110	1.16E-03	Increases	25	
46	2	XRCC5	2q35	-0.087	1.50E-02	Ku80	-0.109	1.27E-03			

* References by manual curation for breast cancer (see **Supplementary Materials References**).

The 876 tumor samples with 191 protein data (RPPA) were used to calculate the correlation coefficients (60–63).

Functional enrichment analysis of the 46 KDM7A-DT-defined co-expressed proteins provided a highly enriched gene subset. These proteins create high-confidence physical and functional PPI networks, which we constructed using STRING. We also specified physical PPI and functional networks that allowed us to analyze the data. **Supplementary Figures S5A, S5B** show the significantly differentially enriched PPI networks and subnetworks where KDM7A-DT expression in tumor samples is negatively or positively correlated correspondingly with the mRNA and protein expression levels encoded by the same gene (**Table 2**). In addition, we identified the GO terms (and pathways) that were statistically unique or differentially enriched by comparing only the protein subsets positively and negatively correlated with KDM7A-DT. Among the KDM7A-DT negatively correlated proteins forming physical PPI network modules, we found the following highly enriched terms: ‘positive regulation of cellular metabolic process’ (GO: 0031325), ‘response to hormone’ (GO:0009725), ‘TOR signaling’ (GO:0031929),’ response to lipid’ (GO:0033993), ‘tube morphogenesis’ (GO:0035239), and ‘cellular response to DNA damage stimulus’ (GO: 006974). Among the proteins positively correlated with KDM7A-DT expression forming a physical PPI network, we observed highly enriched subsets for ‘response to stress’ (GO:0006950), ‘wound healing’ (GO:0042060), ‘response to hypoxia’ (GO: 0001666), ‘gene transcription pathway/Pol-II transcription’ (HAS-212436), and ‘transcriptional regulation by p53’ (HAS-3700989). Functional network models generated by STRING provide additional interconnections of the proteins and modules and (as expected) some new enriched GO terms (**Supplementary Figures S5C, S5D**). For example, in the case of the proteins positively correlated with KDM7A-DT, we observed gene subsets defined by the terms ‘positive regulation of cell population proliferation’ (GO:0008284), ‘regulation of programmed cell death’ (GO:0043067), ‘cell developmental process’ (GO:0048869), ‘regulation of phosphorylation’ (GO: 0042225), ‘central carbon metabolism in cancer’ (hsa0530), and ‘proto-oncogene’ (KW-0656).

Physical PPI network analysis of the proteins positively and negatively correlated with KDM7A-DT shows an extensive PPI network (**Supplementary Figure S5E**) integrating the proteins into a comprehensive physical network with well-specified functional modules. The PPI functional enrichment *p*-value for each network is highly significant (*p*< 10E-16), suggesting that the network has significantly more interactions than expected by chance. The combined network enhances the significance of the GO terms and their family members, some of which are observed in **Supplementary Figures S5A–D**. Significant enrichment was also observed in the case of the combined functional networks (**Supplementary Figure S5F**). **Supplementary Table S9** lists the genes/proteins in the enriched GO categories in **Supplementary Figures S5E, S5F**.

Among the list of the top 46 proteins, we observed negatively correlated proteins, such as GATA3 (Rho = -0.44, *p* = 4.98E-41), BCL2 (Rho = -0.42, *p* = 2.10E-38), ESR1 (Rho = -0.35, *p* = 9.70E-27), and TP53BP1 (Rho = -0.26, *p* = 1.27E-15). The expression of these proteins and KDM7A-DT RNA could be affected by common mutation events, such as *TP53* and *GATA3* mutations (**Figures 5H**, **6C**; **Supplementary Figure S6A**). A contingency table of the number of tumor samples with *TP53* and *GATA3* mutations and the tumors without mutations in these two genes distributed across BRCA subtypes is presented in **Supplementary Figures S6A-C**. The percentage of *TP53* mutations is significantly higher in the Basal and HER2 subtypes than in the luminal A and luminal B subtypes. In contrast, the rate of *GATA3* mutations is substantially higher in the luminal subtype than in the BL and HER2 subtypes (**Supplementary Figure S6C**). The top proteins whose expression levels were positively correlated the most with KDM7A-DT RNA expression included ANXA1 (Rho = 0.23, *p* = 5.21E-12), CDH3 (Rho = 0.228, *p* = 9.74E-12), AKT3 (Rho = 0.22, *p* = 1.60E-10), LCK (Rho = 0.178, *p* = 1.18E-07), EGFR (Rho = 0.21, *p* = 1.98E-10), and KIT (Rho = 0.5, *p* = 5.62E-6).

These results suggest that KDM7A-DT is involved in our PPI networks that are related to DDR, apoptosis, tumor aggressiveness, and invasiveness, as well as essential BRCA oncogenes that show subtype-specific mutational events (GATA3:Luminal v.s. TP53:Basal/HER2). Interestingly, among the highly enriched functional categories defined by IPA DB, we observed genes/proteins of ‘cytokine signaling in the immune system’ (HAS-1280215), ‘cell death of immune cells’, and ‘immune cell trafficking’.

### KDM7A-DT expression reveals differential and subtype-specific disease prognoses within BRCA patients

**Figure 7:**
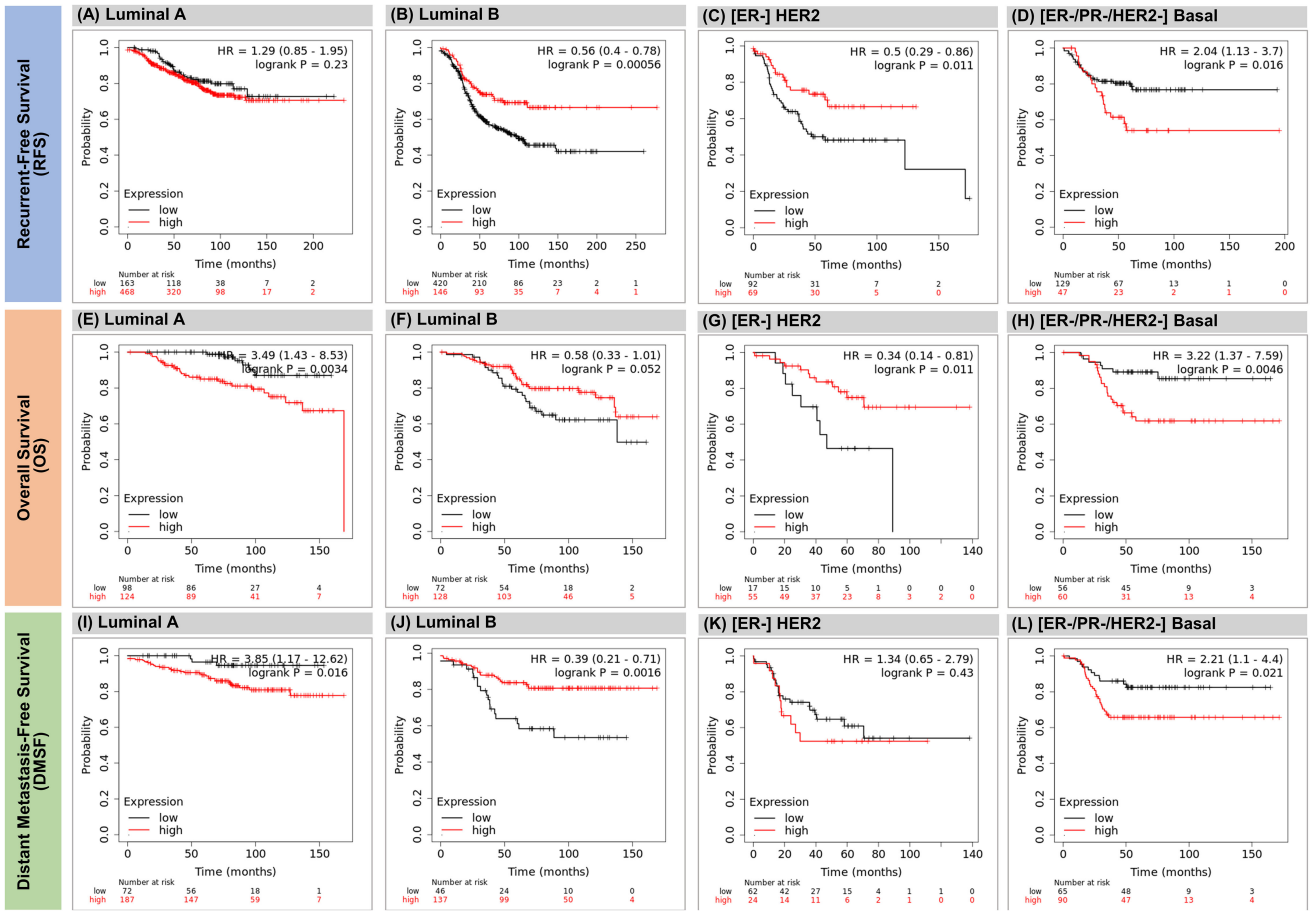
The expression of KDM7A-DT predicts survival differences within the BRCA subtypes. K-M analysis was performed using the KM plotter and its associated meta-cohort database for breast cancer mRNA expression. Patients were split by KM plotter auto-selection method for the best cutoff value (threshold) separating survival data onto two risk groups. In luminal A data, higher expression of KDM7A-DT shows poorly prognostic role defined by OS and DMFS data, but the KDM7A-DT expression is not significant for RFS **(A, E, I)** In luminal B data, higher expression of KDM7A-DT shows favorable prognostic role defined by PFS, OS (near border-line) and DMFS data **(B, F, J)** In HER2+/ER- data, higher expression of KDM7A-DT shows favorable prognostic role defined by PFS and OS, however and its expression is not significant by DMFS data **(C, G, K)** In Triple-negative/Basal subtype patients the higher expression of KDM7A-DT shows poorly prognostic role defined by all survival time data **(D, H, L)**.

We performed a survival prediction analysis based on KDM7A-DT expression as a prognostic factor for BRCA patients (58, 64, 65) (Methods). We propose that KDM7A-DT expression alterations provide subtype-specific identification of relatively lower and higher survival risk groups. Employing the KM-plotter on breast cancer datasets (see Methods, **Supplementary Information**), we calculate ‘the best’ (optimized) cut-off value for the KDM7A-DT expression levels to discriminate the relatively low- and high-risk outcome groups. We calculated the K–M survival functions for recurrent-free survival (RFS), overall survival (OS), and distant metastasis-free survival (DMFS) time in BRCA patient cohorts. For a given survival time model, the risk groups were discriminated via the minimization of log-rank statistics p-values, which defined the cut-off value in the data quartile interval. **Figure 7** shows the predicted time survival probabilities discriminating relatively lower- and higher-risk groups within each BRCA subtype in the above three types of survival time. When for a given risk group, KDM7A-DT expression is higher than the predicted cut-off value and the patients are assigned to the higher risk subgroup, then a role of KDM7A-DT is interpreted as ‘pro-oncogenic’, in the opposite case - as ‘tumor suppressor-like’ factor. The pro-oncogenic and tumor-suppression-like patterns were identified separately for luminal A, B, HER2^+/^ER^-^ and (ER^-^/PR^-^/HER2^-^) Basal BRCA datasets.

In luminal A data, higher expression of KDM7A-DT shows a prognostic role for poor survival as defined by OS and DMFS data. Still, the KDM7A-DT expression does not significantly separate risk subgroups for RFS (**Figures 7A, E, I**). In luminal B data, higher expression of KDM7A-DT shows a favorable prognostic role defined by RFS, OS (near border-line), and DMFS data (**Figures 7B, F, J**). In HER2^+^/ER^-^ data, higher expression of KDM7A-DT shows a favorable prognostic role defined by RFS and OS; however, its expression is not significant by DMFS data (**Figures 7C, G, K**). In contrast, in triple-negative and Basal subtype patients, the higher expression of KDM7A-DT is a robust prognostic risk factor for poor survival defined by all survival time data (**Figures 7D, H, L**).

Thus, according to our results, the KDM7A-DT overexpression in BRCA patients is associated with favorable outcomes within luminal B (RFS, OS, and DMFS) and HER2+/ER- (RFS and OS) subtypes and should be considered as a tumor suppressor like factor. In contrast, the KDM7A-DT overexpression isolates patient groups with poor outcomes, and its function is deemed pro-oncogenic to the Basal (RFS, OS, and DMFS) and luminal A (OS, DMFS) subtypes.

Interestingly, in luminal A, KDM7A-DT alteration is not a significant factor in survival, as defined by the RFS time. This fact aligns with the pathobiology of luminal A tumors, which are mostly highly differentiated, highly sensitive to hormone therapy, low invasive, and have a low proliferative signature expression level. We can see a relatively higher 5-year survival probability (>70%) of RFS for luminal A patients versus the pooled RFS data in each other subtype (**Figure 7A**).

### KDM7A-DT overexpression activates transcriptional signaling of both cell cycle arrest and metabolic pathways while it suppresses inflammatory pathways of T47D BRCA cells

We showed that elevated *KDM7A-DT* transcription triggers specific downstream pathways in nonmalignant cells at physiological and oxidative stress conditions. Our analyses of comprehensive BRCA data suggested that KDM7A-DT genetic alterations and expression aberrations in primary BRCA play essential roles in multiple pathways referring to TP53 missense mutation, CNA, tumor genome instability, uncontrolled proliferation, pathways driving cancer invasiveness, and clinical outcomes. As shown above, the KDM7A-DT locus generates a full-length transcript and several ncRNA intermediates with distinct expression and sub-cellular localization patterns. However, only the full-length transcript was analyzed since only these data are available from the BRCA patient dataset. We aimed to identify the functional role of KDM7A-DT’s intermediates and full-length endogenous transcription in BRCA cells using *in vitro* models.

To characterize the *KDM7A-DT* locus and its transcripts expression in BRCA cells *in vitro*, we used a panel of six cell lines: the luminal A (MCF-7, and T47D), HER2 (SKBR3) and triple-negative (HsS78T, MDA-MB-231, and MDA-MB-468). Except for MCF-7, the other cell lines have a mutation within the *TP53* gene (Cell cultures; Methods). The Northern blot analyses presented in **Figure 2E** depict the expression of the full-length KDM7A-DT transcript among basal, HER2, and luminal cell lines, with notable abundance and stability in the T47D cell line. We chose the T47D cell line as a biological model to study KDM7A-DT’s product(s) functions in BRCA cells. The T47D breast cancer cell line was originally isolated from the metastatic pleural effusion of a 54-year-old female patient. The T47D genome contains a single copy of the *p53* missense mutation at residue 194 (within the zinc-binding domain, L2), which is required for the survival of T47D BRCA cells and makes T47D cells. These cells may be considered a model of a metastatic variant of luminal A subtype controlled by an ER+/PR+/HER- status and the specific pathways defined by original cells (see Discussion).

To identify the variability and functions of endogenous *KDM7A-DT* expression, we carried out antisense oligonucleotides (ASOs) targeting four individual regions of KDM7A-DT based on its newly validated start site. The following are the results of our experiments. We observed that *KDM7A-DT* expression was initially effectively knocked down by chemically modified DNA ASOs targeting individual regions of the KDM7A-DT’s intermediate-sized transcripts (**Figure 2A**; **Supplementary Figure S7A**). KDM7A-DT knockdown by three such ASOs in T47D cells abrogated cell growth compared with control cells transfected independently with a negative ASO (**Supplementary Figure S7B**). We found that knock-downed KDM7A-DT’s start region in T47D cells generated a significant gain of function in DDR/R signaling activation, as indicated by the elevated γH2AX levels, compared with control cells (**Supplementary Figure S7C**). We then sought to reproduce these results by independently targeting the end region of KDM7A-DT and selecting a silencing approach based on LNA gapmers. We designed eight independent LNA gapmers, four targeting an area at the gene’s start (intermediate-sized isoform; IS1–4) (**Supplementary Figure S7D**), which overlap at the same time the ASOs previously used, and another four targeting a unique region at the gene’s end (long isoform; L1–4) (**Supplementary Figure S7E**). We analyzed the knockdown efficiency of the above experiments by qPCR using a primer pair amplifying a region only at the transcript’s end.

Overall, the LNA gapmers did not suppress KDM7A-DT expression to the same extent as the initially used ASOs. However, because LNAs show nuclease resistance and robust binding, their ‘gapmer’ design is associated with lower cell toxicity and a better phenotype than ASOs with extensive complementarity (66). Hence, we selected the LNA gapmer-based gene targeting approach for all subsequent *silencing* experiments. The LNAs that target the long isoform showed a moderately more robust knockdown than those that target the intermediate/truncated-sized ncRNA region (**Supplementary Figures S7D, S7E**). Among LNAs targeting the intermediate/truncated-sized isoform, only IS2 significantly affected γH2AX expression (**Supplementary Figure S7F**). Conversely, three out of four LNAs targeting the long isoform (L2, L3, and L4) led to increased levels of γH2AX (**Supplementary Figure S7G**). When we treated T47D cells with H_2_O_2_ for 30 minutes, and subsequently with the above four LNAs and a control LNA, γH2AX levels were upregulated in all samples, including the negative control LNA, due to OS pretreatment (**Supplementary Figure S7H**).

Subsequently, we conducted a microarray analysis of gene expression changes upon KDM7A-DT knockdown with LNAs in T47D cells compared to negative controls. The statistically significant results of differential expression analysis across all groups are presented in **Supplementary Table S2C** (see Methods; the criteria for extraction of DEGs were *p*-value< 0.05 and -0.3< log_2_FC > 0.25). Our comprehensive analysis unveiled distinct transcriptional responses contingent upon varying conditions. KDM7A-DT knockdown (Group B; **Supplementary Table S2C**) resulted in the upregulation of 3 genes, with 14 genes exhibiting downregulation. Interestingly, introducing OS after knockdown, denoted by treatment with H_2_O_2_, led to a notable shift in gene expression patterns. Specifically, 39 genes were upregulated, while a significantly higher number of genes, 342, were downregulated (Group C; **Supplementary Table S2C**). Furthermore, the knockdown effect in cells already subjected to stress (treatment with H_2_O_2_) demonstrated a distinct impact. Notably, 25 genes exhibited upregulation, while downregulated genes surged markedly to 1282 (Group D; **Supplementary Table S2C**). These findings underscore the dynamic interplay between KDM7A-DT knockdown and oxidative stress, elucidating the intricate regulatory mechanisms governing gene expression in response to environmental cues.

The specific design of knockdown experiments in T47D allowed us to delve into the functional aspects of KDM7A-DT’s role in oxidative DNA damage and genetic reprogramming within a cellular context where the abundance of lncRNA is highly promoted by the affected biological pathways, enhancing the relevance and applicability of our findings to the intricacies of the luminal A subtype.

Notably, KDM7A-DT’s downregulated pathway analyses (KEGG, String-12, EnrichR) were predominantly related to metabolism, including ‘oxidative and biosynthetic reactions like coenzyme metabolic process’ and ‘cellular respiration,’ while upregulated pathways were primarily associated with immune responses, such as ‘interleukin-2 production’ and ‘regulation of interferon-gamma production’.

### Differential pathway analysis after KDM7A-DT knockdown in T47D cells versus *KDM7A-DT* overexpression in nonmalignant fibroblasts

**Figure 8:**
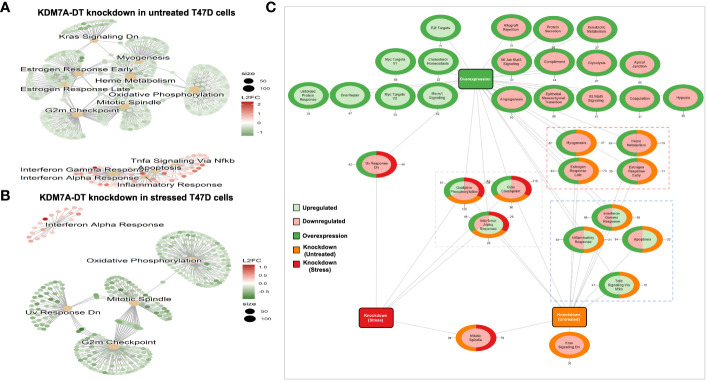
Differential enrichment of MsigDB cancer hallmark genesets after altering endogenous expression levels of KDM7A-DT shows an association between KDM7A-DT and numerous processes that interact with the stress response and metabolic processes. **(A, B)** GSEA results depicted as a cnetplot (category network) using cancer hallmark gene set terms. GSEA was based on log_2_FC resulting from differential expression analysis (expression quantified using microarrays) in KDM7A-DT knockdown T47D cells *versus* control T47D cells under normal conditions (group B) or stress conditions induced by H_2_O_2_ (group D). The significant gene sets that include upregulated and downregulated genes are represented and colored based on gene log_2_FC. The scale of dot sizes is based on gene set size. **(C)** Network depiction of differentially enriched cancer hallmark gene sets (upregulated or downregulated) after altering endogenous expression levels of KDM7A-DT (overexpression in MRC5 cells, knockdown in untreated T47D cells, or knockdown in T47D cells under stress conditions induced by H_2_O_2_). The color inside the circles represents the enrichment score of the corresponding gene set (light-green > 0, light-red< 0) in each category, illustrated with the border color (green: overexpression, orange: knockdown (group B), red: knockdown (group D), while the number indicates the enriched DEGs.

The 50 hallmark genesets (GSEA) from MSigDB were used to complement our GO analysis of KDM7A-DT knockdown in T47D. This analysis revealed the *activation* of cytokine production pathways (interleukin-2, interferon-alpha, interferon-gamma, TNFα signaling via NF-κB, and inflammatory response pathways) and the *suppression* of metabolism-related pathways (heme metabolism, oxidative phosphorylation, cellular respiration), cell signaling (estrogen response early/late, myogenesis, *Kras*) and cell cycle-related pathways (mitotic spindle, G2/M checkpoint) (**Figure 8A**). Interestingly, four of the above pathways, the interferon-alpha response, oxidative phosphorylation, mitotic spindle, and G2/M checkpoint, were concomitantly regulated in KDM7A-DT knockdown cells under OS (**Figure 8B**). Furthermore, two of the above pathways (oxidative phosphorylation and G2/M checkpoint) were concomitantly positively regulated by KDM7A-DT’s forced expression in MRC5 cells (**Figure 8C**), and four (interferon-alpha, interferon-gamma response, inflammation response, and apoptosis) were concomitantly negatively affected by KDM7A-DT in MRC5 and T47D cell models.

The perturbed expression of KDM7A-DT revealed downregulated DEGs abundant in estrogen response, myogenesis, and heme metabolism pathways in T47D knockdown cells that were inversely regulated in MRC5 cells over-expressing KDM7A-DT. In contrast, the upregulated DEGs in T47D knockdown and MRC5 over-expressing KDM7A-DT cells were enriched in only one pathway (TNFα signaling *via* NF-κB). DEG’s profile of T47D cells does not show enrichment with the *EMT pathways* of MSigDB Hallmark cancer genesets and also breast cancer genes EMT signature (67). To determine the frequency of gene occurrence by chance, we randomly generated 100 gene subsets (chosen from **Supplementary Table S2**) of the same sample size as the tested gene set. Results of EMT enrichment analysis in each group C or D (**Supplementary Table S2C**) confirmed this enrichment analysis and suggested that KDM7A-DT has no role in EMT when it is downregulated in T47D cells. **Supplementary Table S10** and the **Supplementary Information** section provide more details of the comparison analysis.

### KDM7A-DT knockdown activates the NHEJ branch of the DDR/R signaling in T47D BRCA cells

In this study, we analyzed the dynamics of γH2AX in the T47D cells to build an oxidative DNA damage BRCA cellular model where KDM7A-DT is validated by northern blot analysis to be endogenously over-expressed. We used western blotting for the detection of γH2AX in T47D cells treated for 30 minutes, 2 hours, and 24 hours with H_2_O_2_ (0.2 mM) and for 2 and 24 hours with two other DNA damage-inducing agents [doxorubicin (1 mM) and etoposide (10 μM)] as reference DNA damage-inducing agents (**Supplementary Figure S8A**). The highest γH2AX signal levels occurred after 30 minutes and 2 hours of H_2_O_2_ treatment, while in the case of doxorubicin and etoposide, the highest γH2AX signal was detected 24 hours after treatment (**Supplementary Figure S8A**). These data indicate that in T47D cells, OS is a faster-acting DNA damage-inducing agent than the two reference agents. To decipher the dynamics of the two major branches of the DDR/R pathway (NHEJ and HR) in T47D cells in response to oxidative DNA damage, we analyzed the levels of RAD51 (a protein known to facilitate progression to HR) and TP53BP1 (a protein playing a significant role in DSB and is accumulated in DNA damage sites during NHEJ) in a time-course experiment. We analyzed the expression of corresponding proteins by western blotting before and after treatment with H_2_O_2_ (0.2 mM) for 30 minutes, 2 hours, and 24 hours. TP53BP1 protein levels were significantly upregulated after 30 minutes and 2 hours of OS. Furthermore, after 2 hours, we observed three (3) highly abundant TP53BP1 isoforms (splice variants), while two less abundant TP53BP1 isoforms were observed after 30 min and 24 hrs. In contrast, OS treatment did not affect RAD51 protein levels. The levels of both TP53BP1 and RAD51 were significantly reduced during long-term H_2_O_2_ treatment (24 hours) (**Supplementary Figure S8B**).

**Figure 9:**
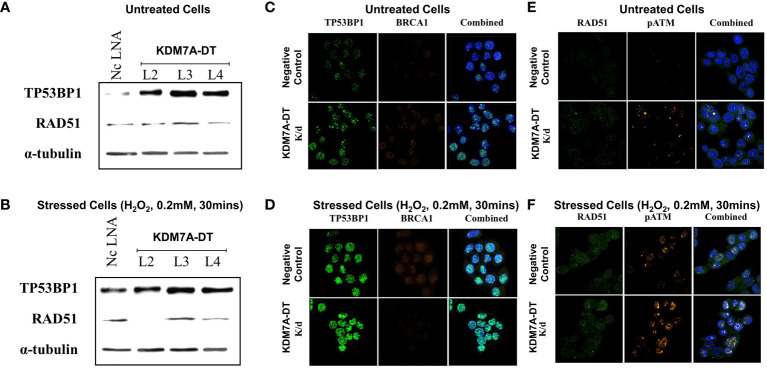
KDM7A-DT knockdown in T47D BRCA cells activated the TP53BP1-mediated NHEJ response, similar to OS, while neither KDM7A-DT expression nor OS affected the HR pathway. **(A)** The LNA gapmers (L1, L3, and L4) were used to knock down full-length KDM7A-DT transcript in untreated T47D cells, resulting in significant upregulation of TP53BP1 protein levels compared with the corresponding levels in the negative control (Nc) LNA cells, as analyzed by western blot. **(B)** In T47D cells treated with 0.2 mM H_2_O_2_ for 30 minutes, TP53BP1/53BP1 protein levels were strongly upregulated compared with those in Nc LNA cells. Cells transfected with all three LNA gapmers (L1 - L4) and treated with 0.2 mM H_2_O_2_ for 30 minutes showed a milder relative upregulation of TP53BP1 protein levels compared with the relative upregulation of TP53BP1 levels in H_2_O_2_-untreated L1 - L4 knockdown cells Double-immunofluorescence experimental data in pairs are presented in figures **(C-F)**. The 1st pair, TP53BP1 (green color signal) and BRCA1 (gold color signal) are major signaling proteins governing DSB repair via the NHEJ pathway. **(E, F)** The 2nd pair, RAD51 (green color signal) and phosphorylated ATM (gold color signal), are essential proteins and markers in DSB repair via the HR pathway. **(C, E)** KDM7A-DT knockdown T47D (L1-L4 gapmer) BRCA cells under untreated conditions; **(D, F)**. KDM7A-DT knockdown T47D (L1-L4 gapmer) BRCA cells after 30 minutes of OS (H_2_O_2_, 0.2 mM) compared with control cells (Nc LNA).

KDM7A-DT knockdown by all selected LNAs (L1, L3, and L4) in T47D cells resulted in a significant upregulation of TP53BP1 but not RAD51 protein levels (**Figure 9A**). In addition, early OS treatment (H_2_O_2_, 0.2 mM, 30 minutes) resulted in the upregulation of TP53BP1 protein levels compared with untreated control cells (**Figures 9B**; **Supplementary Figure S8C**) and had statistical insignificant effects on RAD51 protein levels in both untreated and stressed knockdown cells (**Figures 9B**; **Supplementary Figure S8B**). To gain further insight into the role of KDM7A-DT in DDR/R focus pattern formation within the nucleus, we conducted double-immunofluorescence experiments based on hallmark protein pairs involved in DDR/R, such as RAD51 with pATM and TP53BP1 with BRCA1. KDM7A-DT knockdown in T47D cells resulted in a robust upregulation of TP53BP1-containing DDR foci compared with control cells, while BRCA1 protein remained at low levels (**Figures 9C**; **Supplementary Figure S8C**). On the other hand, KDM7A-DT knockdown in OS-induced T47D cells resulted in a slight upregulation of TP53BP1-positive foci compared with control cells. At the same time, BRCA1 protein levels remained low (**Figure 9D**), which agrees with the western blot analysis above (**Figures 9A, B**). Thus, i) in OS-treated cells there is a slight upregulation of RAD51, which is diminished after KDM7A-DT knockdown; ii) in both the nontreated and OS-induced KDM7A-DT knockdown T47D cells, TP53BP1 forms microscopically visible DDR nuclear foci; iii) KDM7A-DT expression depletion in the nontreated and OS-treated cells upregulates the expression signal of TP53BP1-defined DDR foci, while BRCA1 expression exists in DDR foci at low levels in the nontreated and OS-treated KDM7A-DT T47D cells.

In untreated T47D cells (negative control, **Figure 9E**), RAD51 and pATM levels were very low or undetectable in cells. However, in KDM7A-DT knockdown OS-untreated T47D cells, the phosphorylation level of the ATM protein (**Supplementary Figure S8D**) and abundance in the DDR foci was increased (**Figure 9E**); however, the RAD51 signal showed a low intensity and diffuse distribution in the cytoplasm (**Figure 9E**; **Supplementary Figure S8B**). Furthermore, the RAD51 signal did not form DDR foci but was localized in the cytoplasm, validating that KDM7A-DT-mediated activation of DDR/R does not involve the HR branch (**Figure 9E**).

In agreement with the western blot analysis (**Figures 9A, B**), in both KDM7A-DT non-treated and KDM7A-DT knockdown T47D cells, activation of DDR/R (pATM) signaling upon OS treatment was present without detectable or at a low-level of RAD51 accumulation in the nucleus and DDR foci (**Figure 9F**). Hence, in the T47D BRCA cell line, KDM7A-DT knockdown activates the NHEJ branch of the DDR/R pathway, whose initiation events include cell cycle arrest, phosphorylation of ATM/H2AX proteins, and the accumulation of TP53BP1 protein in DDR foci, a hallmark protein of the DSB repair pathway.

### KDM7A-DT-defined genes that suppress the EMT Type II pathway in fibroblasts are commonly included in the EMT Type III oncogene pathway activated in BRCA tissues

Even though KDM7A-DT knockdown in T47D does not affect the EMT pathway, KDM7A-DT over-expression in nonmalignant fibroblasts downregulates it significantly. Based on our results, we hypothesized that increased KDM7A-DT expression in BRCA could be involved in developmental programs of cell type-specific lineage, particularly in the pathways inducing epithelial-to-mesenchymal (EMT) and mesenchymal-to-epithelial (MET) transitions. To elaborate this hypothesis, we extracted from TCGA BRCA data the genes whose mRNA levels positively and negatively correlate with KDM7A-DT expression. First, analyzing RNA-seq data, we found 4,491 genes positively correlated, and 3,402 genes negatively correlated with KDM7A-DT (**Supplementary Table S11**). Second, using the dbEMT-2 database (52), we identified 191 EMT pathway–associated genes classified as oncogenes and 260 EMT pathway–associated genes classified as tumor suppressors in these lists (**Supplementary Table S12**). We found that 47 of the 88 KDM7A-DT-defined EMT Type II pathway–associated gene signatures downregulated upon its over-expression in MRC5 cells were categorized as oncogenes and tumor-suppressors (**Supplementary Figure S9**, **Supplementary Table S12**). **Supplementary Figure S9** shows the Venn diagrams of KDM7A-DT-defined EMT Type II-associated genes classified as EMT pathway-associated oncogenes and tumor suppressors, positively and negatively correlated with KDM7A-DT in BRCA primary tumors. **Supplementary Tables S12A, B** shows the gene lists represented by distinct gene groups defined in the Venn diagrams. We observed that 44 out of 47 EMT Type II genes (**Supplementary Figure S9A**) were significantly positively correlated with KDM7A-DT expression in BRCA samples. In contrast, the expression of only 3 of the 47 EMT Type II genes (**Supplementary Figure S9B**) demonstrated significant negative correlations with KDM7A-DT expression in the same patient cohort (*p* = 4.27E-08, Fisher exact test). We found a significant EMT Type III pathway–associated gene enrichment in the EMT oncogene subset (one-sided *p* = 0.048, ratio of two binomial proportions exact test). However, there was no statistical significance in the case of the tumor suppressor gene subset (one-sided *p* = 0.34, ratio of two binomial proportions exact test). These findings suggest that in nonmalignant fibroblasts, overexpression of KDM7A-DT leads to downregulation of EMT Type II pathway genes that are frequently activated in BRCA samples where KDM7A-DT is also over-expressed, especially a subset enriched for EMT Type III-associated pathway genes, collectively referred to as highly aggressive and drug-resistant BRCA.

We relied on the 46 KDM7A-DT-associated mRNA/protein pairs (**Table 2**) concordantly expressed in the TCGA BRCA cohort to verify this hypothesis. Using IPA, dbEMT-2, and EMTome databases, we found that eight (8) of these genes are known as EMT pathway-associated genes (**Table 2**). The mRNA and protein of the seven (7 out of 8) genes mentioned above (*NOTCH1*, *WWTR1*, *ETS1*, *YAP1*, *EGFR*, *AKT3*, and *TGM2*) are annotated as EMT pathway upregulated genes involved in oncogenesis and cell invasiveness. Transglutaminase type 2 (*TGM2*) was included in our KDM7A-DT EMT Type II downregulated gene signature in fibroblasts. The set of 46 KDM7A-DT-associated genes, whose mRNA and protein expression levels are concordant, includes the *SMAD3 gene*, which encodes a well-characterized tumor suppressor protein playing an essential role in TGF-beta signaling and core EMT pathway. We found a significant negative correlation between SMAD3 transcript/protein and KDM7A-DT expression levels (**Table 2**). Furthermore, SMAD3 is a component of the TGF-β signaling pathway and contributes to G1 phase cell cycle arrest in BRCA cells. SMAD3 may act as a tumor suppressor by blocking c-Myc transcription and stimulating p15 production to activate G1 arrest. We found that *SMAD3* expression exhibits negative correlations with many Cancer Hallmark gene-subsets expression compared to KDM7A-DT, which shows positive correlations with genes primarily representing the EMT score and many Cancer Hallmark gene-sets (*p*< 1.00E-09). Notably, *SMAD3* expression was negatively correlated with the expression levels of the following gene subsets: c-Myc targets V1/V2, the unfolded protein response, oxidative phosphorylation, the ROS pathway, mitotic signaling, the G2/M checkpoint, E2F TF targets, DNA repair, UV response down, cholesterol homeostasis, and glycolysis (**Supplementary Table S13**). However, *SMAD3* expression is positively associated with the UV response and (early and late) estrogen response. We performed a survival prediction analysis of *SMAD3* expression defined by Affymetrix U133A probe sets (205396_at) in 4934 BRCA samples collected in the K–M Plotter database. This analysis showed that higher expression of *SMAD3* identifies the risk group with relatively favorable outcomes (by RFS) at a high confidence level; we reproduced this result in BL and other BRCA subtypes defined by the StGallen classification (**Supplementary Figure S10**). Based on the above findings, we suggest that KDM7A-DT may be involved in the suppression of a specific gene set of EMT-mediated pro-oncogenic signaling in non-malignant, that is activated in BRCA cells with KDM7A-DT over-expression that may lead to uncontrolled cell growth and invasiveness, poor prognosis and drug resistance. However, interactions between SMAD3/2 and SMAD4/2 complexes and TGB-β signaling at the KDM7-DT/KDM7 promoter region are possible (68), which may regulate KDM7DT activity at transcriptional and post-transcriptional levels. The molecular mechanisms leading to a negative correlation between SMAD3 and KDM7A-DT-defined signature EMT phenotype in BRCA patient samples, as well as the role of SMAD3 - KDM7A-DT axis in survival prediction, may be clinically promising and need to be studied.

### KDM7A-DT-defines activating pro-oncogenic EMT (Type III) genes that separates breast cancers into distinct subgroups

Following the above notion, we hypothesized that up-regulated EMT Type II BRCA DEGs can also include elements of the 88 gene EMT Type II signature down-regulated by overexpression of KDM7A-DT transcription. Using gene expression profiles of post-EMT cell derivatives derived from mammalian cell lines after their transformation into mesenchymal-like cells and cell sorting, 265 DEGs involved in up- (n=134) and down-(n=131) regulation of the mammary gland EMT pathway were selected. Next, we confirmed that these 265 EMT DEGs were a unique priority to mesenchymal vs. epithelial BRCA genes. This statement has been supported using analysis of the molecular subtypes DEG clusters of the 51 breast cancer microarray profiles (**Supplementary Table S14**; **Supplementary Materials**) and EMT databases used in this study (Websites/databases/software; **Supplementary Materials**). No Type II genes were in the list of 131 down-regulated post-EMT derivatives DEGs generated from mammalian cell lines.

In contrast, 17 of the 134 up-regulated post-EMT derivatives DEGs generated from mammalian cell lines were presented in our 88 DEG EMT Type II signature (*SERPINE1, PLOD2, CCN2, CCN1, WNT5A, COL4A2, COL4A1, ITGA5, MYL9, FBLN1, FBLN5, SPOCK1, COL1A1, COL1A2, COL5A2, CALU* and *FERMT2*) (**Supplementary Table S14A**; **Supplementary Materials**). These 17 genes of EMT type II signature may also consist of a pro-oncogenic BRCA EMT signature (BRCA EMT Type III subset). In summary, it appears that KDM7A-DT-defined genes suppressing EMT Type II pathways in fibroblasts function as pro-oncogenes in BRCA cells that activate EMT Type III pathways driving cancer progression.

## Discussion

### Stress-induced and TP53-dependent transcriptional regulation and biogenesis of KDM7A-DT

Si-paancRNAs are induced by OS due to a transient accumulation and bi-directional pausing of RNAPII at divergent promoters of stress-specific genes. Strikingly, overexpression of all four selected si-paancRNAs promoted, independently and significantly, activation of γH2AX-mediated DDR/R signaling and p53/p21-mediated cell cycle arrest in nonmalignant proliferating lung fibroblasts (MRC5 cells), and an overall stress-associated cellular morphology. To screen for the ability of si-paancRNAs to be induced in the context of critical cancer pathways, p53, and p16/*INK4* protein levels were knocked down in semi-immortalized skin fibroblasts. Among the four si-paancRNAs selected above, KDM7A-DT showed a transcriptional super-induction upon OS and, in parallel, a strong dependency on *TP53* knockdown. This means that endogenous levels of TP53 may strongly inhibit KDM7A-DT induction by OS. Overexpression of KDM7A-DT in the nonmalignant lung fibroblasts resulted in the activation of oxidative phosphorylation, G2M checkpoint arrest, and the inhibition of glycolysis and heme metabolism pathways. These findings concord with a recent study in children with sickle cell anemia where KDM7A-DT expression levels were correlated with hemoglobin subunit gamma-1 and gamma-2 expression (69). The study’s findings suggest that si-paancRNA KDM7A-DT in BRCA is (i) a robust genetic signal of TP53 missense mutation associated oxidative DNA damage and (ii) a lncRNA driver of subtype-specific oncogenesis and progression. In pathological conditions, it could be accumulated by genetic amplification and stress-associated transcriptional «training» to modify cellular signaling pathways (tolerance vs. sensitivity) by regulating the NHEJ in the DDR/R pathway.

We provide insight for the first time regarding the biogenesis of si-paancRNAs in response to OS, using KDM7A-DT as a case study. We showed that its complex biogenesis is derived from stress-induced promoter-associated transcriptional interference and alternatively processed 5’ and 3’ end sites. KDM7A-DT expression levels are elevated within its locus’s 250–1000 bp region, whereas the highest relative expression levels are within the 1250–2230 bp window. These results could be partly explained by the co-localization, at the 250-750 bp region, of a highly active divergent core promoter region that, in response to OS, is causing RNAPII-mediated noncoding interference. This, combined with the existence in the KDM7A-DT locus of stress-induced alternative 5’ and 3’ sites, possibly generates an abundance in size variation of truncated/intermediate-sized ncRNAs with primary cytosolic enrichment. In contrast, the expression of the primary regulatory lncRNA is weak, but it is OS-specific and shows a nuclear enrichment and a subtype-specific expression pattern in BRCA.

This regulation of the long KDM7A-DT isoform may indicate that the stress-specific promoter of KDM7A-DT and its gene locus show transcriptional biogenesis similar to dilncRNAs. Our hypothesis is supported by a recent study that showed that independent of the location of the DNA lesion, DSBs act as transcriptional promoters, creating both DDRNAs and dilncRNAs that interact (66). Such RNA–RNA interactions could explain why silencing KDM7A-DT by targeting the region specific to the long isoforms and not the common seed region of all isoforms was more efficient and consistent across experiments. Whether dilncRNAs and si-paancRNAs represent one or two distinct classes of p53-mediated lncRNA response to recruiting TP53BP1 in non-coding (low homology) regions remains unclear.

KDM7A-DT is the antisense RNA gene of the protein-coding gene KDM7A (or JHDM1D) with head-to-head gene architecture. They share common promoter regions and transcriptional regulatory sites. *KDM7A* encodes a Jumonji domain-containing histone demethylase essential for brain development, participating in epigenetic gene regulation, c-Myc activation, and apoptosis (68). According to our analysis of TCGA-Pancancer data, *KDM7A* mRNA is the top positively correlated gene of KDM7A-DT (*r* = 0.66, *p*< 1.00E-98). It has been shown that genomic deletion of the KDM7A-DT promoter partially downregulates KDM7A-DT and *KDM7A* expression (38). In our experiments, KDM7A expression was moderately upregulated by KDM7A-DT overexpression (*p*=0.042) in MRC5 cells and downregulated after KDM7A-DT knockdown in T47D cells (p = 0.064). These data suggest that there might be an interplay between KDM7A and KDM7A-DT gene locus. However, unlike KDM7A-DT, KDM7A is not survival-significant in BRCA (data not presented), which implies that KDM7A’s role is independent of KDM7A-DT in BRCA progression and BRCA outcomes.

### KDM7A-DT-mediated global reprogramming of non-malignant fibroblasts

KDM7A-DT overexpression resulted in significant changes to the transcriptome that can be dichotomized in up-and-down-regulated genes associated with distinct cellular processes and pathways. Interestingly, the top upregulated genes due to KDM7A-DT over-expression were significantly enriched in regulating transcription activity and RNA metabolism pathways. In contrast, the top-downregulated genes were significantly enriched for cellular communication. The GSEA analysis revealed that KDM7A-DT overexpression led to a significant enrichment of cancer hallmark gene sets, primarily related to cell cycle regulation and c-Myc targets, further supporting the potential oncogenic function of KDM7A-DT. STRING enrichment analysis further supported that KDM7A-DT-mediated global reprogramming of non-malignant fibroblasts can induce significant changes in gene expression patterns associated with cellular proliferation, angiogenesis, and tumorigenesis.

In addition, the EMT pathway–associated genes were the most downregulated gene set in MRC5 cells, suggesting its role in suppressing myogenesis, coagulation, angiogenesis, apical junction, protein secretion, immunity, and inflammatory processes. In a recent study, experiments of overexpression and knockdown of KDM7A-DT in monocytes imply that this lncRNA influences cell development and homeostasis pathways. Furthermore, the above research proposes that KDM7A-DT acts as an inflammatory response regulator, which aligns with our findings (70). It is well known that stressed cells first activate the DDR/R pathway to perform DNA damage assessment, then enter the S-phase and initiate mitosis, arrest, or apoptosis. We found that KDM7A-DT overexpression in non-malignant fibroblasts induces a γH2AX-mediated DDR/R response that activates the G2M checkpoint arrest and TNFa-signaling via NFkB but inhibits the estrogen response (early and late), myogenesis, heme metabolism, apoptosis, interferon, and inflammatory responses.

### KDM7A-DT can play a pro-oncogenic role in luminal A breast cancer cells

Interestingly, the endogenously overexpressed KDM7A-DT in T47D cells presented a partially inverted regulatory pattern, where DDR/R signaling, apoptosis, interferon, and inflammatory response were down-regulated, but the G2/M checkpoint, estrogen response (early and late), myogenesis, heme metabolism and TNFa-signaling via NFkB were upregulated. Additional immunofluorescence experiments verified that upon KDM7A-DT knockdown, the NHEJ-TP53BP1 axis of the DDR/R pathway is induced to repair SSBs and DSBs, while the HR branch is unaffected. OS acts similarly by inducing NHEJ and not affecting HR. It is important to mention that a 40%–60% decrease in KDM7A-DT levels was sufficient to generate these effects. In conclusion, endogenous KDM7A-DT expression in BRCA T47D cells exerts oncogenic effects by suppressing the NHEJ protein pathways and activating pathways of G2M checkpoint, immune response, and oncogenic metabolism.

Accordingly, our comparative enrichment analysis in MRC5 and T47D cells suggests that KDM7A-DT is a component of multiple pathways regulated concomitantly or inversely in MRC5 and T47D cells. However, the interpretation of the effects on downstream pathways resulting from KDM7A-DT knockdown in T47D cells, used as an *in vitro* 2D growing cell line model of the luminal A subtype, is subject to limitations within the scope of this study’s objectives. Generally, the 2D growing cell line models lack many key characteristics and the complexity of cancer’s origin and progression, including the diversity of cancer at the molecular level, signaling pathways, cellular compartment heterogeneity, and tissue diversity. These factors play essential roles in studies involving patient samples and in understanding the outcomes of the disease.

### T47D cells as a model to identify the functional roles of endogenous expression of KDM7A-DT in BRCA

We observed that TCGA data demonstrate a spectrum from moderate to high levels of KDM7A-DT expression in a large proportion of BRCA primary tumors. Also, our results showed that the KDM7A-DT expression pattern can be varied in the subtype contexts and is associated with the TP53-missense mutations. Cell lines with different phenotypic and intrinsic molecular subtype characteristics are widely used in breast cancer research (67). We analyzed six (6) cell lines to identify which one(s) show stable and abundant expression of KDM7A-DT. We used MCF7 and T47D (luminal A), SKBR3 (HER2), Hs578T, MDA-MB-468, and MDAMB-231 triple-negative/basal-like BRCA subtypes. SKBR3, Hs578T, MDA-MB-468, and MDAMB-231 cells represent aggressive BRCA cells with TP53 mutations (71). MCF7 and T47D cell lines retain several key characteristics specific to the mammary epithelium with luminal cell properties. Remarkably, MCF7 cells are TP53 mutation-negative, while T47D cells exhibit a TP53 missense mutation.

Previous studies have shown that T47D is more susceptible to progesterone compared to other popular breast cancer luminal cell line MCF7, which also has a metastatic cell origin (72). TP53 mutations have responsibilities in the endocrine response, genome instability, and drug resistance pathways; it is a rare event in the luminal A subtype of patient BRCA samples. Among the cell lines tested, only T47D cells showed high expression of full-length KDM7A-DT locus. The T47D breast cancer cell lines were originally isolated from the pleural effusion of the 54-year and 69-year-old female patients with metastatic disease (71). Although the phenotypic and molecular characteristics of MCF-7 and T47D cells are similar, hundreds of 2D- and 3D-growing T47D and MCF7 cell lines are differentially expressed (72).

In this study, the luminal A T47D cells were applied as a model to identify the functional roles of endogenous expression of KDM7A-DT in BRCA. To refer to clinical data, T47D cells may be considered a KDM7A-DT-highly expressed cell line model as a variant of many rare but clinically relevant *‘atypical’ aggressive and metastatic variants* of luminal A subtype metastatic cells that development is controlled by the ER, PR receptors, differentially expressed pathways defined by luminal lineage precursors origin. The T47D breast cancer cell line was originally isolated from the metastatic pleural effusion (71). These cells are negative for the BRCA EMT pathways (according to our results) and HER2 and are highly differentiated tumor cells. The growth of the cells is controlled by the hormonal expression of both ER and PR, reflecting the specific pathways defined by the original luminal precursor cells (72, 73). The 2D growth characteristics of T47D cells pose a challenge when attempting to extrapolate findings to 3D cultures and patient breast cancer (BRCA) tissues (74, 75). Unlike their 2D counterparts, T47D cells grown in three-dimensional cultures exhibit *varied morphologies*, form tightly cohesive structures with robust cell-cell adhesions, and display significant differences in their expression profiles (75). The limitations of *in vitro* models restrict the extrapolation of our cell line data to clinical cancer research studies, suggesting a multidisciplinary approach.

### KDM7A-DT expression associations with BRCA molecular and tumor heterogeneity

BRCA’s heterogeneity is diverse and a critical factor in this cancer type progression, response to therapy, and metastasis. The molecular signature across breast cancer subtypes does not appear to represent distinct states but rather transitional ones, capable of inter-conversion due to intratumor clonal and cell-state heterogeneity. Clonal heterogeneity is defined by a multi-level selection process of cloned populations within a tumor based on their phenotypic variance, which is affected by both spatial and temporal factors (70). Clonal evolution of a dominant or resistant clone is a potential mechanism for malignant spread and treatment resistance. Besides intratumor heterogeneity, breast cancer also shows increased intertumor heterogeneity among tumor tissues of different patients or different metastases (76). Changes in the genome, epigenome, transcriptome, and proteome heterogeneity can be reflected in various aspects of BRCA development, resistance to therapy and metastasis, such as biomarkers, metabolism, cell cycle, tumor microenvironment (TME), epithelial–mesenchymal transition (EMT), circulating tumor cells (CTCs), and clinical pathology.

Considering the significant heterogeneity in BRCA’s molecular, cellular, and clinical characteristics (77), we identified multiple associations between KDM7A-DT expression and most critical risk factors and biomarkers of BRCA, using TCGA primary BRCA sample data and independent microarray datasets. These analyses showed that increased KDM7A-DT expression levels correlate with advanced BRCA histologic grade and system-level aberrations, including aneuploidy, CNA, HR defects, tumor burden, lymphocyte count, intel neoantigens, and several other well-established cancer markers and scores of BRCA progression and heterogeneity.

### Possible mechanisms of overexpression of KDM7A-DT HER2 and BL vs luminal A and B subtypes

The overexpression of KDM7A-DT in HER2 and BL vs luminal A and B tumor subtypes can be explained by (i) significant enrichment of the gain and amplification of KDM7A-DT locus in the patient of HER2 and BL subtypes vs luminal A and B subtypes (ii) positive linear regression of CNA in order the groups with deletion, diploid, gain, and amplification events of KDM7A-DT locus with its expression, in BRCA samples with the TP53 missense (driver) mutations, (iii) inhibition and negative correlation of KDM7A-DT by endogenous TP53 mRNA levels and (iv) strong negative correlation of KDM7A-DT with ER’s mRNA and protein levels. These results suggest that the TP53 missense mutation event and other essential factors associated with genomic instability and ER signaling disruption may contribute to the relatively frequent overexpression of KDM7A-DT in BL and HER2 samples versus luminal A and B samples.

Following extraction of the TP53-missense mutation samples in each subtype patient’s group, we found that KDM7A-DT’s gene copy numbers were significantly higher in the BL and HER2 subtypes than in the luminal A and B subtypes. Thus, the mechanisms leading to higher KDM7A-DT expression in HER2 and basal samples compared to luminal A and B BRCA samples may be explained by TP53 missense (driven) mutation, gain, and amplification of KDM7A-DT genes. **Supplementary Table S10B** shows that count-only mutation event provides a discriminative subtype biomarker because such an event occurs in 62.1% of luminal A, 23.1% of luminal B, 4.3% of HER2, and 6.3% of BL subtype events. Regarding TP53 missense mutations, we also observed that the corresponding expression levels of TP53 mRNA levels were lower in Basal and HER2 subtypes patients compared to the luminal A and B subtypes patients. In contrast, the corresponding HRD scores in TP53 missense mutation samples rank the subtypes in a gradual order of luminal A and luminal B, HER2, and Basal (highest value). This may imply that an epigenetic down-regulation of TP53 mRNA levels could also be involved in the expression of KDM7A-DT stress-associated transcription.

Luminal breast cancers account for about 60% of all cases. They are HR-positive (HR+, ER+/-, PR+/-) and can be further classified based on HER2 receptor status (78). Luminal A breast, in total, is a less aggressive HER2^-^ (HR+/HER2−) subtype that can be characterized by the combinations (ER+/PR+, ER+/PR−, and ER−/PR+) of clinical statuses and relatively low proliferative and genetic grade signatures. Luminal B tumors tend to be relatively aggressive, demonstrating HER2−enrichment (HR+/HER2+) but could also be HR+HER−, encompassing ER+/PR+, ER+/PR−, and ER−/PR+ statuses. In addition, women with luminal B tumors are often diagnosed at a younger age than those with luminal A tumors, possibly indicating an association with a genetic risk factor (79, 80).

Relatively low KDM7A-DT expression in the luminal A and B subtypes vs. other subtypes could be explained by absent/weak associations of the KDM7A-DT expression with the pathways altered by *TP53 missense* and other mutations (such as aneuploidy, gains/amplifications, multiple rare mutations), subtype- associated tumor suppressors and oncogenes. In addition, mutations of other BRCA driver genes, such as GATA3 in luminal subtypes, may be responsible for down-regulating KDM7A-DT RNA levels in these BRCA contexts. On the other hand, the luminal subtypes have similar essential genetic programs/pathways, which are controlled by lineage-driving transcription and epigenetic (including ncRNAs) regulatory programs.

We observed a significant and reproducible negative association between the expression of lncRNA KDM7A-DT and the hormone receptor (HR) status (ER and PR) in BRCA cases: samples with upregulation of this lncRNA are mainly ER/PR negative. Even though KDM7A-DT’s expression was up-regulated in the HER2 BRCA subtype compared to the luminal subtypes, its RNA levels did not correlate with HER2 protein and ERBB2 transcript levels (data not shown). Historically, heterogeneity in breast cancer is understood in terms of varying expression of the ER, PR, and HER2. Recent studies have supported the link between miRNAs and lncRNAs and HR expression in patients with BRCA, highlighting their potential as prognostic biomarkers in hormonal-dependent cancers (81–83). In the whole BRCA cohort, KDM7A-DT overexpression is associated with poor BRCA outcomes across the entire BRCA cohort. Such results, however, are not clinically useful because complex molecular subtypes are present in a BRCA cohort, and the proportions of each subtype vary widely over different BRCA cohorts.

### Significant role of KDM7A-DT in survival outcomes overall and within each breast cancer subtypes

We found that KDM7A-DT can play a significant role in RFS, OS, and DMFS outcomes in BRCA. KDM7A-DT shows a robust association with many BRCA molecular risk scores and biomarkers. Since BRCA data exhibit high heterogeneity across tumors within the same subtype (67, 76, 79), we wanted to identify the role of KDM7A-DT’s aberrant expression in the variability of subtype-relevant outcomes. We divided the patients into low- and high-risk groups based on the KDM7A-DT expression cut-off values specified for each BRCA subtype and type of survival events in the primary tumor data. Our survival prediction analysis showed the different diagnostic and prognostic patterns of KDM7A-DT expression within subtype for basal HER2 subtypes compared to luminal A and B subtypes in the context of recurrence, progression, and metastasis events.

Basal-like BRCA (BLBC) has been characterized as the most genome-unstable, aggressive, highly metastatic, TP53-mutated BRCA subtype that accounts for almost 90% of deaths in BRCA (84). We found that KDM7A-DT stably reproduces a pro-oncogenic function at RFS, OS, and DMFS times. In this subtype tumor biology and clinical contexts, the pro-oncogenic role (poor prognosis) at higher expression KDMA7-DT within BLBC patients can be explained directly due to high enrichment of the gain and amplification event in triple-negative/basal type primary tumors and indirectly by TP53 missense mutations downregulating at transcriptional level TP53 mRNA levels, that (as we showed in semi-transformed fibroblasts model) can inhibit KDM7A-DT’s stress-induction levels. Also, according to our findings, the KDM7A-DT locus is gained/amplified, and correspondently, its full-length transcript over-expression is associated with the regulatory events/processes relevant to genotoxic stress development, tumorigenesis, hormone-independent tumor formation, aneuploidy, EMT/MET pathways, progression of TP53 missense mutation-associated clones within basal tumors.

KDM7A-DT expression pattern may serve as an important outcome marker (risk factor) in luminal A primary tumors. In luminal A subtype tumors, lower expression of KDM7A-DT is associated with better survival according to OS and DMFS data, but it was not a prognostic factor by RFS. Before discussing these results, we should note that luminal A subtype tumors are typically highly differentiated, often sensitive to hormone therapy, and exhibit low genetic grade and low proliferative gene signatures. Consequently, someone may propose a hypothesis of OS and DMFS homogeneity (like RFS) concerning KDM7A-DT expression. However, our results showed that when KDM7A-DT’s expression is relatively higher than a specific computationally cut-off value, a large proportion of the luminal A subtype patients were assigned to relatively high-risk group (53% (124/232) patients by OS and 72% (187/259) patients by MDFS). These findings suggest an underlying pro-oncogenic mechanism(s) relevant to higher expression of KDM7A-DT in luminal A BRCA. Notably, the low frequency of TP53 mutations in luminal A subtype BRCA primary tumors explains a small fraction of the poor outcome events. It suggests that other (unknown) mechanisms are involved in pro-oncogenic activity, which results in poor prognosis. According to our results, HRD scores are very low in luminal A tumors. This suggests that homologous recombination repair machinery damage may not be a significant explanatory factor.

Conversely, cases of KDM7A-DT upregulation are predominantly HR-negative or have low HR RNA and protein expression levels in tumors. These results suggest that high-level KRM7A-DT may act antagonistically to HR pathways in luminal A tumors, which could make cells chronically hormone-resistant. In our hypothesis, at persistent high KDM7A-DT expression, the luminal A tumors are at high risk of gaining progression and losing treatment control through ER/PR pathways.

In contrast to luminal A and Basal subtypes, our survival prediction analysis suggests that KDM*7A-DT* exhibits tumor suppressor functions in luminal B and HER2 subtypes. Hence, other genetic mechanisms of BRCA driver genes, such as GATA3, are likely related to KDM7A-DT’s subtype-specific tumor suppressor functions and require further study.

KDM7A-DT RNA levels alone may be an important diagnostic marker for RFS, OS, DMFS, and BRCA heterogeneity. However, its diagnostic and prognostic potential is significantly enhanced when analyzed alongside its partner genes. Specifically, the combined expression patterns of KDM7A-DT, KRT7, and GATA3 emerge as a promising three-gene prognostic signature for breast cancer. This triad, forming the KDM7A-DT-GATA3-KRT7 axis, is especially influential in delineating cancer cell subtype dynamics within the breast cancer cell population, with a marked impact on the BL subtype (see results). Our survival prediction analysis suggests that KDM*7A-DT* exhibits tumor suppressor-like functions in luminal B and HER2 subtypes. KDM7A-DT can play a role in different regulatory processes when it is restricted to HR and HER2-dependent epithelium origin BRCA subtype precursors and lineage cancer cells. The mechanisms underlying KDM7A-DT’s tumor suppressor-like functions in the context of BRCA patient survival are likely to be subtype-specific and require further research.

### Role of KDM7A-DT in EMT Type II-III pathways and potential clinical applications

The central role of aberrantly activated EMT pathway in aggressive BRCA is well documented and includes cell diversity, plasticity, metabolic reprogramming, stress response, drug resistance, cancer stem-cell-like phenotypes (26, 27, 49, 51, 52, 85, 86) TP53 tumor suppressor is critical for regulating the features mentioned above. This study identified functional links between *TP53* missense mutations and overexpression of the KDM7A-DT gene in BRCA. We presented the results suggesting a role of the TP53 mutations in the KDM7A-DT gene alterations and associated EMT processes (87). Our results indicate that KDM7A-DT upregulation in combination with KDM7A-DT-associated EMT Type II-III genes could be used to construct a KDM7A-DT-defined EMT Type III prognostic signature(s) of BLBC high-invasive and aggressive cancers. Furthermore, KDM7A-DT’s functional interplay with EMT could provide a mechanistic link with the DDR/R pathway. Understanding the complex interplay between EMT and DDR/R is key to dissecting the nuanced differences between luminal A and luminal B breast cancer subtypes. Luminal A subtypes typically maintain an epithelial phenotype, whereas luminal B subtypes are inclined towards a partial EMT, marked by a considerable rise in genomic instability and cellular proliferation.

As observed, the regulation of KDM7A-DT in gene expression and alterations is closely linked to BRCA heterogeneity associated with cancer progression. This stochastic process is influenced by the heterogeneity of cancer cells and cells within the tumor microenvironment (TME), such as fibroblasts and cancer-associated fibroblasts (CAFs). Tumor hypoxia is a common pathophysiological phenomenon that impacts intra- and intertumor heterogeneity, genomic instability, gene expression, apoptosis, and autophagy within the tumor mass and alters cell states. It reprograms cells in the TME, stimulating cellular metabolism, angiogenesis, EMT, and MET. Notably, MET in TME fibroblasts is a fundamental process in ontogenesis and oncogenesis. It has been demonstrated that hypoxia can reprogram the fibroblast genome towards epithelial-like cells through the EMT pathway, leading cancer-associated fibroblasts to undergo intermediate states and become more aggressive epithelial-like tumor cells (88). The role of the KDM7A-DT regulation in hypoxia-associated cancer progression mechanisms is perspective in the contexts of basic tumor biology and clinical needs.

We also identified a set of 17 KDM7A-DT-defined genes suppressing EMT Type II pathways in fibroblasts that, in BRCA, represent the activated EMT Type III (cancer) pathways. This signature gene set may be considered an EMT signature, separating breast cancers into distinct subgroups according to different progression stages and states of the EMT process.

Our correlation, network, and pathways analyses of KDM7A-DT-defined co-expression mRNA and protein profiles encoding TCGA DB breast cancer-associated genes identified high-confidence molecular interactions at RNA transcription regulation and protein-protein interaction levels. These results suggest that KDM7A-DT is involved in pathways and protein-protein interaction networks that are related to DDR, apoptosis, hormone and stress responses, transcription, tumor aggressiveness, and invasiveness, as well as essential BRCA oncogenes that showed links to subtype-specific mutational events (GATA3 in luminal, TP53 in Basal and HER2 subtypes).

In summary, KDM7A-DT and its long transcript exhibit several intrinsic biological and clinical characteristics that suggest their roles in invasive BRCA and its subtypes. KDM7A-DT-defined mRNA and protein subnetworks offer resources for identifying clinically relevant RNA-based signatures and prospective targets for therapeutic intervention. Our analysis suggests that a spectrum of genetic disruptions, including gene alterations of KDM7A-DT, chromosome-level alterations, aneuploidy, mutational burden, HR defects, and TP53 missense mutations, lays the groundwork for the activation of KDM7A-DT-defined chronic oxidative stress response pathways. This genetic and epigenetic framework facilitates the overexpression of KDM7A-DT, which inhibits the DDR/R and compromises the cell cycle, inflammation, metabolism, and apoptosis machinery, which are the driving factors affecting cellular heterogeneity, clonal progression and response to therapy in BRCA subtypes. The research further illuminates KDM7A-DT’s involvement in the complex EMT/MET dynamics of cancer progression, where cancer cells and their associated fibroblasts transition towards more malignant, epithelial-like phenotypes. These transitions are pivotal in modulating the immune response and metabolic processes, contributing to the development of drug resistance. Ultimately, this leads to the emergence of aggressive breast cancer subpopulations characterized by either high levels of KDM7A-DT in luminal A and basal-like/triple-negative forms or low levels in luminal B and HER2-positive types, highlighting the molecule’s critical role across different cancer subtypes.

### An explanatory model linking KDM7A-DT expression variation with TP53-dependent and TP53BP1-mediated NHEJ pathway dysregulation, aggressive phenotypic diversity, and prognosis variability

The ability of lncRNA KDM7A-DT to inhibit the TP53BP1-mediated NHEJ activity is of considerable theoretical and clinical interest. Cellular stresses stabilize and activate the TP53 signaling pathway, which regulates various cellular processes, such as apoptosis, DNA repair, and senescence. It is well known that inhibition of DDR activity may lead to the accumulation of unrepaired DSBs and, thus, to a chronic or unresolved activation of the DDR pathway, which results in a prolonged cell cycle arrest or senescence-associated proliferation arrest (89). Among the various broken DNA ends generated at DSBs, blunt ends, and short ssDNA subject to NHEJ repair, about 80% of DSBs can be repaired by NHEJ (90). TP53BP1 is an essential positive regulator of NHEJ‐mediated DSB repair. Recently, it has been shown that TP53BP1 accumulation in the nuclear foci is required for DNA damage‐induced cellular senescence via activation of the TP53 pathway in semi-immortalized fibroblasts (91). Remarkably, the same study showed that increased accumulation of TP53BP1 in the nuclear foci following DNA damage activates TP53 and governs cellular senescence via a liquid–liquid phase separation mechanism.

To our knowledge, our study reports for the first time that KDM7A-DT is super-induced following OS in semi-immortalized fibroblasts, and this response due to OS is TP53-dependent. We point out that overexpression of KDM7A-DT in normal cells activates the DDR/R and TP53 pathways to regulate cellular homeostasis and reprograms cellular capabilities to adapt to DNA damage. In addition, we showed that KDM7A-DT expression is upregulated in a TP53-dependent manner in response to an early *hras* oncogenic insult. However, this up-regulation wasn’t stabilized at later time points in the oncogenic-induced senescence model used. This could suggest that the gene’s function is more critical in the early stages of the response to oncogenic stress, such as initiating senescence, rather than in the maintenance or perpetuation of the senescent state.

There is no known role for si-paancRNA KDM7A-DT expression variation in NHEJ of DSBs in cancer subtype-specific contexts. Cancer is driven by oncogenic/genotoxic stress. In this context, oncogenes enhance DNA replication, leading to deregulated DNA replication. Consequently, the replication fork is stalled due to a shortage of such replication factors and tends to collapse (92). Deregulated DNA replication also increases replication‐transcription collisions (93). Uncoordinated DNA replication elicits DNA damage, which activates TP53 to induce senescence and prevent the proliferation of cells expressing oncogenes. However, in contrast to the tumor suppressor function of cellular senescence, some reports suggest that it increases oncogenesis, possibly by releasing factors that stimulate the proliferation and invasion of tumor cells (94). Hence, KDM7A-DT variations in BRCA may function as a tumor suppressor or pro-oncogenic using the TP53-TP53BP1-NHEJ pathway; their functions may be categorized as genetically, epigenetically, environmentally, and cellular in origin.

As a result of studies on patient tumors and tumor cell lines have demonstrated that chronically activated components of the NHEJ complex cause chromosomal aberrations, genomic instability, EMT, metabolic and epigenetic changes, especially in malignant cells with DSB repair defects (95). Our results and the current literature indicate that these phenomena could be related to KDM7A-DT functions. It has been reported that in high proliferative cells, NHEJ protein defects (and abnormal abundance) can be caused by incorrect repair of short sequences and error-prone short sequence accumulation, as well as by gaining/overexpressing of core NHEJ proteins (such as Ku70/Ku80) (95).

Differential expression of such core NHEJ factors has been found to correlate with cancer progression and overall survival (95). The majority of the studies have found that increased expression of nuclear Ku70/Ku80 and some other core proteins results in increased uncontrolled tumor proliferation, metastasis, basal subtype, and positive TP53 mutation status. These are leading risk factors for decreased survival outcomes.

However, some studies have found the opposite expression pattern. In breast cancer tissues, the lower expression levels of Ku70 and Ku80 proteins tended to be associated with a higher malignant nuclear grade of cancer cells and a higher frequency of axillary lymph node metastasis (96). Down-regulation of the Ku heterodimer is associated with the progression of bladder cancer from a low to a high malignant potential (97). Also, down-regulated Ku70 was associated with poor disease-free survival in colorectal cancers (98). Also, it has been shown that the mutations in specific NHEJ core complex molecules can dictate whether NHEJ alteration promotes or suppresses carcinogenesis and progression (95).

Thus, observations indicate that NHEJ is a complex process that acts as a guardian and a disruptor of genome and cellular processes. In T47D cells, we showed that full KDM7A-DT transcript significantly inhibits the TP53BP1-mediated NHEJ pathway while it activates the cell cycle arrest. Our results are limited to the luminal A cell line model. To validate these results and test the above hypothesis, extensive studies should be conducted to unravel the mechanistic link between KDM7A-DT, TP53, TP53BP1, and NHEJ factors and cellular homeostasis and heterogeneity in BRCA.

## Conclusion

Under the appropriate genotypic, epigenetic, and tumor microenvironment contexts, specific si-paancRNA may become an integral regulatory component of the cellular core stress response via regulating sensitivity/tolerance to oxidative DNA damage in non-cancer and cancer human cells. The above hypothesis is exemplified by the fact that the full-length KDM7A-DT can globally affect the NHEJ branch of the DDR/R protein pathway, cell cycle, and apoptosis, resulting in the activation of pro-oncogenic and oncogenic tissue-specific pathways in both the human fibroblast cell line MRC5 and the TP53-mutated BRCA T47D cell line, correspondingly. In BRCA patient tumor samples, the KDM7A-DT expression is linked to stress-induced pathways such as interferon response, TNFα signaling, aerobic and anaerobic metabolism, apoptosis, extracellular vesicle process, immune characteristics, and the EMT Type II phenotype. Locus-specific KDM7A-DT gain/amplification in association with *TP53* missense mutations and aneuploidy provides a genetic basis for chronic oncogenic stress-associated overexpression of full-length KDM7A-DT and poor clinical BRCA outcomes, specifically in the BLBC and luminal A subtypes. The expression of KDM7A-DT reveals its potential clinical value in differential and subtype-specific disease prognoses and diagnosis of BRCA subtypes. Following future verification, KDM7A-DT could be applied in personalized RNA-based signatures as a complementary score of BRCA heterogeneity and treatment outcomes.

## Methods

### Cell cultures

The following cell lines were used: MRC-5 (RRID: CVCL_0440), normal human lung fibroblasts; human embryonic kidney cell line 293T, which highly expresses the large T antigen (RRID: CVCL_0063); T47D (RRID: CVCL_0553), a TP53-mutation positive luminal A subtype BRCA cells [(ER(+)PR(+)HER2(-)]; luminal A cells MCF-7 (RRID: CVCL_0031) [ER(+)PR(+/-)HER2(-)] that does not have a mutation within the TP53 gene; the TP53-mutation positive HER2 subtype SK-BR-3 (RRID: CVCL_0033)], and the TP53-mutation positive triple-negative BRCA cell lines Hs578T (RRID: CVCL_0332), MDA-MB-231 (RRID: CVCL_0062), MDA-MB-468 (RRID: CVCL_0419) that can also specified on normal-like (Hs578T, MDA-MB-231) and Basal-like (MDA-MB-468) phenotypes. We also used a modified BJ Human fibroblast (RRID: CVCL_3653) immortalized by joint expression of the Telomerase reverse transcriptase subunit (hTERT), epiregulin (a growth factor of epidermal growth factor family) and the early region of SV40, which encodes for the viral large and small T antigens and conditionally over-express HRAS, by a 4-OHT-inducible HRAS vector. These cells and corresponding stable knock-down cells of TP53 and p16/INK4 genes were gifts from Dr. Mathijs Voorhoeve. All cells were cultured in a 5% CO_2_ atmosphere at 37 °C in a standard medium supplemented with 10% fetal bovine serum (FBS) and penicillin/streptomycin. All cell lines were maintained by passaging 1:3 when confluent in DMEM plus 10% FCS plus antibiotics in 5% CO2 at 37°C. Cell lines were seeded at subconfluent numbers (80%–90%) and treated for 30 minutes or 2 hours with 0.2 mM H_2_O_2_. All cell lines used in this study have been authenticated regularly according to standard quality control. Cells from the ATCC or other sources were authenticated by human Short Tandem Repeat (STR) profiling (ATCC). In addition, cell lines were confirmed to be free of Mycoplasma regularly using a PCR Mycoplasma kit (MD Bioproducts).

### Cloning

Three of the four si-paancRNAs (NR_036539, NR_002799, and NR_036530) studied were cloned into the pCDH-EF1-MCS-(PGK-GFP-T2A-Puro) lentiviral expression vector (System Biosciences) using cloning primers (**Supplementary Table S1**). KDM7A-DT was synthesized by DNA 2.0 (Newark), and its sense strand, as well as its reverse complement strand, were subcloned into pCDH-EF1-MCS-(PGK-GFP-T2A-Puro), a lentiviral (LTV) expression vector (System Biosciences).

### Preparation of lentiviral stocks and transduction

The stable expression of si-paancRNAs was obtained by cloning selected lncRNA transcripts into pCDH-EF1-MCS-(PGK-GFP-T2A-Puro). EF1 and PGK in a divergent configuration provide constitutive transcription of si-paancRNA transcripts. The SV40 and BGH polyadenylation signals at both ends of the vector enable efficient transcription termination, resulting in high steady-state expression levels. In the negative (antisense) orientation of the above expression cassette is the expression of copGFP (fluorescent reporter) and puromycin-*N*-acetyl transferase (drug-selectable marker) for detecting and selecting transduced cells. Third-generation lentiviruses were produced in Lenti-X 293T (Clontech) with a packaging mix of three constructs, pMDLg/pRRE, pRSV-Rev, and pVSVG, from Addgene. Supercoiled DNA constructs were prepared using QIAGEN maxi preps. For retroviral packaging, 293T cells were cotransfected with the retroviral particles. For transduction, LNCaP, PC-3, and DU145 cells were incubated with virus-containing supernatant in 8 mg/ml polybrene. After 48 hours, infected cells were selected for 72 hours with puromycin (1.5 mg/ml) or hygromycin (150 mg/ml). Integration rates were tested by quantitative polymerase chain reaction (qPCR) against the long terminal repeat (LTR) of pCDH and normalized to 1–2 integration sites per cell.

### Cell cycle analysis

Lentivirus-transduced cells were plated at a density of 60,000 cells/well 24 hours before analysis. Five hours before harvesting, cells were labeled with 10 μM EdU and processed using the Click-iT EdU Alexa Fluor 647 Flow Cytometry assay (Invitrogen). Cell nuclei were counterstained with 25 μg/ml propidium iodide (Sigma-Aldrich). Samples were subjected to EdU incorporation analysis on a BD FACSCalibur (Becton Dickinson). Data were analyzed using WIN-MDI 2.9 software.

### Transfection and lncRNA silencing

Chemically modified chimeric DNA ASOs and antisense locked nucleic acid (LNA) DNA gapmers were transfected using Lipofectamine 2000 according to the manufacturer’s instructions. According to the requirements of the transfection reagent, cells were added to 6-well plates 24 hours before transfection at 70% confluency. The transfection mixture in each well was prepared according to the kit’s instructions. Two hundred fifty microliters of serum-free modification of Eagle’s minimum essential medium (Opti-MEM, Gibco) containing 100 nM test ASOs or LNA or corresponding control ASOs/gapmers and 250 μl opti-MEM-containing transfection reagent were mixed and incubated at 37°C for 20 minutes. Then, it was added to each well containing 2 ml of DMEM with 10% fetal calf serum (FCS) and incubated for 4 hours. After incubation, fresh medium was added to all wells and incubated for 24 hours following the second round of transfection. Finally, all wells were incubated under standard culture conditions for 48 hours before harvesting the cells. The chemically modified chimeric DNA ASOs used in this study are shown in **Supplementary Table S1**. All three ASOs (392, 424, 457) were designed by IDT Technologies to target the beginning of KDM7A-DT. In addition, four antisense LNA gapmer sequences were designed by Exiqon for two independent regions of KDM7A-DT: the region that corresponds to the primary intermediate-size ncRNA transcript at the beginning of the transcript (IS-ncRNA; GRCh38/hg38, chr7: 140,177,302-140,177,832) and the region at the end of the transcript representing the full-length lncRNA KDM7A-DT (lncRNA; GRCh38/hg38, chr7: 140,178,951-140,179,640). A negative control LNA gapmer (Exiqon, catalog no. 300610) was also used.

### Western blotting

After preparing cell lysates, the protein concentration was determined with a bicinchoninic acid (BCA) kit. An equal amount of total protein content (20 μg) was resolved by SDS-PAGE and transferred to polyvinylidene fluoride (PVDF) membranes (Millipore) *via* a trans-blot semi-dry transfer cell system or wet-transfer using a mini-trans-blot electrophoretic transfer cell system (Bio-Rad Laboratories). For immunoblots, the primary antibodies used were rabbit anti-phospho-histone H2A. X (Cell Signaling Technology, Inc., catalog no. 9718S), rabbit ani-p21 Waf1/Cip1 (12D1) (Cell Signaling Technology, Inc., catalog no. 2947), mouse anti-p53 (Santa Cruz Biotechnology, catalog no. DO-1), mouse anti-phospho-ATM (Santa Cruz Biotechnology, catalog no. sc-47739), rabbit anti-53BP1 (Novus Biologicals, catalog no. NB100-904), mouse anti-BRCA1 (Santa Cruz Biotechnology, catalog no. sc-6954), and rabbit anti-RAD51 (Santa Cruz Biotechnology, catalog no. sc-8349). The secondary antibodies used were anti-rabbit IgG conjugated to horseradish peroxidase (HRP) and anti-mouse IgG conjugated to HRP (Santa Cruz Biotechnology). All blots were stripped and re-probed with monoclonal anti-α-tubulin antibody (DM1A, Sigma-Aldrich) as a loading control. Signals were visualized by ECL plus Western Blotting Detection Reagents (GE Healthcare) and a Kodak x-omat 1000A Film Processor.

### Immunofluorescence staining

Cells were grown at 37°C on coverslips in 12-well tissue culture plates in a culture medium containing 10% FCS (Life Technologies), antibiotics, and antimycotics. After reaching 70% confluence, the cells were washed with phosphate-buffered saline (PBS), fixed in 4% paraformaldehyde for 10 minutes, washed three times with PBS, and permeabilized with 0.25% Triton X-100 (PBST) at room temperature for 30 minutes. Next, cells were washed with PBS, blocked with 1% bovine serum albumin (BSA, Sigma-Aldrich) in PBST, and incubated with primary antibodies at 4°C (primary antibodies were diluted with 1% BSA PBST) overnight. After three washes with PBS, the cells were incubated in the dark with the corresponding fluorescein-conjugated secondary antibodies for 1 hour. The secondary antibodies used were goat anti-rabbit IgG conjugated to Alexa Fluor 488 (Invitrogen, catalog no. A11008), goat anti-mouse IgG conjugated to Alexa Fluor 488 (Invitrogen, catalog no. A11029), and donkey anti-mouse IgG conjugated to Alexa Fluor 594 (Invitrogen, catalog no. A21203). Subsequently, the cells were washed and mounted on slides. The nuclei were stained with 4’,6’-diamidino-2-phenylindole, and dihydrochloride (DAPI) using VECTASHIELD reagent (Vector Laboratories) and visualized using an LSM 710 confocal laser scanning microscope (Carl Zeiss) equipped with ZEN 2008 software (Carl Zeiss).

### Nuclear and cytosolic fractionation

A modified version of the Dignam protocol was used to prepare nuclear and cytosolic fractions. Briefly, 1 × 10^7^ cells were collected by scraping/centrifugation and washed twice in ice-cold PBS. Cell pellets were resuspended in 250 μl of ice-cold homogenization buffer (100 μM Tris-Cl pH 7.5, 15 mM NaCl, 60 mM KCl, 7.35 ml of 150 mM sucrose, 0.05% NP-40, 1 mM dithiothreitol (DTT), 10 mM vanadyl ribonucleoside complex (VRC, New England Biolabs), and proteinase inhibitors). One-fifth of the cell lysate (50 μl) was transferred to a new tube to isolate total RNA from whole-cell extracts. At the same time, the remaining lysate was applied to an equal volume of sucrose pad (100 μM Tris-Cl pH 7.5, 15 mM NaCl, 60 mM KCl, 300 mM sucrose, 1 mM DTT, 10 mM VRC, and proteinase inhibitors). Nuclear and cytosolic fractions were separated by centrifugation at 900 rpm at 4°C for 10 min (program without braking). Each supernatant (cytosolic fraction) was transferred to a new tube. At the same time, nuclear pellets were resuspended in 250 μl of wash buffer (100 μM Tris-Cl pH 7.5, 15 mM NaCl, 60 mM KCl, 1 mM DTT, 10 mM VRC, and proteinase inhibitors) followed by centrifugation at 14,000 rpm at 4°C for 30 minutes to remove cytoplasmic residue. One milliliter of TRIzol (Invitrogen) was added directly to whole-cell extracts, nuclear pellets, and cytosolic fractions.

### Northern blotting

Northern blot analysis used 20 μg of total, nuclear or cytoplasmic RNA from cells. RNA was separated by 15% denaturing urea-PAGE, transferred to a nylon membrane (Ambion), and UV cross-linked (Stratagene). DNA probes for different regions of KDM7A-DT and *GAPDH* transcripts were end-labeled with T4 polynucleotide kinase. The blot was processed following hybridization and washing. The primer pairs used to amplify the DNA probes are shown in **Supplementary Table S1**.

### RNA isolation coupled with reverse transcription-quantitative PCR

Total RNA was isolated from 1–1.5 × 10^6^ cultured cells with TRIzol reagent (Invitrogen). After treatment with RNase-free DNase I (Invitrogen), 1000 ng of total RNA was reverse transcribed using Superscript II First-Strand Synthesis Kit for RT-PCR (Invitrogen) under the conditions defined by the supplier. Complementary DNA (cDNA) was quantified by real-time PCR on the ABI Prism 7900 Sequence Detection System (Applied Biosystems) using SYBR Green PCR Supermix (Invitrogen). Each sample was run in duplicate, and each PCR experiment included two non-template control wells. Expression was normalized to β-actin (*ACTB*) and TATA-binding protein (*TBP*). The primers used for qPCR analysis are shown in **Supplementary Table S1**.

### Cell survival rate assay

The cell survival rate was detected using MTS (3-[4,5-dimethylthiazol-2-yl]-2,5 diphenyl tetrazolium bromide). The assay was performed as described previously. First, cells in the logarithmic growth phase were selected. Then, after being washed with PBS, cells were digested by trypsin and spread in a 96-well plate after dilution at a specific concentration (each well had approximately 5000 cells). Subsequently, the 96-well plate was placed in an incubator at 37°C overnight. After adding 20 μl MTS reagent to each well, the cells were cultivated in the incubator for 1 hour. Then, a multifunctional enzyme-labeling measuring instrument measured each well’s 490-nm optical density (OD) value. The cell survival rate was calculated based on the OD value: (mean OD_490_ value of experimental well)/(mean OD_490_ value of control well).

### Identification of the stress-induced KDM7A*-*DT cap-methylated and poly(A)-tailored sites

The stress-induced 5′- and 3′-ends of KDM7A-DT transcripts were detected using a modified rapid amplification of cDNA ends (RACE) that amplifies products specifically from capped and poly(A)-tailed mRNAs. We used a GeneRacer™ Kit and FirstChoice^®^ RLM-RACE Kit (Life Technologies) with total RNA isolated from MRC5 cells exposed to H_2_O_2_ (0.2 mM) for 30 minutes as a template. RT-PCR was performed with a 5′ or a 3′ RACE universal primer and corresponding gene-specific primers (GSP) for 5’ RACE (GSP1, GSP2) and reverse GSP primers for 3’ RACE (GSP1, GSP2, GSP3, GSP4, GSP5, GSP6), followed by ‘nested’ PCR with the nested universal primer and the nested gene-specific primers. PCR products were then gel-purified using a gel extraction followed by a PCR clean-up system (Qiagen). Finally, they were sequenced using the extracted bands or cloned into a TA cloning vector pCR^®^ 2.1 TOPO (Life Technologies) and sequenced individual colonies. 5′ RACE analysis revealed that stress-induced KDM7A-DT shows a primary 5′ cap located 224 bp downstream of its reported start site in RefSeq (NR_024451). In addition, it showed a secondary 5′ cap structure 247 bp downstream of its primary one. 3′ RACE analysis detected five stress-induced poly(A)-tailored transcripts, including the one predicted by the RefSeq annotation, generating 10 alternative intermediates-to-long ncRNA transcripts involved in the biogenesis and maturation of the KDM7A-DT transcript under OS. Full-length sequences of the two cDNA clones sequenced from 5′ RACE and the 10 cDNA clones from 3′ RACE analyses are shown in **Supplementary Figure S1**. The primers used for 5′ and 3′ RACE analysis and the resulting predicted sequences are shown in **Supplementary Table S1**. GSP1 followed by a nested GSP2 was used for reverse transcription for 5′ RACE.

### Microarray analysis

RNA was isolated, as mentioned above, from MRC5 cells overexpressing KDM7A-DT or vehicle control in triplicate. RNA quality and integrity were verified using the Agilent 2100 Bioanalyzer system (Agilent Technologies). Biotin-labeled cRNA samples for hybridization on an Illumina HumanHT-12 v4 Expression BeadChip (Illumina, Inc.) were prepared according to Illumina’s recommended sample labeling procedure. In brief, 500 ng of total RNA extracted from the cell samples was used for cDNA synthesis, followed by an amplification/labeling step (*in vitro* transcription) to synthesize biotin-labeled cRNA according to the MessageAmp II aRNA Amplification kit (Ambion, Inc.). The quality of cRNA was controlled using the RNA Nano Chip Assay on an Agilent 2100. Microarray scanning was performed using an Axon GenePix 4000B (Molecular Devices) scanner. All arrays were quantile normalized without background subtraction using Illumina GenomeStudio software.

### Differential gene expression

In a typical case, a significantly differentially expressed gene (DEG) is defined by an appropriate statistical test and selected by the adjusted *p*-value< 0.05 at a fold change (FC) score estimated by treatment *versus* control gene expression data. To quantify the FC of a given gene, we used the following notations: x = log_2_ (mean of normalized microarray hybridization signal value measured after treatment), y = log_2_ (mean value of normalized microarray hybridization signal value in control samples). If a gene is upregulated, then x > y and FC = x/y > 1; if a gene is downregulated, then y > x and FC = x/y< 1. We identified DEGs of microarray data by applying linear models and empirical Bayesian statistics of the limma package (99) in R. Significant DEGs were selected according to adjusted *p*-value< 0.05 after false discovery rate (FDR) correction. Additionally, significantly upregulated and downregulated DEGs were established based on the FC cut-off values, for example, -3.0 > log_2_FC > 2.5.

### TCGA and pan-cancer analysis of whole genomes consortium BRCA cohort data

#### RNA-seq gene expression dataset processing

Briefly, we analyzed log_2_ mRNA expression data for 1080 TCGA BRCA samples using the RSEM tool, which is an RNA-seq isoform quantification software summarizing transcript expression levels either as transcripts per million (TPM), reads per kilobase of transcript per million reads mapped (RPKM), or fragments per kilobase of transcript per million reads mapped (FPKM). RSEM converted the RNA-seq raw data for the selected samples. Batch normalization was based on Illumina HiSeq_RNASeqV2 (84). Before analysis, log_2_(x+1) transformed the upper quartile–normalized RSEM data for batch-corrected mRNA gene expression.

#### Gene-level copy number variation quantification

For 1080 breast carcinomas, we used the categorized gene-level CNV defined by the GISTIC2 method (54). We used the experimentally measured copy number profile using the Affymetrix Genome-Wide Human SNP Array 6.0 platform at the TCGA genome characterization center. We used the segment copy number data defined by GISTIC2 segments mapped to the hg19 genome assembly. Genes are mapped onto the human genome coordinates using the UCSC Xena HUGO probeMap. We applied the GISTIC2 method using the TCGA Firehose pipeline, which produces gene-level copy number categorized values of {-2, -1, 0, 1, 2}. These values categorize the tumor based on homozygous deletion, single copy deletion, regular diploid copy, low-level copy number amplification, or high-level copy number amplification.

#### TCGA protein expression data

We used the expression data of 119 proteins and phosphoproteins detected by the reverse-phase protein array (RPPA) platform (937 cancer tissue samples). This dataset was generated and processed at the MD Anderson Cancer Center RRPA Core Facility. In addition, we downloaded the level 3 interpreted level dataset from cBioPortal (https://www.cbioportal.org/datasets).

#### BRCA subtypes and datasets for survival analysis

For our recurrent-free survival (RFS) and overall survival outcome prediction analyses, we filtered initial TCGA BRCA tumor samples based on the detailed cancer type classification. We retained only the luminal A, luminal B, HER2, and BL ‘breast invasive ductal carcinoma’ categories (*n* = 780 cases). We also analyzed the dataset with the ‘infiltrating lobular carcinoma’ and ‘infiltrating ductal carcinoma’ categories (*n* = 972). We only used luminal A, luminal B, HER2, and BL subtype samples with information regarding RFS (*n* = 848). We also observed that for a significant proportion of patients in TCGA, the BRCA molecular subtypes reported by different TCGA teams are distinct. Therefore, in our analysis regarding BRCA subtypes, we selected the samples whose subtype classification was not contradictory.

*CNAs.* We observed KDM7A-DT CNA data for 991 patients (PCAWG cohort) categorized as amplification (n = 84), gain (n = 222), shallow deletion (n = 95), and deep depletion (n = 0) cases.

### Gene expression omnibus microarray datasets

We downloaded probe set expression values from microarray experiments of cohorts of patients with BRCA from the NCBI GEO repository (https://www.ncbi.nlm.nih.gov/geo/), using the GEOquery package in R (100) available through Bioconductor. The GEO accession numbers of the cohorts used are GSE1456 (Stockholm cohort), GSE4922 (Uppsala cohort), and GSE61304 (BII-OriGene Singapore cohort). We analyzed the three cohorts separately.

### Gene set enrichment analysis

GSEA (101) aggregates the per-gene statistics across genes within a gene set (using FC for each gene), making it possible to detect situations where all genes in a predefined set change in a small but coordinated way. Thus, before conducting GSEA, we sorted all datasets in decreasing order regarding log2FC. We analyzed this list using the clusterProfiler package in R, with an adjusted *p*-value cut-off set at 0.05 and subsequently performed GSEA against the cancer hallmark gene sets of the Molecular Signatures Database (MsigDB, version 7.5.1; https://www.gsea-msigdb.org/gsea/msigdb/index.jsp) using the msigdb r package. We visualized enrichment results using a function from the clusterProfiler package (102). We subsequently used Cytoscape (103) to construct custom networks.

### Statistical tests and software

#### Histogram symmetry test for analysis of skewed frequency distributions of gene alteration events

The discrete frequency distribution is *symmetric* if we have approximately the same shape on either side of the histogram’s middle. Alternatively, a skewed (non-symmetric) frequency distribution (histogram) has no mirror imaging of the frequencies. For skewed distributions, it is expected to have one tail of the distribution considerably longer or drawn out relative to the other. Suppose that N samples with discrete random values {-2, -1, 0 1, 2} are used to construct a histogram. Our algorithm randomly drew two similar-sized samples $n_{1}$ and $n_{2},$ where $n_{2}=N-n_{1}$ and $\min\left|n_{1}-n_{2}\right|\leq 1$ . For the first subset of size $n_{1}$ , the algorithm constructs the frequency distribution using the original random variable values {-2, -1, 0 1, 2}; for the second subset, the algorithm assigns the frequency values calculated to mirror-imaging random variable values {2, 1, 0, -1, -2}. Next, we consider a null hypothesis, assuming that these two distributions are paired distributions belonging to the same distribution. Our algorithm applies an exact similarity paired distribution test to calculate the two-sided *p*-value (Cytel Studio-12). Using such a sampling and testing procedure m -times ( m >1000 or a large enough number) allows for estimating the mean value and confidence intervals of the sampling distribution *p*-values. We used Sigma Plot-12 graphical tools in this study.

### Image analysis

We performed image analysis using ImageJ software (https://imagej.nih.gov/ij/) and normalized the signal values by dividing them by the pixel’s nucleus area and multiplying them by 100.

## Data availability statement

The datasets presented in this study can be found in online repositories. The names of the repository/repositories and accession number(s) can be found in the article/**Supplementary Material**. All data from this study are available in the supporting materials; Illumina Human HT-12 v4 Expression BeadChip data are deposited in the Array Express database (www.ebi.ac.uk/arrayexpress) under accession numbers E-MTAB-11818 (for the T47D cell line data) and E-MTAB-11819 (for the MRC5 cell line data).

## Ethics statement

Ethical approval was not required for the studies on humans in accordance with the local legislation and institutional requirements because only commercially available established cell lines were used.

## Author contributions

AG. and VAK conceived the study and designed the research. AG and MYA performed the experiments. VAK, AG, MT, VG, GSO, CP, and AVI performed data curation, bioinformatics, and data analysis. VAK and AG. contributed materials, reagents, metadata, and analytical tools. AG and VAK wrote the original draft and the final version of the manuscript. All authors have agreed to the published version of the manuscript.

## Glossary

**Table:**

1-D DDg	one‐dimensional data‐driven grouping
2-D DDg	one‐dimensional data‐driven grouping
AS	aneuploidy score
ASO	antisense oligonucleotide
BCA	bicinchoninic acid
BRCA	breast cancer
BLBC	basal-like breast cancer
CSR	core stress response
CNA	copy number alteration
DDR/R	DNA damage response and repair
DD RNA	DNA damage response RNA
DEG	Differentially expressed gene
DFS	disease-free survival
dilncRNA	damage-induced long noncoding RNA
DSB	double-strand break
dsRNA	double-stranded RNA
EMT	Epithelial-mesenchymal transition
ER	estrogen receptor
FBS	fetal bovine serum
FGA	fraction genome altered
GSEA	Gene Set Enrichment Analysis
H_2_O_2_	hydrogen peroxide
HR	homologous recombination
HRD	homologous recombination defects
IN	indel neoantigen
IPA	ingenuity pathway analysis
ITI	invasive tumor index
ITG	intratumor heterogeneity
IS-ncRNA	Intermediate-size ncRNA
KEGG	Kyoto Encyclopedia of Genes and Genomes
LNA	locked nucleic acid
LF	leucocyte fraction
NHEJ	Non-homologous end joining
OD	optical density
OS	oxidative stress
PBS	phosphate-buffered saline
RFS	Recurrence-free survival
PPI	protein-protein interaction
PS	proliferation scores
DFS	disease-free survival
PDR	protein damage response
PS	proliferation score
TP53 MMI	*TP53* missense mutation index
MET	mesenchymal-epithelial transition
MM	missense mutations
MS	mutation score
NM	nonsense mutations
si-paancRNA	stress-induced promoter-associated antisense lncRNA
RACE	rapid amplification of cDNA ends
Ragnum HS	Ragnum cancer hypoxia score ROS, reactive oxygen species
RNP	ribonucleoprotein
RNS	reactive nitrogen species
SNV	single nucleotide variant
SSB	single-strand break
WHS	wound healing score
