# Supplementary material for: KDM7A-DT induces genotoxic stress, tumorigenesis, and progression of p53 missense mutation-associated invasive breast cancer
Source: Front Oncol. 2024 May 2;14:1227151. doi: 10.3389/fonc.2024.1227151 (PMC11097164; doi:10.3389/fonc.2024.1227151)
Supplement: Supplementary file 2 [file DataSheet_2.docx]

**Supplementary Information**

**Supplementary Methods**

**Reagents**

ABI Prism 7900 Sequence Detection System: Applied Biosystems

Agilent 2100 Bioanalyzer system: Agilent Technologies

anti-mouse IgG conjugated to HRP: Santa Cruz Biotechnology

anti-rabbit IgG, horseradish peroxidase (HRP)-linked: Santa Cruz Biotechnology

antisense oligonucleotides (#392, #424, #457): IDT Technologies

Axon GenePix 4000B scanner: Molecular Devices

BD FACSCalibur: Becton Dickinson

bovine serum albumin (BSA): Sigma-Aldrich

Click-iT EdU Alexa Fluor 647 Flow Cytometry assay: Invitrogen

donkey anti-mouse IgG conjugated to Alexa Fluor 594: Invitrogen, catalog no. A21203

Eagle’s minimum essential medium: opti-MEM/Gibco

ECL plus Western Blotting Detection Reagents: GE Healthcare

FCS: Life Technologies

FirstChoice® RLM-RACE Kit: Life Technologies

GeneRacer™ Kit: Life Technologies

goat anti-mouse IgG conjugated to Alexa Fluor 488: Invitrogen, catalog no. A11029

goat anti-rabbit IgG conjugated to Alexa Fluor 488: Invitrogen, catalog no. A11008

Illumina HumanHT-12 v4 Expression BeadChip: Illumina

Kodak x-omat 1000A Film Processor: Kodak

LNA gapmer sequences: Exiqon

LSM 710 confocal laser scanning microscope: Carl Zeiss

MessageAmp II aRNA Amplification kit: Ambion

mini-trans-blot electrophoretic transfer cell system: Bio-Rad Laboratories

monoclonal anti-α-tubulin antibody: DM1A, Sigma-Aldrich

mouse anti-BRCA1: Santa Cruz Biotechnology, catalog no. sc-6954

mouse anti-p53: Santa Cruz Biotechnology, catalog no. DO-1

mouse anti-phospho-ATM: Santa Cruz Biotechnology, catalog no. sc-47739

negative control LNA gapmer: Exiqon, catalogue no. 300610

nylon membrane: Ambion

pCDH-EF1-MCS-(PGK-GFP-T2A-Puro): System Biosciences
Lenti-X 293T: Clontech

PCR clean-up system: Qiagen

pMDLg/pRRE: Addgene

Polyvinylidene fluoride (PVDF) membranes: Millipore

propidium iodide: Sigma-Aldrich

pRSV-Rev: Addgene

pVSVG: Addgene

QIAGEN maxi prep: QIAGEN

rabbit ani-p21 Waf1/Cip1 (12D1): Cell Signaling Technology, Inc., catalog no. 2947

rabbit anti-53BP1: Novus Biologicals, catalog no. NB100-904

rabbit anti-phospho-histone H2A. X: Cell Signaling Technology, Inc., catalog no. 9718S

rabbit anti-RAD51: Santa Cruz Biotechnology, catalog no. sc-8349

RNase-free DNase I: Invitrogen

Superscript II First-Strand Synthesis Kit for RT-PCR: Invitrogen

SYBR Green PCR Supermix: Invitrogen

TA cloning vector pCR^®^ 2.1 TOPO: Life Technologies

TRIzol: Invitrogen

vanadyl ribonucleoside complex (VRC): New England Biolabs

VECTASHIELD reagent: Vector Laboratories

**Websites/databases/software**

Archs4: https://www.nih-cfde.org/product/archs4/

cBioportal: <https://www.cbioportal.org/datasets> [1]

clusterProfiler package: <https://bioconductor.org/packages/release/bioc/html/clusterProfiler.html>

Cytoscape: https://cytoscape.org/;[2] GEOquery package: [3]

EMTome: <http://www>.emtome.org/

Enrichr: https://maayanlab.cloud/Enrichr/

Illumina GenomeStudio: <https://www.illumina.com/techniques/microarrays/array-data-analysis-experimental-design/genomestudio.html>

ImageJ software: https://imagej.nih.gov/ij/

Ingenuity Pathway Analysis: <https://apps.ingenuity.com/ingsso/login>

Kyoto Encyclopedia of Genes and Genomes (KEGG): <https://www.genome.jp/kegg/kegg1.html>

Molecular Signatures Database (MsigDB, version 7.5.1): <https://www.gsea-sigdb.org/gsea/msigdb/index.jsp>)

NCBI GEO repository: [https://www.ncbi.nlm.nih.gov/geo/](https://www.ncbi.nlm.nih.gov/geo/))

One-dimensional data-driven grouping (1-D DDg): [4-6]

PANTHER: https://www.pantherdb.org

Sigma-Plot 12 graphical tools: https://sigmaplot.software.informer.com/12.0/

STRING-v.11.5: <https://string-db.org/>

survival package: version 3.3-1, <https://CRAN.R-project.org/package=survival>

survminer package: version 0.4.9; <https://cran.r-project.org/web/packages/survminer/index.html>

Two-dimensional data-driven grouping (2-D DDg): [5]

WIN-MDI 2.9 free software: [http://www.cyto.purdue.edu/flowcyt/software/Winmdi.htm](http://www.cyto.purdue.edu/flowcyt/software/Winmdi.html)
ZEN 2008 software: Carl Zeiss, catalog no. 410136-1054-110

**Functional Enrichment Analysis**

For our functional enrichment analysis of the selected DEG subsets, we performed overrepresentation analysis (ORA) regarding terms of Gene Ontology (biological processes) and Kyoto Encyclopedia of Genes and Genomes (KEGG) using the cluster profile [7] package in R. ORA is a widely used approach to determine whether known biological functions or processes are overrepresented (enriched) in an experimentally derived gene subset – for example, a subset of DEGs. In our functional enrichment analyses of DEGs for MRC5 cells, we used the gene (and protein) sets with minimum and maximum elements from 50 to 500 genes accordingly; we applied FDR correction for gene set *p*-values. When analyzing microarray expression data for T47D cells, we identified the subsets of DEGs following the procedure mentioned above for four groups: group A, stressed (treated with H_2_O_2_) compared with untreated; group b, *KDM7A-DT* knockdown compared with untreated; group C, stressed (treated with H_2_O_2_) *KDM7A-DT* knockdown compared with untreated *KDM7A-DT* knockdown; and group D, stressed (treated with H_2_O_2_) *KDM7A-DT* knockdown compared with stressed (treated with H_2_O_2_). For these groups, we tested significantly (*p* < 0.05) upregulated (FC > 1.2) and downregulated (FC < 0.8) DEGs for overrepresentation in GO terms using the clusterProfiler package. Furthermore, we grouped the enrichment analysis results into higher GO levels using ClueGo in Cytoscape. In addition, to obtain unbiased and specific results, we carried out protein-protein interaction (PPI) network, pathway, and gene subset functional enrichment analyses using STRING-v.11.5 (<https://string-db.org/>) and Ingenuity Pathway Analysis (<https://apps.ingenuity.com/ingsso/login>).

## Gene Expression Omnibus (GEO) microarray datasets

Using standard normalized, batch effect correction, and filtering data algorithms, we also organized our metadata of six Affymetrix U133A and U133B expression arrays and the GEO datasets GSE21653 (Marseille cohort, *n* = 243), GSE6532 (GUYT data, *n* = 68), GSE9195 (GUYT_2 data, *n* = 58), GSE61304 (Singapore cohort [BII-OriGene]; *n* = 57), GSE6532 (Oxford cohort, *n* = 116), and GSE19615 (Manhattan cohort, *n* = 103). Our metadata includes 650 BC expression profiles and essential clinical factors, including histologic grade, estrogen receptor (ER) and progesterone receptor (PGR) status, tumor subtype, and survival data. Standard batch effect correction and log_2_-normalized expression, outlier exclusion, and statistical quality control tests have been published previously. We extracted log_2_-normalized expression for each probe set. We defined gene expression by selecting the corresponding probe set with the highest average value across samples compared to the other probe sets related to a particular gene.

## TCGA and Pan-Cancer Analysis of Whole Genomes (PCAWG) Consortium BC cohort data

*TCGA.* We downloaded the breast invasive carcinoma dataset (TCGA, PanCancer Atlas) from cBioPortal [8]. Clinical information was available for 1084 samples. In addition, somatic mutation information was available for 1009 tumors and matched normal samples. These somatic variants (simple nuclear polymorphisms (SNPs) and small indels) were identified following whole-exome sequencing using MuTect2. The dataset for our mutation analysis includes 130,495 mutation variants, defined by 17 variant classes; the most abundant class was missense mutations, consisting of 51% (66,930/130,495) events.

*PCAWG.* We downloaded the PCAWG Consortium, TCGA BC CNV, transcriptional profiles, and clinical datasets from cBioPortal (<https://www.cbioportal.org/study/summary?id=pancan_pcawg_2020>) [9]. The 2583 tumor samples represented 46 cancer types.

## Survival analysis

We evaluated the ability of *KDM7A-DT* to discriminate the studied patients into two distinct groups based on their tumor sample gene expression levels and survival outcome (event and last follow-up time or time of event after diagnosis) by using KMplot [10], which utilizes the survival package for multivariate and univariate Kaplan-Meier Analysis. The algorithm splits the patients into two groups based on the optimized gene expression level cut-off value. It then computes a *p*-value that corresponds to the statistic of the slope coefficient of the Cox proportional hazards model after goodness-of-fit, fitting the latter to grouped survival data (Split patients by “best cutoff”). The cut-off value that results in the minimum *p*-value and permits the minimum number of samples in the groups is considered optimal. After defining the optimal cut-off, patient samples into two relatively high- and low-risk categories regarding the expression level of the gene (e.g., *KDM7A-DT*) (either low or high expression). The datasets used in the analysis comprised a total of 2032 patients from 50 cohorts (E-MTAB-365, E-TABM-43, GSE11121, GSE12093, GSE12276, GSE1456, GSE16391, GSE16446, GSE16716, GSE17705, GSE17907, GSE18728, GSE19615, GSE20194, GSE20271, GSE2034, GSE20685, GSE20711, GSE21653, GSE22093, GSE25066, GSE2603, GSE26971, GSE29044, GSE2990, GSE31448, GSE31519, GSE32646, GSE3494, GSE36771, GSE37946, GSE41998, GSE42568, GSE43358, GSE43365, GSE45255, GSE4611, GSE46184, GSE48390, GSE50948, GSE5327, GSE58812, GSE61304, GSE65194, GSE6532, GSE69031, GSE7390, GSE76275, GSE78958, GSE9195). We used this grouping to perform survival prediction analysis on clinical data (RFS, OS, DMFS), to estimate the corresponding risks of individual groups, and to conclude whether they differed statistically by conducting a log-rank test between the groups [4-6, 10].

**STRING enrichment analysis**

For STRING enrichment analysis, we selected DEGs using relatively more robust statistical criteria: adjusted *p*-value < 0.05 and FC > 1.35 for upregulated DEGs and FC < 0.74 for downregulated DEGs. We selected 665 upregulated DEGs and 680 downregulated DEGs. Up-regulated genes were found highly enriched in gene set ‘ribosome biogenesis and RNA helicase activity’ (*n* = 55 of 200, *p* = 1.22E-27, STRING CL:735) and several specific GO terms, including ‘regulation of transcription by RNA polymerase II’ (*n* = 116, *p* = 1.29E-6; GO:0006357) and ‘chromosome organization’ (*n* = 68 of 1066, *p* = 5.4E-6, GO:0051276). The top enriched GO terms were represented by the following gene sets: ‘cell adhesion’ (*n* = 93 of 925, *p* = 9.2E-17; GO:0007155), ’'regulation of cell migration’ (*n* = 85 of 865, *p* = 9.8E-15; GO:0030334), ‘integrin-binding’ (*n* = 32 of 147, *p* = 6.4E-12, GO:0005178), ‘collagen-containing extracellular matrix’ (*n* = 56 of 538, *p* = 1.02E-24; GO:0050839), ‘anchoring junction’ (*n* = 87 of 820, *p* = 2.72E-18; GO:0070161), ‘focal adhesion’ (*n* = 67 of 405, *p* = 6.1E-23; GO:0005925), and ‘glycoprotein’ (*n* = 260 of 4349, *p* = 1.8E-21; KW-0325). Among others, the most significant GO term, 'extracellular vesicles,’ collected 201 of 680 downregulated genes (n = 201 of 2121, p = 1.65E-39; GO:0070062). These findings imply that genes coregulated with KDM7A-DT are involved in the positive regulation of transcription activity, RNA biogenesis, intercellular communication, metabolism, inflammation response, and apoptosis.

**GSEA hallmark gene sets analysis: DEG comparison of the KDM7A-DT knockdown in T47D with the KDM7A-DT overexpression in nonmalignant fibroblasts**

We compared the enriched gene sets identified in the KDM7A-DT knockdown experiment in T47D cells with those obtained from KDM7A-DT overexpression in nonmalignant fibroblasts (MRC5 cells). Three pathways (inflammatory response, interferon-gamma response, and apoptosis) enriched with downregulated DEGs after KDM7A-DT overexpression due to KDM7A-DT amplification in the fibroblasts. The genes of these pathways were enriched with upregulated DEGs after full-length KDM7A-DT knockdown in BRCA T47D cells.

The perturbed KDM7A-DT expression revealed downregulated DEGs in T47D cells abundant in myogenesis, heme metabolism, and estrogen response pathways; upregulated DEGs were enriched in only one pathway (TNFα signaling via NF-κB). Note that three pathways, the interferon-alpha response, G2/M checkpoint, and oxidative phosphorylation, were inversely regulated by KDM7A-DT in MRC5 and T47D cell models (Figure 8C).

Interestingly, three pathways (inflammatory response, interferon-gamma response, and apoptosis) enriched with downregulated DEGs after KDM7A-DT overexpression due to KDM7A-DT amplification in the fibroblasts. The genes of these pathways were enriched with upregulated DEGs after full-length KDM7A-DT knockdown in BRCA T47D cells.

Moreover, we performed a correlation analysis of KDM7A-DT and mRNA expression profiles in T47D cell samples to evaluate ‘the combinatory effect’ of KDM7A-DT in the experimental data (Table S10). Comparing the DEGs in commonly affected pathways, we found 16 common genes in the interferon-alpha response (eight positively correlated and one negatively correlated), 23 common genes in the G2/M checkpoint (9 positively correlated and 4 negatively correlated), and 33 common genes in oxidative phosphorylation (18 positively correlated and 5 negatively correlated).

The comparative enrichment analysis of DEG expression suggest common and unique pathways in noncancer MRC5 fibroblast (MRC5) and BRCA cells (T47D); in both cell types, modulation of KDM7A-DT expression provides a global change in the expression profiles. In part, they showed similar trends of altered genes within the same pathways.


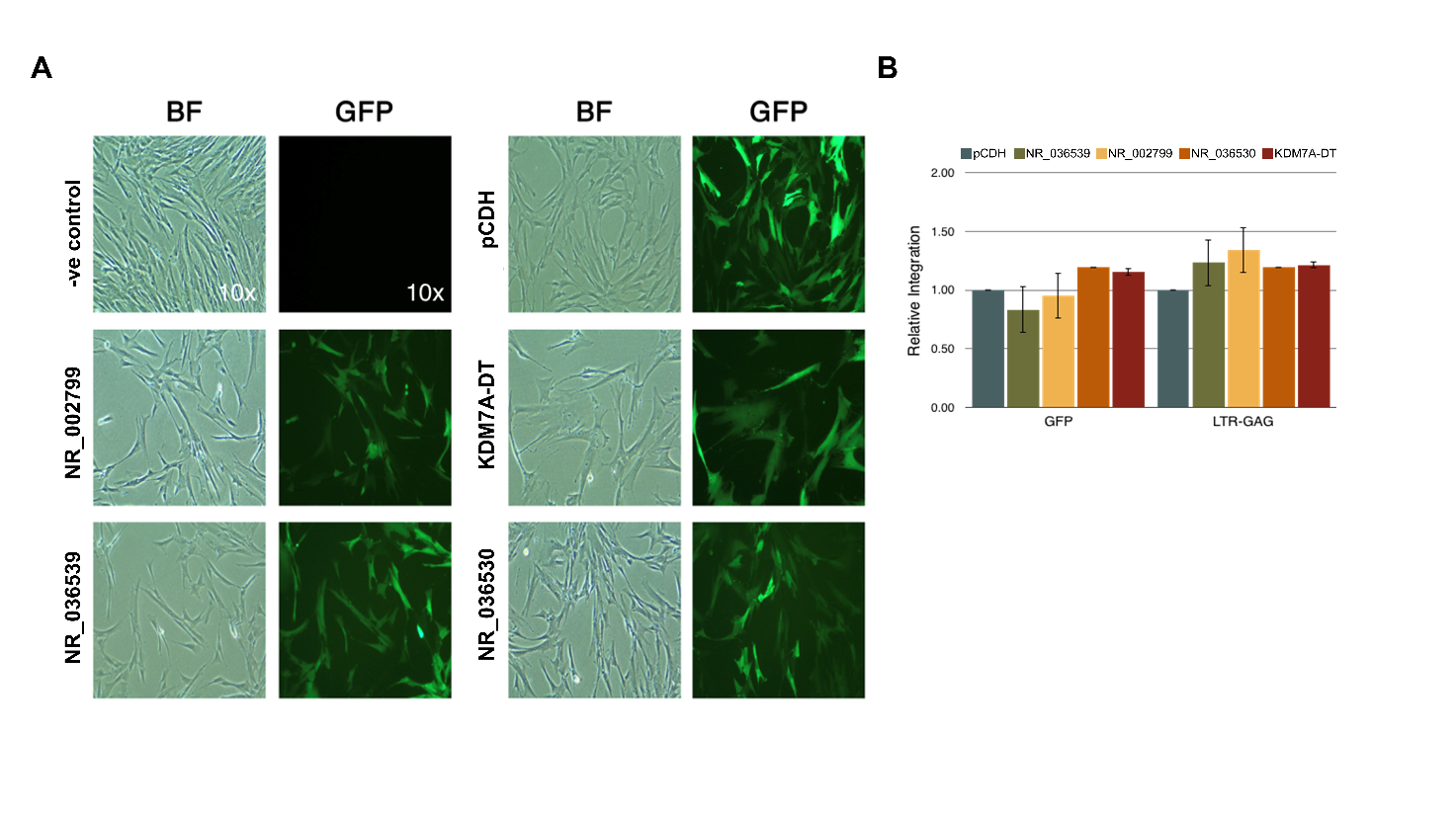
**Supplementary Figures**

**Figure S1.** Selected stress-induced paancRNAs critically affect the morphology of non-malignant cells. **A.** The morphology of MRC5 fibroblasts was significantly altered upon over-expression of all four selected si-paancRNAs compared to their normal phenotype. In brief, MRC5 control cells showed the expected small spindle morphology, which, upon over-expression of all si-paancRNAs, changed to a flattened and relatively enlarged shape. GFP fluorescence intensity was used to adjust the expression levels of all paancRNA clones to similar levels with the empty pCDH vector. **B.** Quantification of genomic integration of vector sequences, LTR-GAG transgene, and GFP by qRT-PCR. FACS and PCR confirmed titrations in target cell lines using specific primers for LTR-GAG: 5′- TGTGTGCCCGTCTGTTGTGTGAC-3′. 5′-TCGGGCGCCACTGCTAGA G-3′; and for GFP: 5′-AGAGCACCAAAGGCGCCCTG-3′; and 5′-TGCGGGTG TTGGTGTAGC-3′ to determine lentiviral genomic integration.


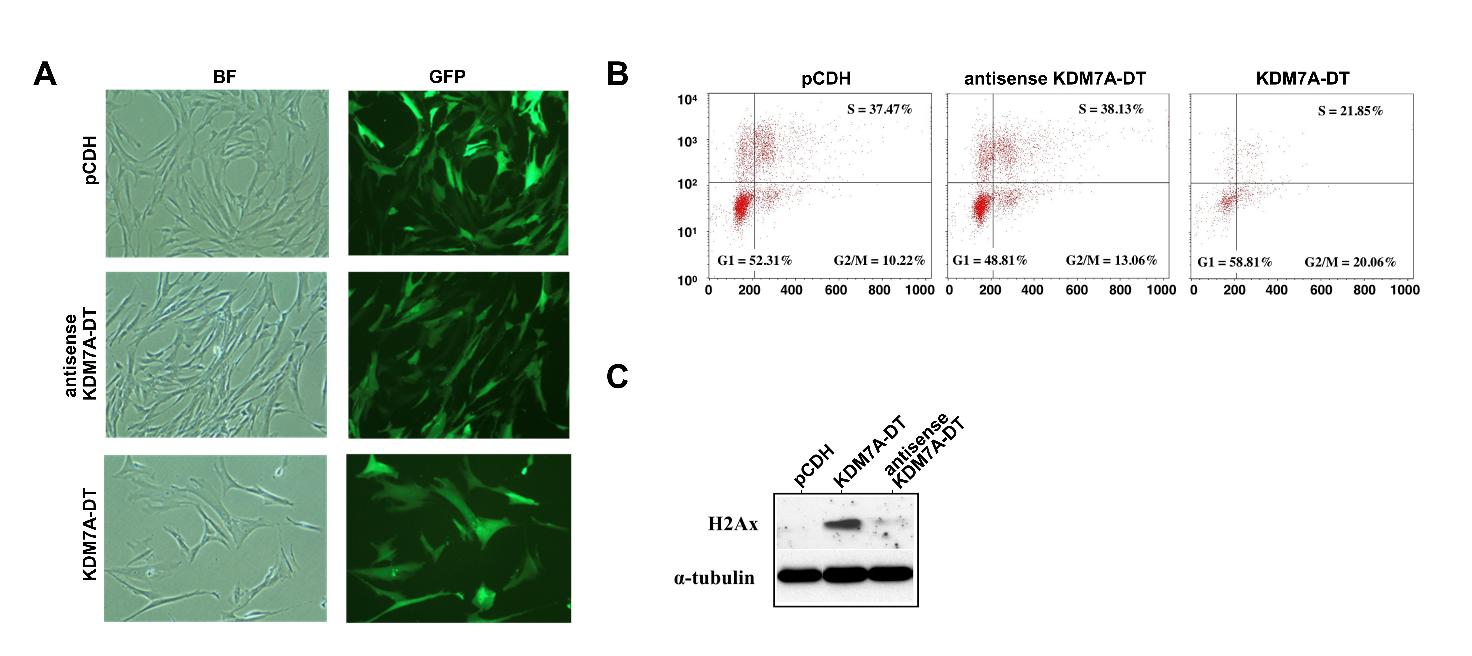


**Figure S2.** Strand specificity dictates KDM7A-DT regulatory function in the cell cycle, and DDR/R. **A.** MRC5 cells over-expressing the complementary strand of KDM7A-DT transcript (labeled as antisense KDM7A-DT) does not affect the morphology of normal fibroblast MRC5 compared to cells over-expressing just the vehicle control. In addition, the forced expression of *KDM7A-DT*’s complementary strand is not affected. **B.** normal cell cycle progression. **C.** phosphorylation levels of the H2AX protein compared to control cells.


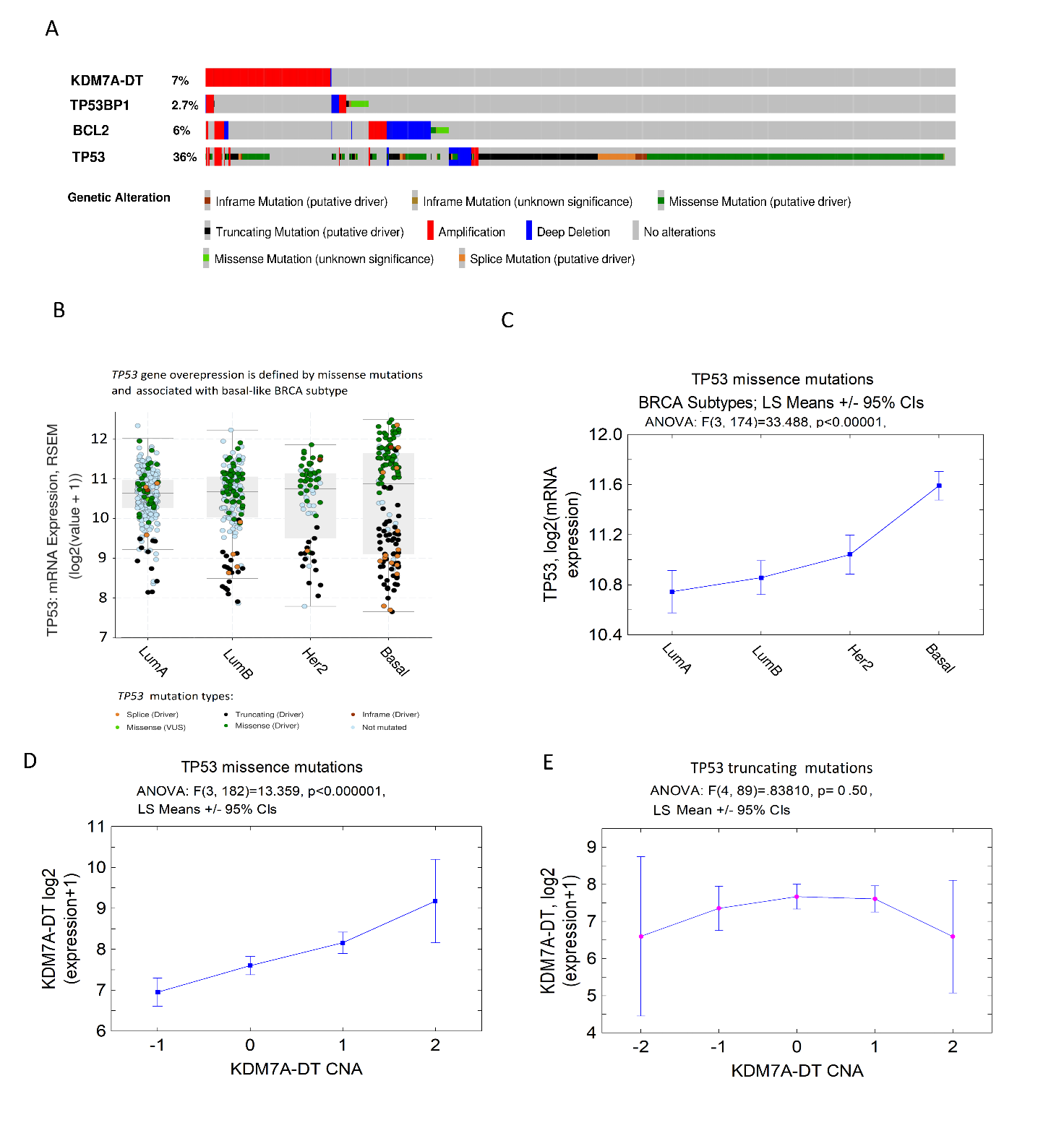


**Figure S3**. Genetic alterations (Copy number alterations (CNA) and significant classes of mutations). **A.** genes *KDM7A-DT*, *TP53BP1*, *BCL2,* and *TP53* were defined in 2,583 tumor samples represented by the Pan-Cancer Analysis of Whole Genomes (PCAWG) Consortium. Source data from UCSC Xena and ICGC Data Portal. In about 7.3% of cases (189/2583), the *KDM7A-DT* locus was amplified. The 2.7%, 6%, and 36% gene alterations were found for protein-coding genes *TP53BP1*, *BCL2,* and *TP53,* respectively. **B. C. D. E.** Association of *TP53* mutation types and expression patterns of KDM7A-DT and TP53 across BC subtypes and *KDM7A-DT* CNA categories (TCGA data have been analyzed; see Method for details).


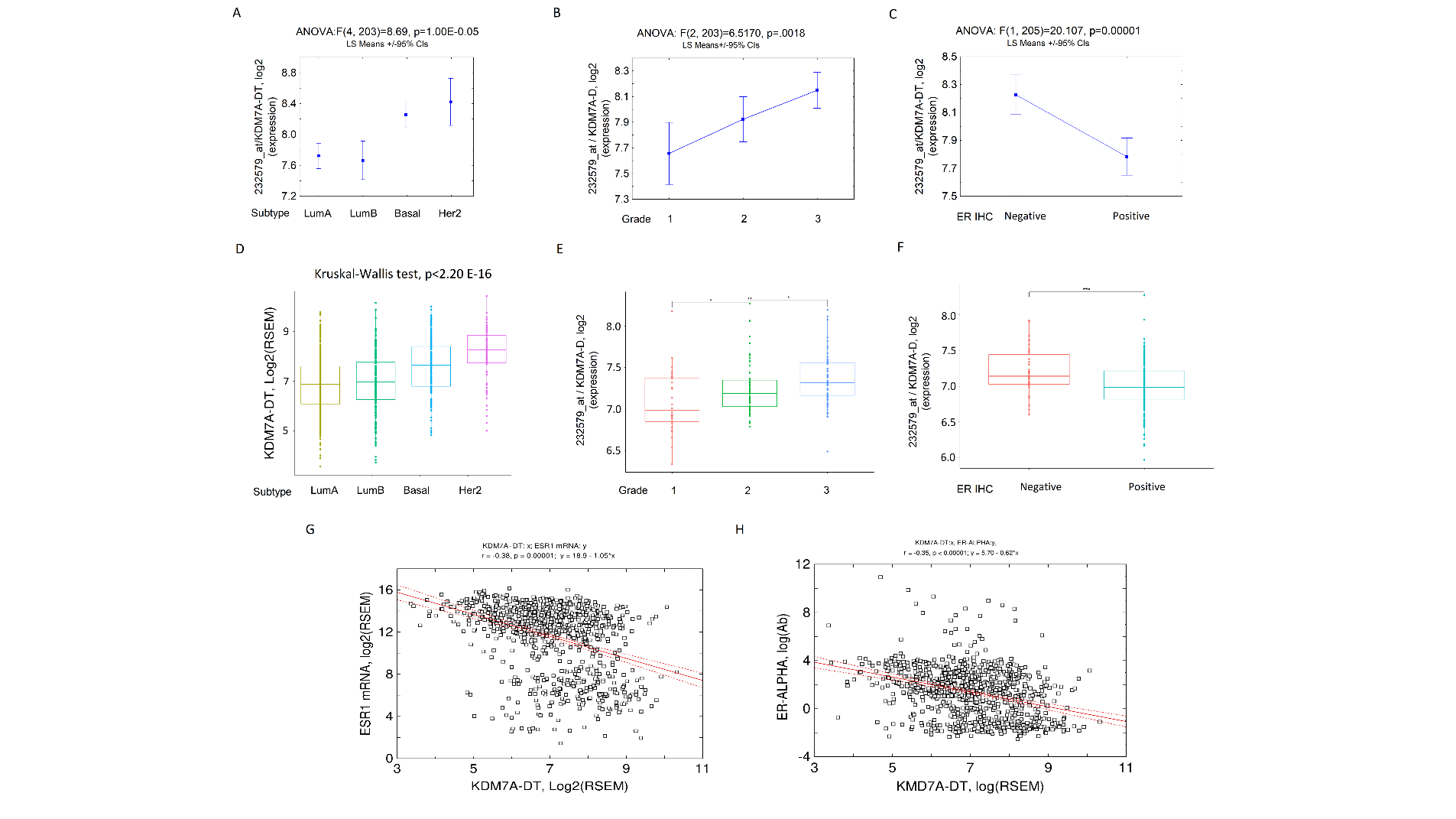


**Figure S4**. Association of KDM7A-DT expression with BC subtypes, clinical characteristics, estrogen receptor, **A. D.** Boxplots of *KDM7A-DT* expression across BC subtypes. **B. E.** *KDM7A-DT* expression is positively correlated with histological grade. **C. F.** *KDM7A-DT* expression is positively correlated with positive ER status. **G. H.** *KDM7A-DT* expression is negatively correlated with *ESR1* RNA and protein expression.


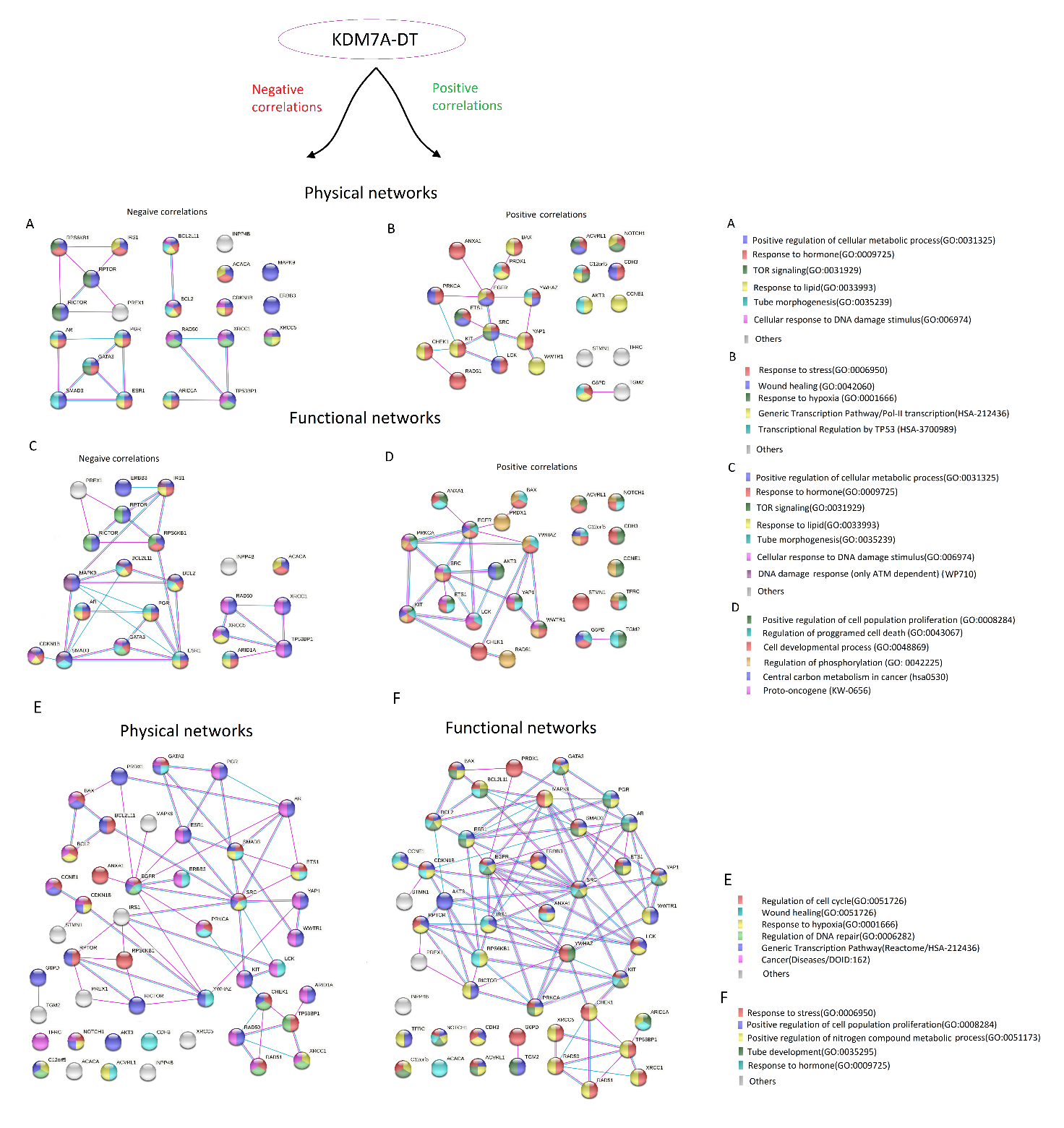


**Figure S5.** Protein-protein networks of 46 DDR proteins positively and negatively correlated with KDM7-DT RNA expression data were downloaded from sBioPortal (TCGA, 2018). The proteins have been selected as described in the Methods. Each protein expression is also correlated with corresponding mRNA levels of a DDR gene encoding these products. STRING 11.5 is used for annotation, network construction, and enrichment analysis. These results are concordant with our experimental data analyses.

**
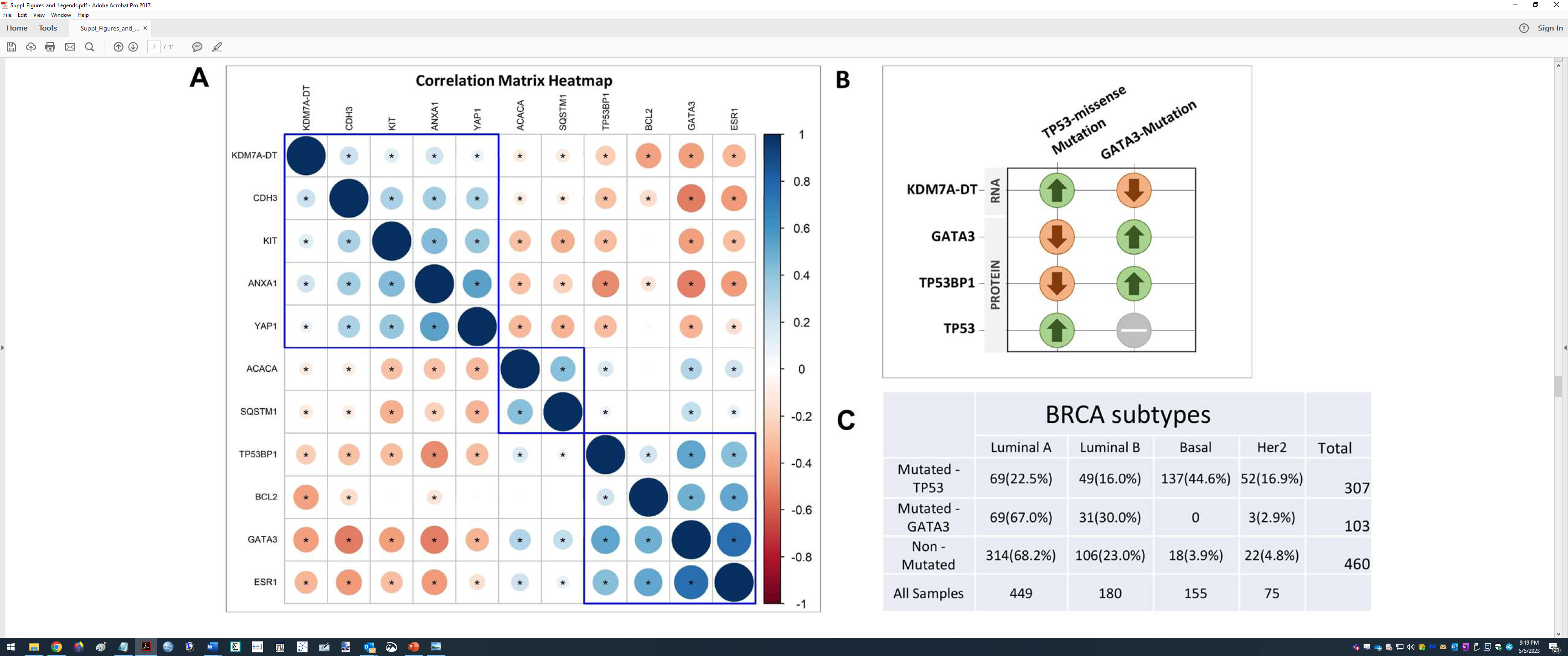
**

**Figure S6.** Mutations in *GATA3* and *TP53* affect the expression of *KDM7A-DT*. **A.** Heatmap of top 10 correlated proteins with *KDM7A-DT* expression. **B.** *KDM7A-DT* expression and GATA3, TP53BP1, p53 protein levels alterations in *TP53* and *GATA3* mutated samples compared to non-mutated samples. The green arrow represents a statistically significant increase, and the orange arrow represents a statistically significant decrease (p<0.05). **C.** *TP53* and *GATA3* mutated samples across breast cancer subtypes in the TCGA dataset.


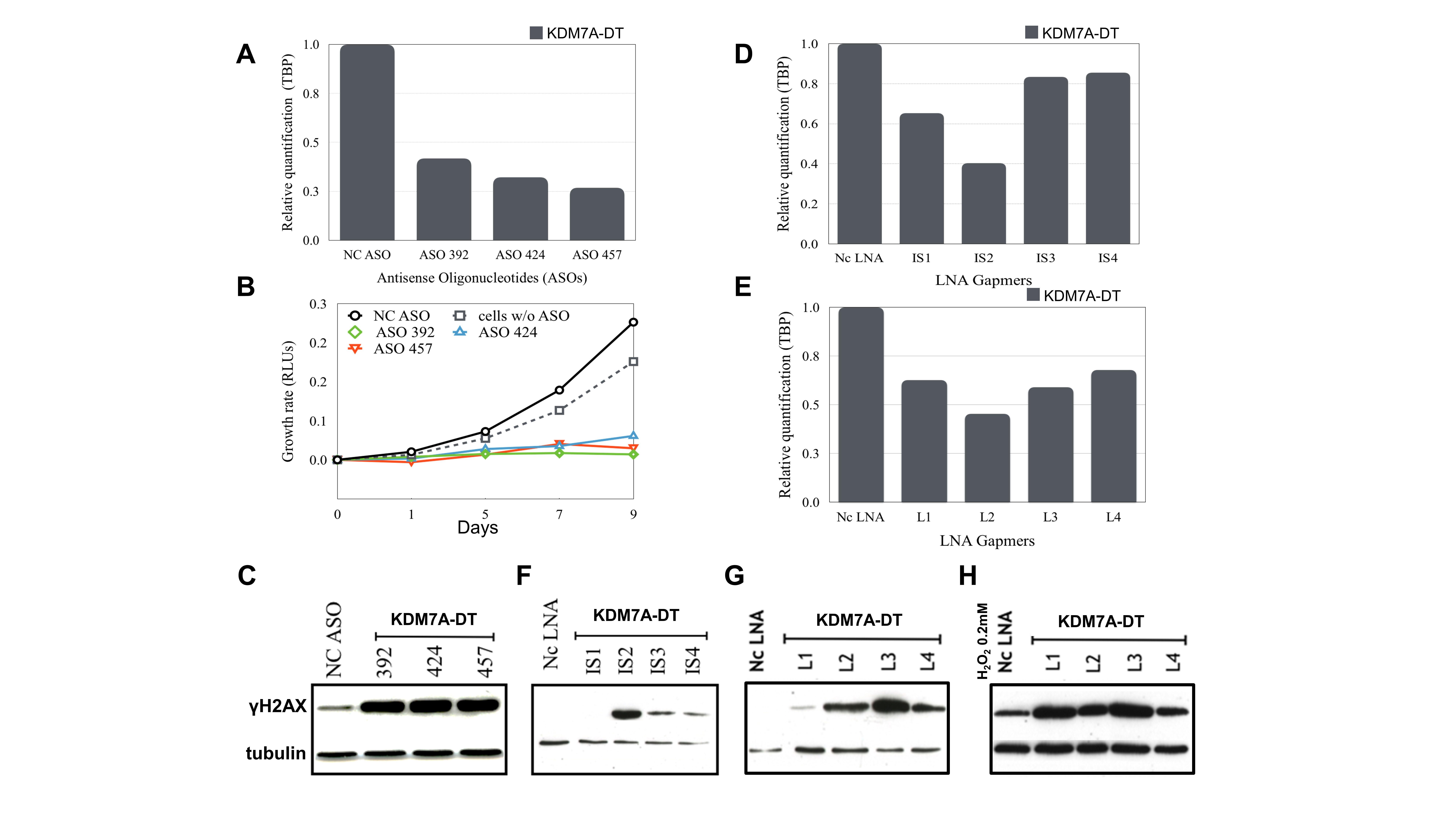


**Figure S7.** Knock-down of KDM7A-DT targeting either the transcript's start or end increases H2AX phosphorylation levels in T47D BC cells. **A**. Knock-down experiments with three different ASO gapmers targeting the beginning of KDM7A-DT significantly downregulate transcript levels compared to a control ASO (NC ASO), as quantified by RT-qPCR using a primer at the transcript’s end. **B.** All three different ASO gapmers significantly inhibit T47D cell viability compared to cells transfected with or without a control ASO. **C.** All three ASOs significantly increase the phosphorylation levels of H2AX protein compared to control ASO. **D.** Four different LNAs (IS1, IS2, IS3, IS4) targeting the beginning of *KDM7A-DT*, as analyzed by qPCR using a primer pair amplifying the end of KDM7A-DT transcript, compared to control LNA. **E.** Four LNAs (L1, L2, L3, and L4) target the transcript's end. **F.** Three out of four LNAs (IS2, IS3, IS4) targeting the beginning of the transcript modestly increase the phosphorylation of H2AX compared to control LNA. **G.** Three out of four LNAs (L1, L3, L4) targeting the end of the transcript demonstrated a significant increase in H2AX phosphorylation compared to the control LNA when cells. **G.** All LNAs (L1, L2, L3, L4), including the control LNA, exhibited a substantial elevation in H2AX phosphorylation under H_2_O_2_ treatment conditions.


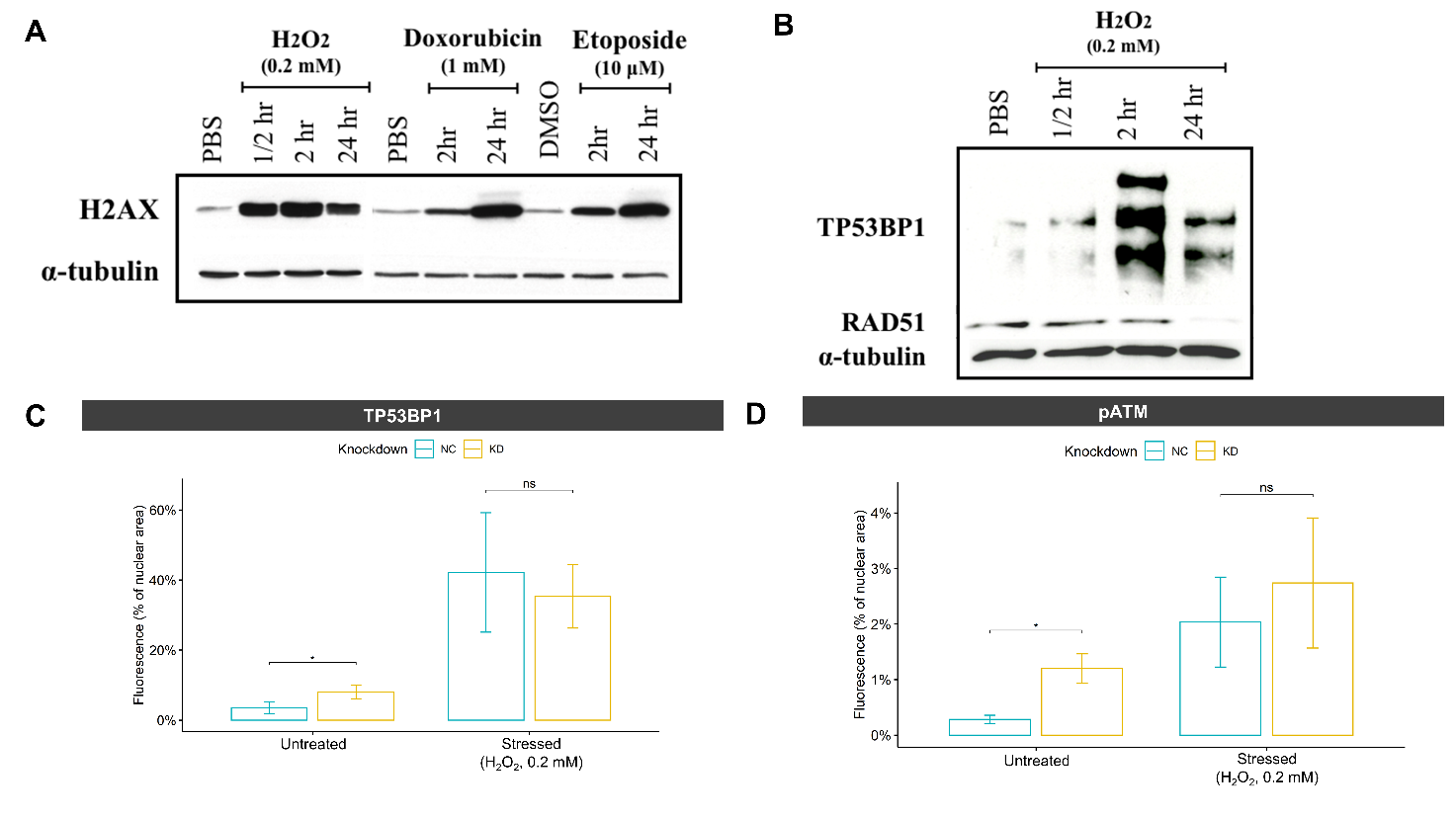


**Figure S8.** Oxidative stress induces H2AX phosphorylation and the DDR/R *via* the NHEJ pathway. **A.** OS (H_2_O_2_, 0.2 mM) is activating H2AX phosphorylation in T47D BC cells similar to classical DNA-damage inducers, such as Doxorubicin (1 mM) and Etoposide (10 μM). **B.** In T47D cells, the expression levels of TP53BP1 and RAD51 proteins are analyzed at three different time points (30 minutes, 2 hours, and 24 hours) following H_2_O_2_ treatment. In brief, RAD51 levels are downregulated at the 24-hour time-point (and remain unaffected at 30 minutes and 2 hours) compared to untreated cells. In contrast, TP53BP1 levels are sharply upregulated at 30 minutes, reaching a maximum at 2 hours and reducing at 24 hours. Three TP53BP1 protein isoforms occurred at 2 hr, while two isoforms occurred at 30 min and 24 hr. Therefore, the 30-minute time-point was selected for further analysis. A relative TP53BP1 expression (**C**) and a relative phosphorylated ATM level (D) in KDM7A-DT knock-down v.s. Control in a nuclear area T47D BC cell under untreated conditions and after 30 minutes of OS (H_2_O_2_, 0.2 mM) compared to control cells (Nc LNA).


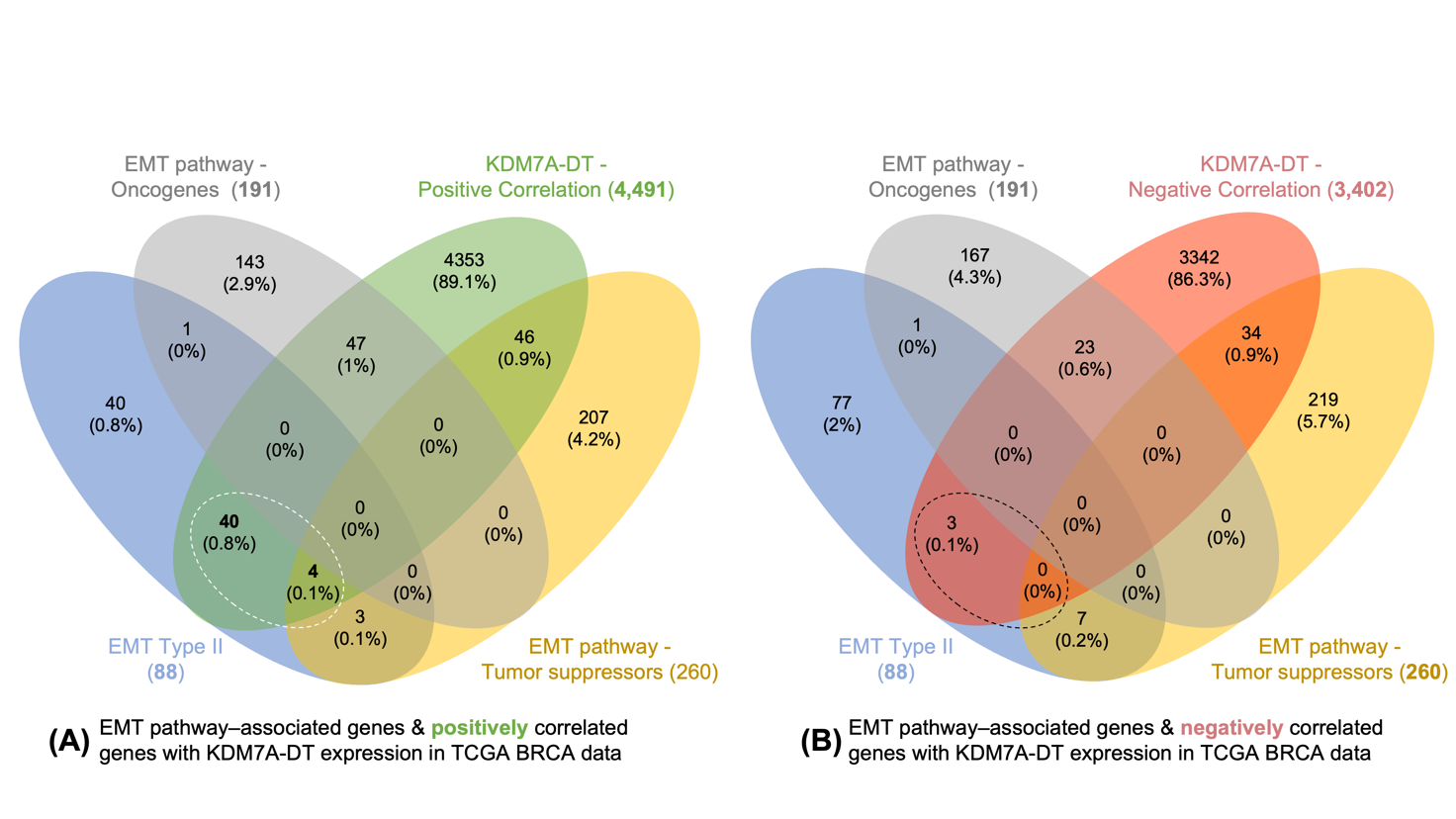


**Figure S9**. Venn Diagram Illustrating genes that are in (A) Positive Correlation (green) and (B) Negative Correlation (red) with KDM7A-DT Expression (TCGA BRCA Dataset). EMT Pathway-Associated Genes (Grey and Yellow) annotated as Oncogenes and Tumor Suppressors Using the dbEMT-2 Database. The EMT Type II group defined in this study (blue) (Table S3), is a subset of genes from the EMT Cancer Hallmark Pathway that were found to be suppressed under KDM7A-DT overexpression.
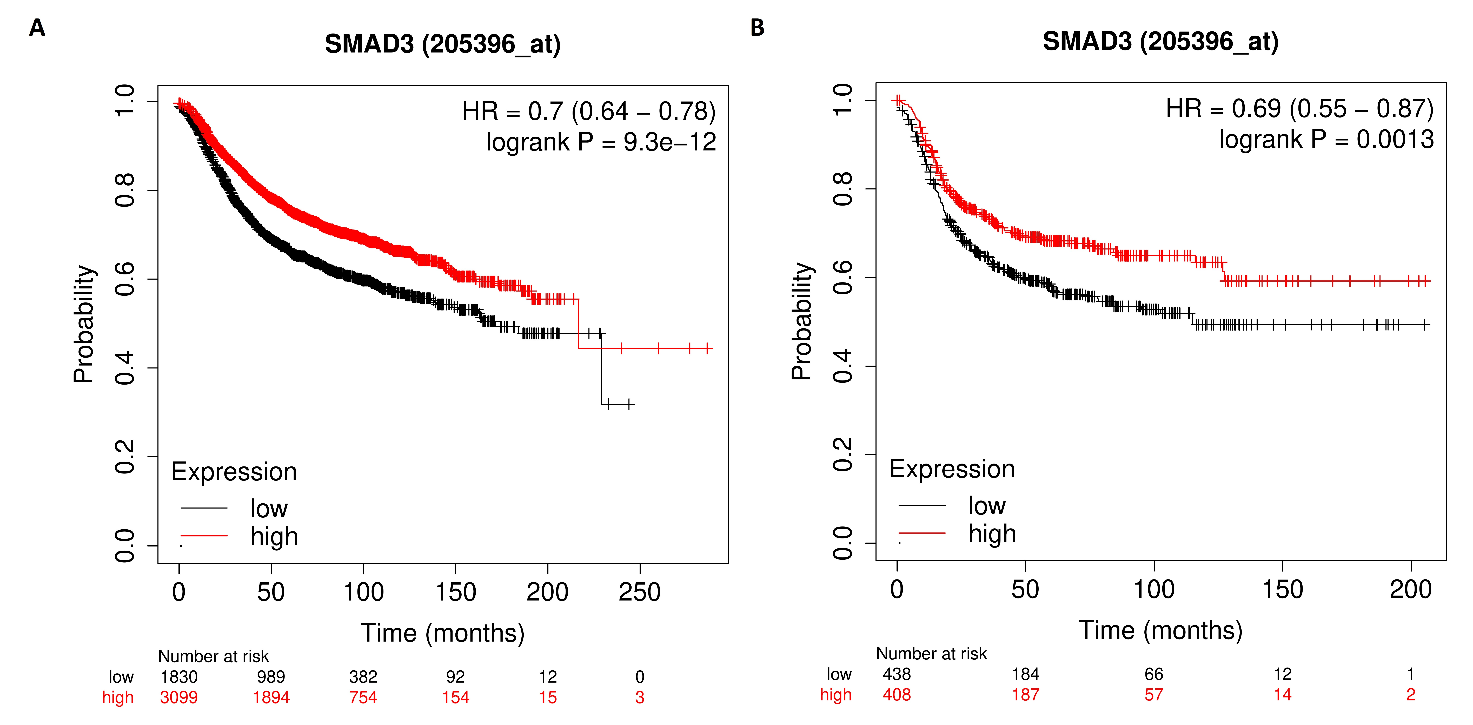


**Figure S10.**  Survival prediction analysis of BC patients defined by the mRNA level of tumor suppressor SMAD3 that expression was negatively correlated with KDM7A-DT expression. **A**. Affymetrix U133A microarray expression data of SMAD3 (probesets 205396_at), recurrence-free survival (RFS) time, and events for all BC 4,929 patients available from the Kaplan-Meier Plotter (K-M plotter) database **B.** The K-M survival functions for 446 basal BC patients. The functions were defined under the same conditions as for all patients.

**References**

1. Cerami, E., Gao, J., Dogrusoz, U., Gross, B. E., Sumer, S. O., Aksoy, B. A., Jacobsen, A., Byrne, C. J., Heuer, M. L., Larsson, E., Antipin, Y., Reva, B., Goldberg, A. P., Sander, C., & Schultz, N. (2012). The cBio cancer genomics portal: an open platform for exploring multidimensional cancer genomics data. Cancer discovery, 2(5), 401–404.
2. Shannon,P., Markiel,A., Ozier,O., Baliga,N.S., Wang,J.T., Ramage,D., Amin,N., Schwikowski,B. and Ideker,T. (2003) Cytoscape: a software environment for integrated models of biomolecular interaction networks. Genome Res., 13, 2498–2504.
3. Davis,S. and Meltzer,P.S. (2007) GEOquery: a bridge between the Gene Expression Omnibus (GEO) and BioConductor. Bioinformatics, 23, 1846–1847.
4. Motakis,E., Ivshina,A.V. and Kuznetsov,V.A. (2009) Data-driven approach to predict survival of cancer patients: estimation of microarray genes' prediction significance by Cox proportional hazard regression model. IEEE Eng. Med. Biol. Mag/, 28, 58–66.
5. Chen,L., Jenjaroenpun,P., Pillai,A.M., Ivshina,A.V., Ow,G.S., Efthimios,M., Zhiqun,T., Tan,T.Z., Lee,S.C., Rogers,K. et al. (2017) Transposon insertional mutagenesis in mice identifies human breast cancer susceptibility genes and signatures for stratification. Proc. Natl. Acad. Sci. U. S. A., 114, E2215–E2224.
6. Grinchuk,O.V., Yenamandra,S.P., Iyer,R., Singh,M., Lee,H.K., Lim,K.H., Chow,P.K.-H. and Kuznetsov,V.A. (2018) Tumor-adjacent tissue co-expression profile analysis reveals pro-oncogenic ribosomal gene signature for prognosis of resectable hepatocellular carcinoma. Mol. Oncol., 12, 89–113.
7. Yu G, Wang L, Han Y, He Q (2012). “clusterProfiler: an R package for comparing biological themes among gene clusters.” OMICS: A Journal of Integrative Biology, 16(5), 284-287.
8. Hoadley,K.A., Yau,C., Hinoue,T., Wolf,D.M., Lazar,A.J., Drill,E., Shen,R., Taylor,A.M., Cherniack,A.D., Thorsson,V. et al. (2018) Cell-of-origin patterns dominate the molecular classification of 10,000 tumors from 33 types of cancer. Cell, 173, 291–304.e296.
9. Campbell,P.J., Getz,G., Korbel,J.O., Stuart,J.M., Jennings,J.L., Stein,L.D., Perry,M.D., Nahal-Bose,H.K., Ouellette,B.F.F., Li,C.H. et al. (2020) Pan-cancer analysis of whole genomes. Nature, 578, 82–93.
10. Lánczky A, Győrffy B. Web-Based Survival Analysis Tool Tailored for Medical Research (KMplot): Development and Implementation. J Med Internet Res. 2021 Jul 26;23(7):e27633.
